# Gamma Responses to Colored Natural Stimuli Can Be Predicted from Local Low-Level Stimulus Features

**DOI:** 10.1523/ENEURO.0417-23.2024

**Published:** 2024-07-22

**Authors:** Sidrat Tasawoor Kanth, Supratim Ray

**Affiliations:** ^1^IISc Mathematics Initiative, Indian Institute of Science, Bangalore 560012, India; ^2^Center for Neuroscience, Indian Institute of Science, Bangalore 560012, India

**Keywords:** gamma, image-computable model, LFP, macaque, natural vision, V1

## Abstract

The role of gamma rhythm (30–80 Hz) in visual processing is debated; stimuli like gratings and hue patches generate strong gamma, but many natural images do not. Could image gamma responses be predicted by approximating images as gratings or hue patches? Surprisingly, this question remains unanswered, since the joint dependence of gamma on multiple features is poorly understood. We recorded local field potentials and electrocorticogram from two female monkeys while presenting natural images and parametric stimuli varying along several feature dimensions. Gamma responses to different grating/hue features were separable, allowing for a multiplicative model based on individual features. By fitting a hue patch to the image around the receptive field, this simple model could predict gamma responses to chromatic images across scales with reasonably high accuracy. Our results provide a simple “baseline” model to predict gamma from local image properties, against which more complex models of natural vision can be tested.

## Significance Statement

Oscillatory gamma response in the primate visual cortex is well studied for parametric stimuli like gratings and chromatic patches, but its emergence in response to naturalistic images is inconsistent, primarily due to differences in image features. We investigated the joint dependence of gamma response on a pair of stimulus features and found them to be independent, across multiple recording scales. We developed and validated a product-based model to predict response for parametric stimuli. By approximating images with parametric features and using the product-based model, we could predict gamma responses to complex chromatic natural images with reasonably high accuracy. Our results demonstrate a simple image-computable gamma response model based on low-level features.

## Introduction

Gamma rhythm (30–80 Hz) in the visual cortex has been related to high-level cognition (Fries et al., 2001; Womelsdorf et al., 2006), found to have high stimulus-related information (Kanth and Ray, 2020) and investigated as a marker for age-related cognitive decline (Murty et al., 2021). The dependence of gamma power and center frequency on parametric grating features like contrast, orientation, etc. has been well studied (Henrie and Shapley, 2005; Gieselmann and Thiele, 2008; Ray and Maunsell, 2010, 2011; Jia et al., 2013; Murty et al., 2018). Colored (especially reddish) stimuli have been discovered to elicit strong gamma responses in the local field potential (LFP) in the primary visual cortex (V1) of nonhuman primates (Shirhatti and Ray, 2018) and in human electrocorticogram (ECoG) recordings (Bartoli et al., 2019). Interestingly, while some studies have found strong gamma responses (N. Brunet et al., 2015) to naturalistic stimuli, others have reported weak or inconsistent gamma (Hermes et al., 2015), which could result from differences in image properties (N. M. Brunet and Fries, 2019).

This stimulus dependence of gamma has led to development of image-computable models which predict gamma responses to novel images. For instance, the orientation variance (OV) model (Hermes et al., 2019) developed using ECoG responses is based on the presence of orientated grating-like features in the receptive field (RF) detected using predetermined spatial Gabor filters, with the gamma response proportional to the variance in the filter outputs. Other studies have correlated gamma to uniform surfaces and the degree of structure (N. M. Brunet and Fries, 2019) or predictability in an image (Peter et al., 2019). Gamma has been correlated with the degree to which RF features can be predicted from the surrounding image (Uran et al., 2022). This model used naturalistic stimuli alone whereas the OV model used artificial stimuli. However, an efficient strategy is to build models using artificial stimuli and test them using natural images (Rust and Movshon, 2005).

We take a three-step approach using this strategy to build a simple image-computable model. The first step is to have a comprehensive knowledge of gamma tuning to as many features as possible. Typically, one feature is studied at a time but when two features are covaried, the responses can reveal how they are jointly represented in the brain. The interaction between tuning to different features can be characterized by their separability—to what degree the tuning to one feature is independent of the other. For completely separable interactions, the joint response can be constructed as a function of individual tunings. Separability analysis has been carried out for single neurons (Mazer et al., 2002; Smolyanskaya et al., 2013), membrane potentials (Peña and Konishi, 2001), and fMRI responses (Leoné et al., 2014). Some studies have found spatial frequency (SF) and orientation (Ori) tuning to be separable in monkeys (Mazer et al., 2002), while others have found interdependence of these features in mice (Ayzenshtat et al., 2016) and cat (Vidyasagar and Sigüenza, 1985) visual areas. Surprisingly, separability of LFP oscillations like gamma has not been well studied although they show preference to Ori and SF (Jia et al., 2013; Murty et al., 2018). Therefore, we first investigated the separability of gamma tuning to grating features and extended the analysis to HSV (hue, saturation, value) stimuli. Then, we fitted curves to individual feature responses and modeled the joint gamma response as a function of these tuning curves.

The second step involved decomposing a complex image into simple features with known gamma tuning. From the built models, we could estimate gamma for two classes of stimuli. The first had achromatic image patches that could be approximated by spatial Gabors. The second class contained colored images with areas of uniform luminance, hue, and saturation, which could be approximated as uniform chromatic patches. The final step involved applying the model learnt in the first step to the image parameters approximated in the second to get a prediction of the gamma response.

## Materials and Methods

### Animal preparation and recording

Two adult female monkeys (*Macaca radiata*; M1, 3.3 kg; M2, 4 kg) were used in this study. The experiments adhered to the guidelines of the Institutional Animal Ethics Committee (IAEC) of the Indian Institute of Science, Bangalore, and the Committee for the Purpose and Supervision of Experiments on Animals (CPCSEA). Each monkey was surgically implanted with a titanium head post and trained on a visual fixation task after recovery. After satisfactory training, a 96-channel microarray (10 × 10 Utah array; 1 mm long, 400 μm interelectrode distance; Blackrock Microsystems) was implanted in the V1 of right cerebral hemisphere (∼15 mm rostral to the occipital ridge; ∼15 mm lateral to the midline). Further details have been presented in previous studies (Murty et al., 2018). These recordings were used only for parametric chromatic stimuli (see below for details) and are labeled M1R and M2R for the two monkeys to distinguish from the main dataset recorded using the hybrid array as described below.

After these arrays were inactive, another surgery was carried out under general anesthesia, and a customized hybrid array was implanted in the left hemisphere of both animals. The array had 81 microelectrodes (9 × 9; 1 mm long, 400 μm interelectrode distance, 3–5 μm tip diameter; Blackrock Microsystems) and 9 ECoG electrodes (3 × 3; 10 mm interelectrode distance, 2.3 mm diameter; Ad-Tech Medical Instrument Corporation), both connected to a single Blackrock 96-channel connector. A craniotomy and a durotomy were performed and the ECoG strip slid under the dura [Dubey and Ray (2019), their Fig. 1]. A hole made in the silastic was aligned with the durotomy, where the microarray was then inserted (10–15 mm rostral to the occipital ridge; 10–15 mm lateral to the midline). For Monkey 2, the ECoG strip did not smoothly slide along the cortex, and one column (three electrodes) had to be removed, resulting in four ECoGs on V1. Two reference wires were either inserted by the edge of the craniotomy or wound over the titanium screws holding the metal strap in place to secure the bone on the craniotomy.

After adequate recovery, the monkeys performed experimental tasks. Signals were recorded using Cerebus Neural Signal Processor (Blackrock). LFP and ECoG signals were obtained by bandpass filtering the raw data between 0.3 Hz (analog Butterworth filter, first order) and 500 Hz (digital Butterworth filter, fourth order) and sampling at 2,000 Hz. The head fixed monkey viewed a monitor screen (BenQ XL 2411, LCD, 1,280 × 720, refresh rate 100 Hz; gamma corrected) at ∼50 cm distance. A trial began with a fixation dot (0.05 or 0.10° radius) appearing on the gray screen at the center. The monkey passively fixated on the dot and after 1,000 ms, 2–4 stimuli successively appeared for 800 ms each with gray interstimulus screen for 700 ms, unless otherwise stated. The dot remained on during the whole trial and maintaining fixation within 2° around it resulted in a juice reward; otherwise the trial was aborted.

### Stimuli

Three categories of stimuli have been used in this study: parametric achromatic gratings, parametric chromatic patches, and natural images. The responses to chromatic patches used to derive the model were recorded from the right hemispheres using standard Utah arrays. Responses to the gratings and images were recorded from the left cerebral hemisphere of the monkeys using hybrid (microelectrode + ECoG) arrays. Additionally, responses for a subset of chromatic hues were recorded from the left hemisphere to compare the tuning across both hemispheres, but this data was not used in constructing the model.

#### Achromatic gratings

We studied four features for static achromatic gratings—orientation, spatial frequency (SF), size, and contrast. Unfortunately, the number of all possible combinations of these features is prohibitively large. Therefore, we varied only two features per recording session, with orientation being covaried with one other feature, leading to three combinations—SF-Ori, Size-Ori, and Con-Ori. In the SF-Ori protocol, a static full-screen, full-contrast grating at one of five spatial frequencies [0.5, 1, 2, 4, and 8 cycles per degree (cpd)] and one of eight orientations (0, 22.5, 45, 67.5, 90, 112.5, 135, and 157.5°) was displayed pseudorandomly, while the monkey fixated at the center of the screen. During the Size-Ori protocols, a static full-contrast grating was presented centered at the RF of one of the recording sites (different site per session). The orientation and size were pseudorandomly chosen from among eight orientations (0, 22.5, 45, 67.5, 90, 112.5, 135, and 157.5°) and six sizes (0.3, 0.6, 1.2, 2.4, 4.8, and 9.6°). For the Con-Ori protocol, full-screen gratings were presented at 4 cpd (M1) or 2 cpd (M2). The stimulus could take one of seven contrasts (0, 3.12, 6.25, 12.5, 25, 50, and 100%) and four orientations (0, 45, 90, and 135°) for M1 and one of six contrasts (0, 6.25, 12.5, 25, 50, and 100%) and eight orientations (0, 22.5, 45, 67.5, 90, 112.5, 135, and 157.5°) for M2. Details of the stimuli are summarized in Table 1 and have also been presented previously (Dubey and Ray, 2020).

**Table 1.** Stimulus details and separability indices

Protocol name	Monkey, no. of electrodes	Size (dva)	Contrast or value	SF or saturation	Orientation or hue (°)	Mean no. of trials ± SD	Mean separability index, *s_i_* ± SD, significant electrodes
Spatial frequency-orientation (SF-Ori)	M1, 82	Full screen	100%	0.5, 1, 2, 4, 8 cpd	0, 22.5, 45, 67.5, 90, 112.5, 135, 157.5	34.6 ± 1	0.98 ± 0.01, 80
	M2, 21					47 ± 1.8	0.98 ± 0.01, 21
Size orientation (Size-Ori)	M1, 29	0.3, 0.6, 1.2, 2.4, 4.8, 9.6	100%	4 cpd	0, 22.5, 45, 67.5, 90, 112.5, 135, 157.5	12 ± 5.9	0.97 ± 0.02, 29
	M2, 22					12.6 ± 4.1	0.97 ± 0.01, 18
Contrast orientation (Con-Ori)	M1, 82	Full screen	0, 3.12, 6.25, 12.5, 25, 50, 100%	4 cpd	0, 45, 90, 135	9.9 ± 0.4	0.95 ± 0.02, 81
	M2, 21		0, 6.25, 12.5, 25, 50, 100%	2 cpd	0, 22.5, 45, 67.5, 90, 112.5, 135, 157.5	27.9 ± 1.6	0.99 ± 0.01, 21
Hue	M1R, 65	Full screen	1	1	0, 10, 20, … 340, 350 (36 values).	20.05 ± 6.2	
	M2R, 39					30.03 ± 0.74	
Hue saturation (Hue-Sat)	M1R, 65	Full screen	1	0, 0.25, 0.5, 0.75, 1	0, 60, 120, 180, 240, 300	12.4 ± 0.7	0.94 ± 0.02, 52
	M2R, 39					14.1 ± 1.4	0.95 ± 0.01, 39
Hue value (Hue-Val)	M1R, 65	Full screen	0, 0.25, 0.5, 0.75, 1	1	0, 60, 120, 180, 240, 300	11.8 ± 0.6	0.97 ± 0.02, 62
	M2R, 39					27.2 ± 0.6	0.99 ± 0.00, 39
Saturation value (Sat-Val)	M1R, 19	9.6	0, 0.25, 0.5, 0.75, 1	0, 0.25, 0.5, 0.75, 1	0	11.92 ± 4.6	0.98 ± 0.01, 19
Hue size	M1R, 11	0.15, 0.3, 0.6, 1.2, 2.4, 4.8, 9.6	1	1	0 (red) (M1R also had cyan hue displayed but data not used for uniformity)	12.4 ± 5.2	
	M2R, 35	0.5, 0.68, 0.92, 1.26, 1.72, 2.34, 3.2				15.9 ± 0.4	
Hue-6	M1, 82	Full screen	1	1	0, 60, 120, 180, 240, 300	29 ± 1	
	M2, 21					32.6 ± 1.4	

List of parametric protocols with the number of electrodes and trials, and stimulus details. Sizes are in degree visual angle (dva), contrasts in percentage, spatial frequencies in cycles per degree (cpd), and orientations in degrees. Value and saturation are dimensionless and hues are in degrees. The last column reports the average separability index (*si*; see Results) for protocols with two stimulus features. The number of electrodes with the *si* significantly different from a random distribution (*t* test; *p* ≤ 0.05) is mentioned.

#### Chromatic stimuli

We used data from previous recordings from the same monkeys from a different (right) hemisphere, since gamma tuning preferences do not vary appreciably with location (Dubey and Ray, 2020) and chromatic space was mapped more thoroughly in these older recordings (Shirhatti and Ray, 2018). Nonetheless, we verified that the tuning preference for hue was consistent across the two hemispheres for these monkeys (see below).

We used data in response to features of chromatic stimuli in the HSV space (hue, saturation, and value) along with size, across five protocols. In the hue protocol, full-screen hues were presented at full saturation and value. The hues were pseudorandomly chosen from 36 values equi-spaced over the hue spectrum with 0° representing red and 350° representing magenta. In the Hue-Size protocols, a chromatic patch at maximum saturation and value was centered at the RF of one of the recording sites in each session. The size was chosen from among seven values (Table 1), and the hue was fixed at red. For M1, cyan hue was also presented but not used here for uniformity. The Hue-Saturation protocol used full-screen stimuli at six equi-spaced hues (0, 60, 120, 180, 240, and 300°) and five saturation levels (0, 0.2, 0.4, 0.6, 0.8, and 1) while the value was 1. In Hue-Value protocols, the saturation was fixed at 1, while the six equi-spaced hues were presented at one of 5 values (0, 0.2, 0.4, 0.6, 0.8, and 1). Lastly, in the Saturation-Value protocol, the hue (red) and size (9.6 dva) were fixed, while the saturation and value could each take one of 5 values (0, 0.2, 0.4, 0.6, 0.8, and 1). These stimuli have also been described previously (Shirhatti and Ray, 2018) and are summarized in Table 1.

Using the hybrid arrays in the left hemisphere, we recorded responses to a contracted hue protocol (Hue-6), in which six full-screen hues (0, 60, 120, 180, 240, and 300°) were displayed at full saturation and full value. The stimuli were randomly interleaved and appeared for 500 ms with interstimulus period of 500 ms.

#### Image stimuli

Naturalistic stimuli used have been described earlier (Kanth and Ray, 2020). Briefly, 64 images from the McGill Color Calibrated Image Database (Olmos and Kingdom, 2004) (http://tabby.vision.mcgill.ca/html/browsedownload.html) were grouped in four categories of 16 images each—Fauna, Flora, Textures, and Landscapes. We added a set of 16 face images. All were cropped and downsampled to 1,280 × 720 pixels. One set of 16 images is shown in Figure 5 and all images are shown in Figure 4*C* of Kanth and Ray (2020). During a session, either 16 (from 1 category) or 32 (from 2 categories) randomly interleaved full-screen stimuli were displayed. We also made their grayscale versions which were displayed in separate sessions. In each trial, 2–4 images were displayed for 500 ms each with an interstimulus interval of 500 ms. The average number of trials per stimulus was 72.25 (±15.57 SD) for M1 and 69.9 (±5.66 SD) for M2.

Post recordings, the images were displayed again and the screenshots (1,280 × 720 pixels) of the display screen were taken. These screenshots in the HSV format [three layers for hue, saturation, and luminance (value)] were used to obtain image patches around the RF centers of electrodes. For grayscale images, only the luminance layer is relevant, with the hue and saturation set to 0.

### Electrode selection

Electrodes were chosen using a RF mapping protocol described previously (Dubey and Ray, 2019). Small gratings were flashed across the visual field and electrodes with consistent responses, and reliable RF estimates across sessions were selected. We chose ECoGs which were posterior to the lunate sulcus and had a minimum response value above 100 μV. We obtained 77 microelectrodes (+5 ECoGs) for M1, 17 microelectrodes (+4 ECoGs) for M2, 65 microelectrodes for M1R, and 39 microelectrodes for M2R.

The Size-Ori stimuli were displayed at RFs of certain electrodes. In each session we selected electrodes within 0.2° of the stimulus center, further choosing those with average firing rate ≥1 spike/s (250–750 ms) and a signal-to-noise ratio >1.5 (details in Dubey and Ray 2020). Some electrodes were chosen on multiple sessions, yielding 24 and 18 nonunique microelectrodes (and five and four ECoGs) for M1 and M2, respectively. In the Hue-Size protocols, we selected electrodes within 0.3° of the stimulus center—11 in M1R and 35 in M2R. For the Sat-Value protocol of M1R, 19 electrodes that were within 0.5° of the stimulus center were chosen.

### Power calculation

Power spectral density (PSD) was calculated by the Multitaper method ([ Slepian taper; Chronux toolbox (Bokil et al., 2010): https://chronux.org/]. The baseline period was 250–0 ms before and stimulus period was 250–500 ms after stimulus onset to avoid onset-related transients. Power was calculated separately for each trial and then averaged to get the PSD for each electrode. For the change in power, stimulus PSD was normalized by the baseline PSD for each electrode. To get the gamma response, PSD values within the 30–80 Hz band were summed and then normalized by the summed baseline power in the same range.

### Separability analysis

We assessed the separability of two covarying stimulus parameters using the trial-averaged change in gamma band power as the response. We obtained a two-dimensional tuning matrix where one stimulus feature varied across columns and the other across rows. This matrix was expressed using a multiplicative model in two ways—through singular value decomposition (SVD) and through marginals (Peña and Konishi, 2001; Mazer et al., 2002). SVD allows to decompose a matrix, *M* as follows:

$$M=U*\lambda *V^{T}.$$

Here, *U* and *V* contain orthonormal vectors weighted by singular values ordered in the diagonal matrix *λ*. The magnitude of the singular value represents how well *M* can be approximated using the corresponding singular vectors. Since the first singular value (*λ*_1_) is the largest, the first singular vectors (*U*1 and *V*1) represent the best independent separable factors of *M*. For a perfectly separable matrix, the other singular values are zero. To quantify the separability, we calculated a separability index (Mazer et al., 2002) based on the contribution of the first singular vector to the overall representation:

$$si=\;\frac{\lambda_{1}^{2}}{\sum_{i}\;\lambda_{i}^{2}}.$$

The first singular vectors represented the data using the linear model:

$$M\;∼\;1+\;U_{1}*\lambda_{1}*V_{1}^{T}.$$

Here, the first term is an intercept, and the second term is the product of the first singular vectors weighted by the first singular value. We used linear regression (Matlab function *fitlm*) to fit this model to the data and obtain coefficients for each term. To evaluate the goodness of fit, we calculated the coefficient of determination (*R*^2^), which represents the percentage of data variance explained by the model and is equivalent to squared Pearson’s correlation for linear regression (Everitt and Skrondal, 2010; Slinker et al., 2016).

In the second approach, we obtained the marginals, *F* and *G*, from the data matrix by summing across columns and rows, respectively. These represent the tuning to one feature independent of the other. In the case of truly independent features, the matrix can be reconstructed from the product of marginals as follows:

M∼1+F*G.

Here, the first term represents an intercept. We used linear regression to fit this model to the data and obtain the *R*^2^ metric. For comparison, we tested another linearly separable model (as in Peña and Konishi 2001) based on the addition of the marginals:

M∼F+G.

Using linear regression, we obtained the coefficients for both terms and the *R*^2^ metric. There is no intercept to keep the degrees of freedom same as in Equations 3 and 4.

### Statistical tests

To test the significance of the calculated separability index *si*, we made a similarly sized two-dimensional matrix with samples randomly chosen from a normal distribution (same mean and variance as the data matrix) and calculated the *si* of that matrix. We performed 20 such iterations and tested if the *si* of the data was significantly different from the bootstrapped values (Student's *t* test; alpha = 0.05). We compared the *R*^2^ of the marginal product model with the marginal sum model and the SVM product model. We used the values for all electrodes from two models and performed an unpaired *t* test. Significance was tested at alpha = 0.05 (*). Differences with alpha <0.005 (**) are also denoted in Figure 2.

### Modeling of grating tuning functions

We used the SF-Ori, Size-Ori, and Con-Ori protocols to obtain tuning functions of single features and then modeled the joint responses as product of individual functions. Trials were divided into two nonoverlapping folds; average of one fold was used to learn the feature tuning and the other to test it. Three iterations were performed in which different trials were allotted to each fold. The correlation values were subsequently averaged over iterations.

Spatial frequency dependence was modeled as a Gaussian function over the spatial frequency. Since SF values were chosen nonlinearly (0.5, 1, 2, 4, and 8 cpd), we linearized them by a log_2_ transformation. Orientation tuning was modeled as a von Mises function over orientation in radians. Since the orientation varied from 0 to 157°, the orientation values were multiplied by 2 to encompass the domain of the von Mises function. These functions are defined as follows:

$$F_{\mathrm{SF}}\left(f\right)=\;A*N_{\left(\mu ,\sigma \right)}\left(\mathrm{lo}g_{2}\left(f\right)\right)+B,$$

$$F_{O}\left(\theta \right)=\;A*VM_{\left(\alpha ,\kappa \right)}\left(2\theta \right)+B.$$

Here *f* and *θ* represent the spatial frequency and the orientation, respectively. *N*_(*μ*,*σ*)_ and VM_(*α*,*κ*)_ represent the Gaussian and von Mises probability density functions, respectively, normalized by their maximum response, to attain a peak value of 1. Parameters for these functions were derived from the two-dimensional SF-Ori data. Employing least squares curve fitting, we estimated *μ* and *σ* (center and sigma of Gaussian), and *α* and *κ* (center and spread of von Mises) as well as *A* (gain) and *B* (offset) for each electrode. Each equation has 4 free parameters, fitted using 40 data points (5 SF × 8 orientation). *A* and *B* do not affect the shape of the curve but merely adjust the magnitude. The response was presumed to be maximum at the full-screen size and at full contrast and scaled down for lower values. The size and contrast values were log transformed to a linear scale from a geometric sequence. Both size and contrast responses were modeled as sigmoids, characterized by a slope (*m* for size, *k* for contrast) and a midpoint (*σ*_0_ for size, *c*_0_ for contrast) determining the shape of the curve:

$$F_{\mathrm{SZ}}\left(r\right)=\;\frac{A}{1+10^{-m\left(\mathrm{lo}g_{2}\left(r\right)-\mathrm{lo}g_{2}\left(\sigma_{0}\right)\right)}\;}+B,$$

$$F_{\mathrm{CN}}\left(c\right)=\;\frac{A}{1+10^{-k\left(\mathrm{lo}g_{2}\left(c\right)-\mathrm{lo}g_{2}\left(c_{0}\right)\right)}}+B.$$

Along with the gain (*A*) and offset (*B*), four parameters were fitted for each equation, using two-dimensional data pooled across orientations. Equation 8 was fitted using 48 data points (6 sizes × 8 orientations), while Equation 9 utilized 28 data points (M1, 7 contrasts × 4 orientations) and 48 data points (M2, 6 contrasts × 8 orientations).

Across the three protocols, we obtained eight free parameters—*μ*, *σ*, *α*, *κ*, *m*, *σ*_0_, *k*, and *c*_0_ determining the tuning curve shape for each electrode. The modeled response to a stimulus was a product of responses to individual features using these parameters.

$$F\left(\;f,\theta ,r,c\right)=\;\frac{N_{\left(\mu ,\sigma \right)}\left(\mathrm{lo}g_{2}\left(f\right)\right)*VM_{\left(\alpha ,\kappa \right)}\left(2\theta \right)}{\left(1+10^{-m\left(\mathrm{lo}g_{2}\left(r\right)-\mathrm{lo}g_{2}\left(\sigma_{0}\right)\right)}\right)*\left(1+10^{-k\left(\mathrm{lo}g_{2}\left(c\right)-\mathrm{lo}g_{2}\left(c_{0}\right)\right)}\right)}\;,$$

$$F_{m}\left(\;f,\theta ,r,c\right)=G*F\left(\;f,\theta ,\sigma ,c\right)+O.$$

Equation 10A depends only on stimulus features and tuning parameters. To get the scaled estimates (shown in Fig. 3*A*), data from the other fold along with the output of Equation 10A was used to fit the gain (*G*) and intercept (*O*; Eq. 10B). Linear correlation of this estimate with the actual data was then computed.

### Modeling hue tuning functions

We used the Hue, Hue-Size, Hue-Saturation, and Hue-Value protocols recorded from M1R or M2R to arrive at hue tuning functions. The data was separated into two cross-validated folds over three iterations, as done for the grating functions. Hue dependence was modeled as a sum of two von Mises functions spread over the circular hue space, with centers initially placed at red (0°) and cyan (180°) hues. The response to a full-screen hue (*H*) was the following:

$$F_{H}\left(H\right)=A*\left(VM_{\left(\alpha r,\kappa r\right)}\left(H\right)+a_{c}VM_{\left(\alpha c,\kappa c\right)}\left(H\right)\right)+B.$$

The fitting function could move the centers by up to 80° on either side of red and 100° on either side of cyan. The gains of the functions were normalized to that at red to be 1 and *a_c_*. The centers (*αr*, *αc*), spreads (*κr*, *κc*), and gain (*a_c_*) of the von Mises functions and the coefficients (*A*, *B*) were fit from the Hue data (36 hues) supplemented by the maximum saturation and value responses of six hues from the Hue-Sat and Hue-Val stimuli, providing 48 data points to fit 7 free parameters.

Responses to full screen and full saturation were considered maximum and were scaled down for lower values. The size dependence was modeled as a sigmoid over linearized size values. It had four parameters (slope *m*, midpoint *σ*_0_, gain *A*, offset *B*) fit using the size data of red hue (7 data points):

$$F_{\mathrm{SZ}}\left(R\right)=\;\frac{A}{1+10^{-m\left(\mathrm{lo}g_{2}\left(R\right)-\mathrm{lo}g_{2}\left(\sigma_{0}\right)\right)}}+B.$$

Saturation tuning was modeled as sigmoid with slope (*l*) and midpoint (*s*_0_) along with the gain (*A*) and offset (*B*). These parameters were fit using 30 data points (6 hues × 5 saturations) of Hue-Sat dataset:

$$F_{S}\left(S\right)=\;\frac{A}{1+10^{-l\left(S-s_{0}\right)}}+B.$$

In case of Hue-Value, the gamma did not vary for values 25% and upward. The model only considered values from 25%, as we did not have data between 0 and 25%. The values were scaled between 0 and 1 from a percentage, and the dependence was modeled as a linear function, with 24 (4 values × 6 hues) data points to fit the gain and intercept:

$$F_{V}\left(V\right)=\;g*\frac{V}{100}+o.$$

The overall responses were then estimated as a product of individual feature responses:

$$F\left(H,S,V,R\right)=\;\frac{\left(VM_{\left(\alpha r,\kappa r\right)}\left(H\right)+a_{c}VM_{\left(\alpha c,\kappa c\right)}\left(H\right)\right)*\left(g\frac{V}{100}+o\right)}{\left(1+10^{-m\left(\mathrm{lo}g_{2}\left(R\right)-\mathrm{lo}g_{2}\left(\sigma_{0}\right)\right)}\right)*\left(1+10^{-l\left(S-s_{o}\right)}\right)\;}\;,$$

$$F_{m}\left(H,S,V,R\right)=G*F\left(H,S,V,R\right)+O.$$

We used the 11 parameters (*αr*, *αc*, *κr*, *κc*, *a_c_*, *m*, *σ*_0_, *l*, *s*_0_, *g_v_*, and *o_v_*) obtained from the individual functions to arrive at the modeled response (Eq. 15A). The gain *G* and intercept *O* were estimated by fitting this response to the other fold to scale the magnitude (Eq. 15B). The same model (Eq. 15A) was used to predict responses to images. The only difference was that parameters were obtained using all trials and not from two separate folds. This did not change the cross-validated nature since the image data had no part in calculating the parameters.

### Image patch statistics

We analyzed the image around the RF of each electrode for grating-like luminance changes or unvarying chromatic features. Most images could be approximated by either a Gabor or a uniform hue patch, which could be defined by simple features.

#### Hue patch approximation

For each image at each electrode's RF, we got four parameters—*H*, *S*, *V*, and *R*—which could be used with Equation 15A to estimate a response. The images were in HSV format, and we first chose pixels within 0.3° radius around the RF center. We then calculated the mean (*mS*_1_, *mV*_1_) and the standard deviation (*sS*_1_, *sV*_1_) of saturation and value of pixels contained in this patch. We also scaled the hues of chosen pixels from 0 to 360° to 0–2*π* and calculated the circular mean (*mH*_1_) and circular standard deviation (*sH*_1_).

For subsequent larger sizes (increments of 0.3° until 2.1°), we calculated the same quantities (*mH_n_*, *mS_n_*, *mV_n_*, *sH_n_*, *sS_n_*, *sV_n_*) using pixels in the incremental ring, excluding those from the previous size. To determine the uniformity of the chromatic patch, we then applied thresholds on the standard deviations and the difference of the means from the first size (*mH_n_*–*mH*_1_, *mS_n_*–*mS*_1_, *mV_n_*–*mV*_1_). This ensured that the HSV values did not change substantially as the radius around the RF center was increased. If the standard deviations for hue, saturation, and value exceeded 0.1, 0.2, and 0.2, respectively, or their respective mean differences crossed 0.05, 0.1, and 0.1, and remained high for the subsequent two radii, we identified that radius as superscribing a uniform HSV patch. These thresholds were chosen because the estimated patch sizes appeared reasonable upon visual inspection, although none of the results shown here critically depend on the threshold (see Results). If the values dropped below the threshold within two sizes of exceeding it, that radius was skipped and the next threshold crossing was chosen. Patches for which the threshold was crossed at the first size itself were assigned a size of 0 to indicate that a uniform HSV patch was not identified. For the pixels included within the patch of radius *R*, their average value (*V*) was calculated. Pixel-wise angular hues (*θ*) were weighted by the corresponding saturations (*s*) and treated as vectors to get the average hue (*H*) and saturation (*S*):

$$H=\mathrm{ang}\left(\sum s*e^{i\theta }\right),$$

$$S=\mathrm{mag}\left(\sum s*e^{i\theta }\right).$$

#### Gabor patch approximation

Using the V layer (of HSV image), we cropped a 14° wide square around the RF center of each electrode. A Gaussian mask (sigma = 0.3°) was used to highlight the image. Pixels farther than 3 times sigma were set to 0. We removed the DC component from the masked image and employed a two-dimensional Fourier transform to get the magnitude spectrum in the SF and orientation domain. Since natural images have higher power at low frequencies (1/f spectrum), we skipped the first 0.1 cpd and selected the maximum peak beyond that to represent the SF and orientation of the image at the chosen size. This was repeated for increasing sizes of the Gaussian mask (increments of 0.3° until 2.1°). To compare across sizes, we normalized these selected magnitudes with the Euclidean norm of the corresponding Gabor (matched in size, SF, and orientation). We also obtained the orientation variance (OV; Hermes et al., 2019) at each size and SF by calculating the variance of magnitudes across orientations. To represent the image patch by a Gabor, we selected the size with the highest normalized magnitude and in case of a tie, the one with the higher OV. Thus, we got the SF (*f*), orientation (*θ*), and size (*r*) of the Gabor to match the image. We also estimated the phase (*p*) and the Michelson contrast (*c*) of the image patch.

We finally selected patches which had SFs between 0.4 and 8 cpd and OV > 20. These limits were chosen because lower SFs could be represented by a small uniform patch. Further, low OVs implied that the chosen orientation was not dominant, and other orientations could be present, preventing the patch from being well approximated by a Gabor. As discussed in the Results, very few image patches qualified as Gabors. This was also observed upon visual inspection—only a small minority had regularly alternating spatial luminance values which would yield a large peak in the 2-D spatial Fourier transform (Fig. 5*A*, image 4); most images instead were better represented by uniform hue patches.

### Code availability

These computational codes for this study were written in Matlab and can be run on a computer operating on macOS (10.13.6 or later) or Microsoft Windows with Matlab 2022b (or later) installed. These codes can be accessed in the Extended Data and can also be found in the following GitHub repository: https://github.com/SidratTK/NatImagesGammaRepo.

10.1523/ENEURO.0417-23.2024.d1 Extended Data Download Extended Data, ZIP file.

## Results

### Gamma responses to a pair of parametric features are separable

We first tested a pair of stimulus features for separable gamma tuning. If separable, joint responses can be reconstructed from individual feature dependent functions. We followed two methods, SVD (Peña and Konishi, 2001) and marginals (Mazer et al., 2002), to analyze the joint tuning as a product of independent factors. SVD factorizes a matrix into independent vectors, whose weighted sum of products can reconstruct the matrix ( Eq.1). For perfect separability, the matrix can be reconstructed as a product of the first left and right singular vectors, weighted by the first singular value. We quantified this measure of independence by a separability index (*s_i_*) which is 1 for a perfectly separable case (Mazer et al., 2002 and Eq. 2). SVD provides independent factors representing some combination of the one-dimensional tuning which may not always be the same as the marginals, which are obtained by simply averaging across rows or columns to get the dependence on one feature irrespective of the second feature. Marginals are an explicit and easy way to obtain tuning of the independent features.

For achromatic gratings, we considered Ori, SF, contrast (Con), and size as the features. Since the number of stimuli required to investigate joint tuning along all features was prohibitively large, we fixed one feature (Ori) and investigated its joint tuning with other features (SF, Con, and Size).

#### SF-Ori

**Figure 1.**
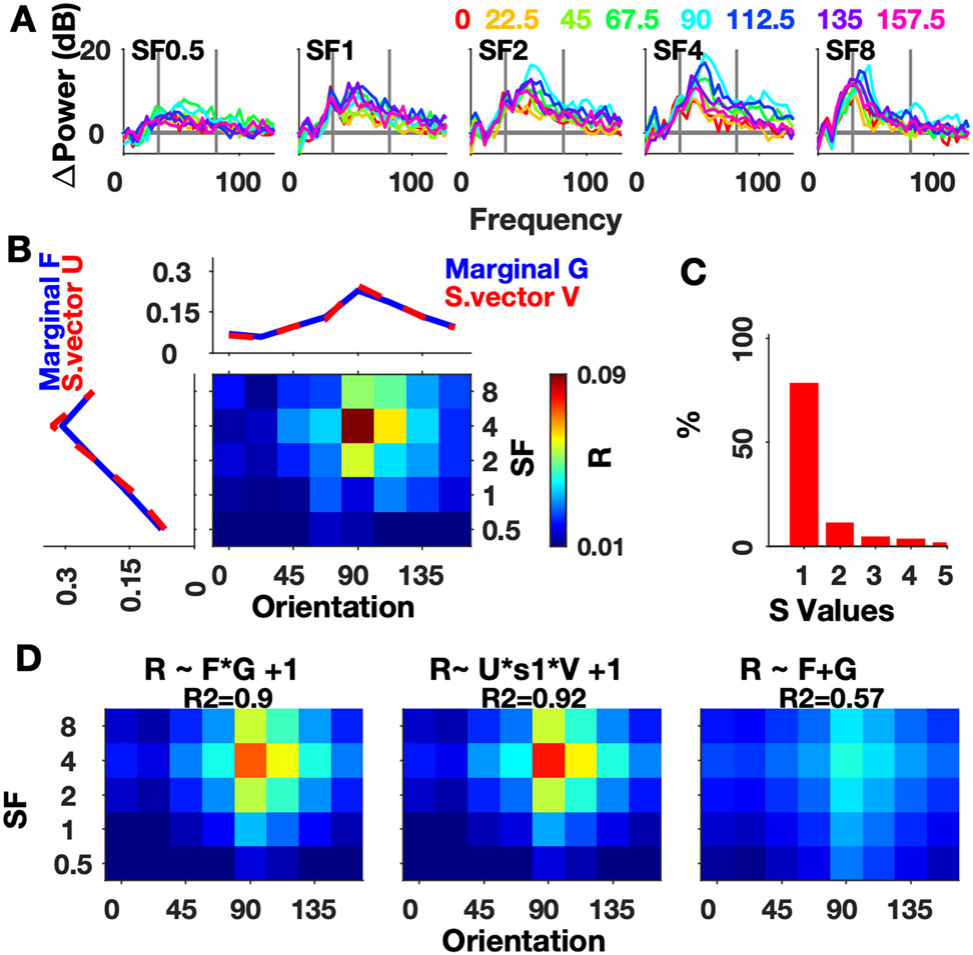
Separability of orientation and spatial frequency tuning for one electrode (M1, e7). ***A***, Frequency spectra of the log change in power from baseline (−250 to 0 ms) in 250–500 ms period for five spatial frequencies (SF) and eight orientations (Ori) for the SF-Ori protocol. Colors correspond to orientations (indicated above the figure). Gray vertical lines mark the gamma band (30–80 Hz). ***B***, Trial-averaged gamma responses to stimuli varying in Ori (columns) and SF (rows) for this electrode. Marginals are shown in blue, and the red curves show the first singular vectors from the SVD of the 2-D matrix. Responses, marginals, and singular vectors are scaled so that area under the curve equals 1. ***C***, Percentage contribution of ordered singular values obtained from SVD. ***D***, Modeled separable joint matrix as a function of independent vectors with the corresponding ratio of variance explained (*R*^2^) by product of marginals (left), by product of singular vectors (middle), and by sum of marginals (right). The gamma responses to grating features are summarized in Extended Data Figure 1-1 and to chromatic features in Extended Data Figure 1-2.

Full-screen, maximum contrast gratings varying in Ori (8 values) and SF (5 values) were displayed in the “SF-Ori” protocol (see Materials and Methods). Figure 1*A* shows the change in power from baseline to SF-Ori stimuli for a typical electrode from M1. These were obtained by estimating the power between 250 and 500 ms after stimulus onset and normalizing by the baseline power (−250–0 ms; see Materials and Methods). These stimuli evoked strong gamma rhythms as can be observed by the spectral peaks especially for SF of 2 and 4 cycles per degree (cpd) stimuli, particularly at 90° orientation (cyan traces). These large stimuli elicited two peaks, which have been termed slow and fast gamma (Murty et al., 2018). As has been previously reported using the same monkeys (Dubey and Ray, 2020), gamma peak frequency varied across SF-Ori conditions (as well as size and contrast manipulations, see Dubey and Ray, 2020). As shown later, natural images often did not induce a strong gamma rhythm, and for most, a clear peak was not present in the spectrum. Therefore, we considered a fixed frequency range of 30–80 Hz (Fig. 1*A*, gray vertical lines) and used the change in power averaged in this range as our “gamma response.” This is shown as a two-dimensional matrix over SFs and Oris in Figure 1*B* (scaled to have an area of 1 to be represented as a joint density function), over which we performed SVD and marginal analysis. The change in gamma power averaged over all electrodes is shown in Extended Data Figure 1-1.

10.1523/ENEURO.0417-23.2024.f1-1 Figure 1-1 **Gamma response to grating features.** Response refers to the change in gamma band (30-80  Hz) power from baseline (-250-0  ms) in 250-500  ms period, expressed on a log scale in decibels. The spatial frequency (SF) tuning is obtained by averaging over 8 orientations in the SF-Ori data. The orientation tuning uses the same data averaged across 5 SFs. The size response is displayed using Size-Ori data and averaging across 8 orientations, while the contrast response utilizes the Con-Ori data averaged over orientations. The responses are averaged over electrodes (magenta: LFP, blue: ECoG), and error bars denote SEM. Download Figure 1-1, TIF file.

**Figure 2.**
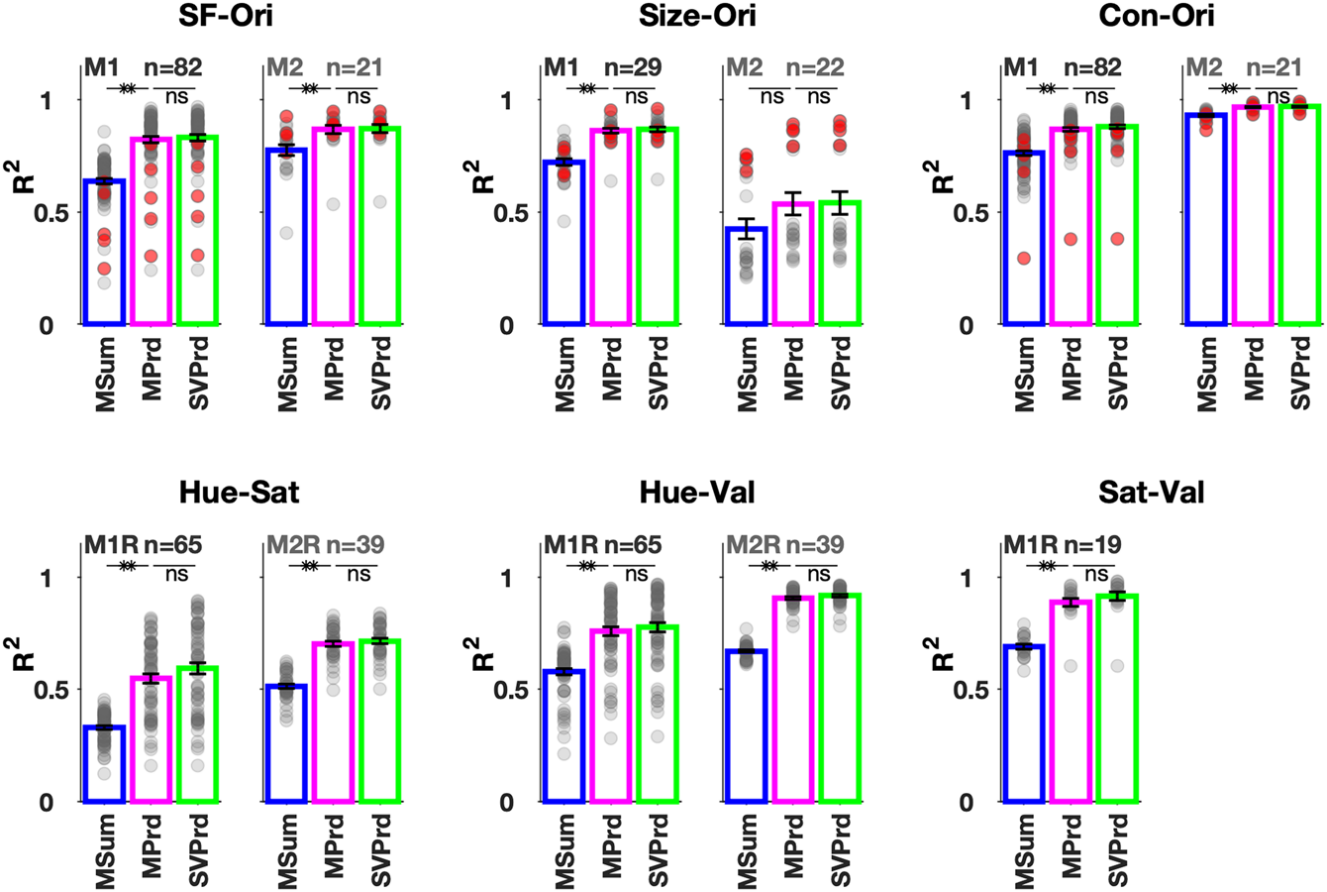
Population level separability. The ratio of variance explained (*R*^2^) of all protocols. M1R and M2R represent the right hemispheres, while M1 and M2 are the left hemispheres of the same two monkeys. The gray and red dots represent the individual microelectrodes and ECoGs, respectively. The electrode averages are plotted as bars for the three approaches used—sum of marginals (MSum, blue), product of marginals (MPrd, magenta), and product of singular vectors (SVPrd, green; **p ≤ 0.005, unpaired *t* test).

10.1523/ENEURO.0417-23.2024.f1-2 Figure 1-2 **Gamma response to chromatic features.** Response refers to the change in gamma band (30-80  Hz) power from baseline (-250-0  ms) in 250-500  ms period, expressed on a log scale in decibels. The hue tuning is obtained from the Hue data in which 36 hues were displayed (with 0° signifying red) for M1R and M2R. The dotted lines are for M1 and M2 (same monkeys, other cerebral hemisphere) showing LFP (red) and ECoG (blue) responses to 6 hues. The saturation (Sat) tuning uses the Hue-Sat data averaged across 6 hues, while the value (Val) response utilizes the Hue-Val data averaged over the 6 hues. The Size response is displayed using Hue-Size data for the red hue. All responses are averaged over electrodes (magenta, red: LFP, blue: ECoG), and error bars denote SEM. Download Figure 1-2, TIF file.

The first singular value represents the contribution of the first singular vectors from the SVD (Fig. 1*B*, red curves) to the joint distribution. The relative contribution of the singular values (Fig. 1*C*) reveals that the first one had a much higher contribution compared with the others, and consequently the joint distribution had a high separability index (*s_i _*= 0.97). Based on the product of the first singular vectors, we modeled a joint response matrix (Fig. 1*D*, middle; see Materials and Methods). We obtained the coefficient of determination (*R*^2^ = 0.92) which quantifies how much of the data variance can be explained by the model. In the second approach, we modeled the joint response as a product of the marginal Ori and SF tunings which are plotted in Figure 1*B* (blue curves) and were highly overlapping with the first singular vectors. The modeled two-dimensional response using the product of marginals (Fig. 1*D*, left) also had a high *R*^2^ (0.9). For comparison, we also used an additive model (see Materials and Methods) which is another separable marginal model. It explained much less variance (Fig. 1*D*, right; *R*^2^ = 0.57) compared with the product models.

This behavior was mirrored across microelectrodes and ECoGs. The average separability index was 0.98 ± 0.01 SD (*n* = 82) for M1 and 0.98 ± 0.01 SD (*n* = 21) for M2. We tested *si* against bootstrapped values (see Materials and Methods) and found it to be significantly different (*t* test; *p* ≤ 0.05) from separability indices of randomly constructed matrices for 80 (of 82) electrodes of M1 and all electrodes of M2. Average *R*^2^ for the SVD-based model (Fig. 2, “SF-Ori” plot; green bars) was 0.83 ± 0.13 SD (M1) and 0.87 ± 0.08 SD (M2), similar to values obtained by the marginals product model (magenta bars): 0.82 ± 0.13 SD (M1) and 0.87 ± 0.08 SD (M2). The additive model (blue bars) explained significantly lesser variance: 0.64 ± 0.11 SD (M1) and 0.78 ± 0.11 SD (M2).

#### Size-Ori and Con-Ori

Responses to varying size and orientation (Size-Ori) and varying contrast and orientation (Con-Ori) were recorded separately. In the Size-Ori protocol, full-contrast gratings at eight orientations and six sizes were displayed centered on specific RFs (see Materials and Methods). The Con-Ori protocol used full-screen gratings at 6 (or 7) contrasts and 8 (or 4) orientations (see Materials and Methods). The gamma response to size and contrast is summarized in Extended Data Figure 1-1, averaged over electrodes. As before, we obtained the two-dimensional gamma responses (power change in 30–80 Hz) for these protocols for all available electrodes, derived the marginals and singular vectors from them, and made marginal product, marginal sum, and singular vector product-based models. Their *R*^2^ values and other metrics are summarized in Figure 2 and Table 1.

**Figure 3.**
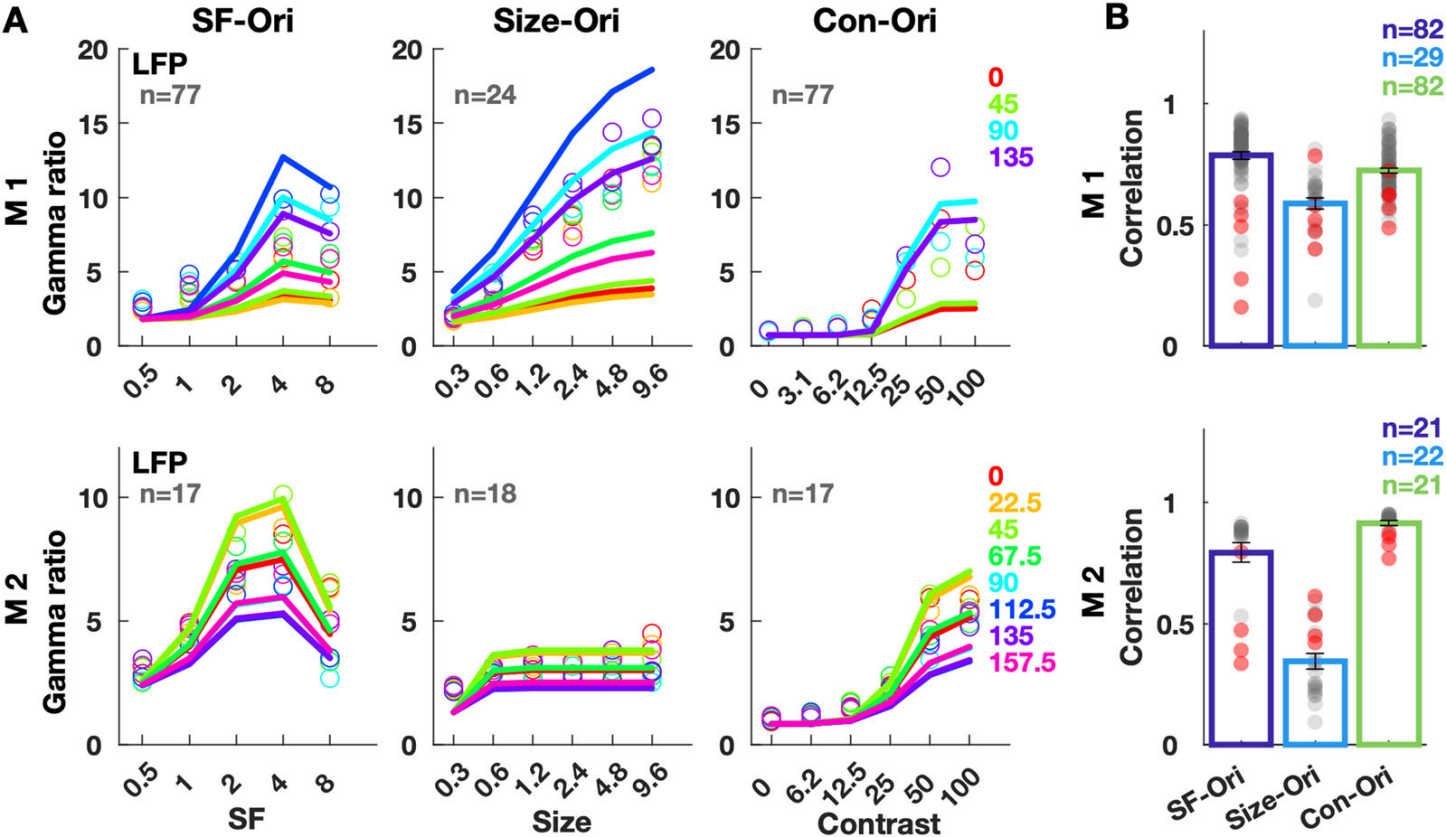
Gamma response to parametric gratings and estimates. Response refers to the change in gamma band (30–80 Hz) power from baseline (−250 to 0 ms) in 250–500 ms period. ***A***, Data (open circles) and estimated responses to the SF-Ori, Size-Ori, and Con-Ori stimuli, averaged across electrodes and twofolds of one cross-validated iteration. Colors correspond to orientations. ***B***, Correlations between actual and estimated responses averaged over three cross-validated iterations for all electrodes (gray, microelectrodes; red, ECoGs). Bars show the average correlation across electrodes. Error bars denote SEM. The distribution of parameters of the model can be observed in Extended Data Figure 3-1, and the correlations using individual parameters (corresponding to Fig. 3*B*) are presented in Extended Data Figure 3-2.

SVD analysis revealed high separability of the two-dimensional responses with separability indices close to 1 (Table 1) for both Size-Ori and Con-Ori protocols. The product models explained significantly more variance than the additive model (Fig. 2), but the *R*^2^ for the two product models were not significantly different from each other (statistics in Fig. 2). While the separability index was high for all electrodes, the explained variance was occasionally not very high, especially for Size-Ori protocol for M2. This was simply because the microelectrode RFs were very foveal for M2, and therefore the response saturated quickly and did not increase monotonically with size [see Extended Data Fig. 1-1 and Fig. 3*A* for gamma vs size; Dubey and Ray (2020), their Fig. 3*G*,*H*, for change in power spectra]. Indeed, ECoGs (blue trace for the size plot in Extended Data Fig. 1-1 and red points in Fig. 3*A*) had higher *R*^2^ values since their RFs were not foveal. However, even for this protocol, the multiplicative model performed much better than the additive one.

10.1523/ENEURO.0417-23.2024.f3-1 Figure 3-1 **Distribution of grating parameters.** Distribution of 8 parameters for all electrodes defining the shape of tuning functions to independent features of spatial frequency, orientation, size and contrast, shown for both monkeys. Note that while trials were divided into two halves for testing the model performance, here these parameters were obtained using all trials (averaging the parameters obtained from two halves also yielded similar results). The grey bars correspond to microelectrodes, and red ones to ECoGs. Medians across all electrodes are shown as triangular markers. Download Figure 3-1, TIF file.

10.1523/ENEURO.0417-23.2024.f3-2 Figure 3-2 **Performance with individual parameters.** The correlation between estimated and actual gamma responses when individual tuning parameters of electrodes were used instead of medians, for three grating protocols. Red dots correspond to ECoGs and grey to individual microelectrodes. The correlation values of each electrode are averaged over 3 iterations of cross-validation. Error bars correspond to SEM. Download Figure 3-2, TIF file.

#### Hue, saturation, and value

Responses to parametric chromatic stimuli were recorded from the right hemispheres of the same monkeys in a different set of experiments (Shirhatti and Ray, 2018) and are labeled as M1R and M2R. The gamma dependence on individual features (Hue, Sat, Val, Size) is shown in Extended Data Figure ure 1-2. This figure also displays responses from the left hemisphere to six full-screen hues. As shown previously, the tuning curves for gamma remain consistent across space (Dubey and Ray, 2020). Even across hemispheres, the tuning curve followed a similar shape for both LFP and ECoG scales (compare the dotted red and blue traces with the pink trace in Extended Data Figure . 1-2). We tested separability using full-screen protocols in which two features out of Hue, Saturation (Sat), and Value (Val) were varied. Hue-Sat protocol displayed six unique hues at five Sat levels, while the Hue-Val protocol used six hues with five luminance levels. Sat-Val protocol (only for M1R) used five levels of both saturation and value for red hue (see Materials and Methods). For all protocols, we constructed a two-dimensional matrix for each electrode from the mean change in power in the gamma range and then performed separability analysis. The results are summarized in Table 1 and Figure ure 2. The marginal additive model explained the least variance as in the case of grating stimuli. *R*^2^ values indicate that the first singular vectors could explain most of the data variance, and they were not significantly different from the *R*^2^ values of marginal product model (statistics in Figure . 2). For the Hue-Sat protocol of M1R, average *R*^2^ was lower than the other protocols, likely because at low saturation, responses were not well separable across hues for some electrodes (Figure . 4*A*, “Hue-Sat” plot, open circles) since the stimuli simply were white/dimmed irrespective of hue. Nonetheless, the main results—superior performance of product models compared with additive model and comparable performance of marginal and SVD based models—were observed for this case also.

**Figure 4.**
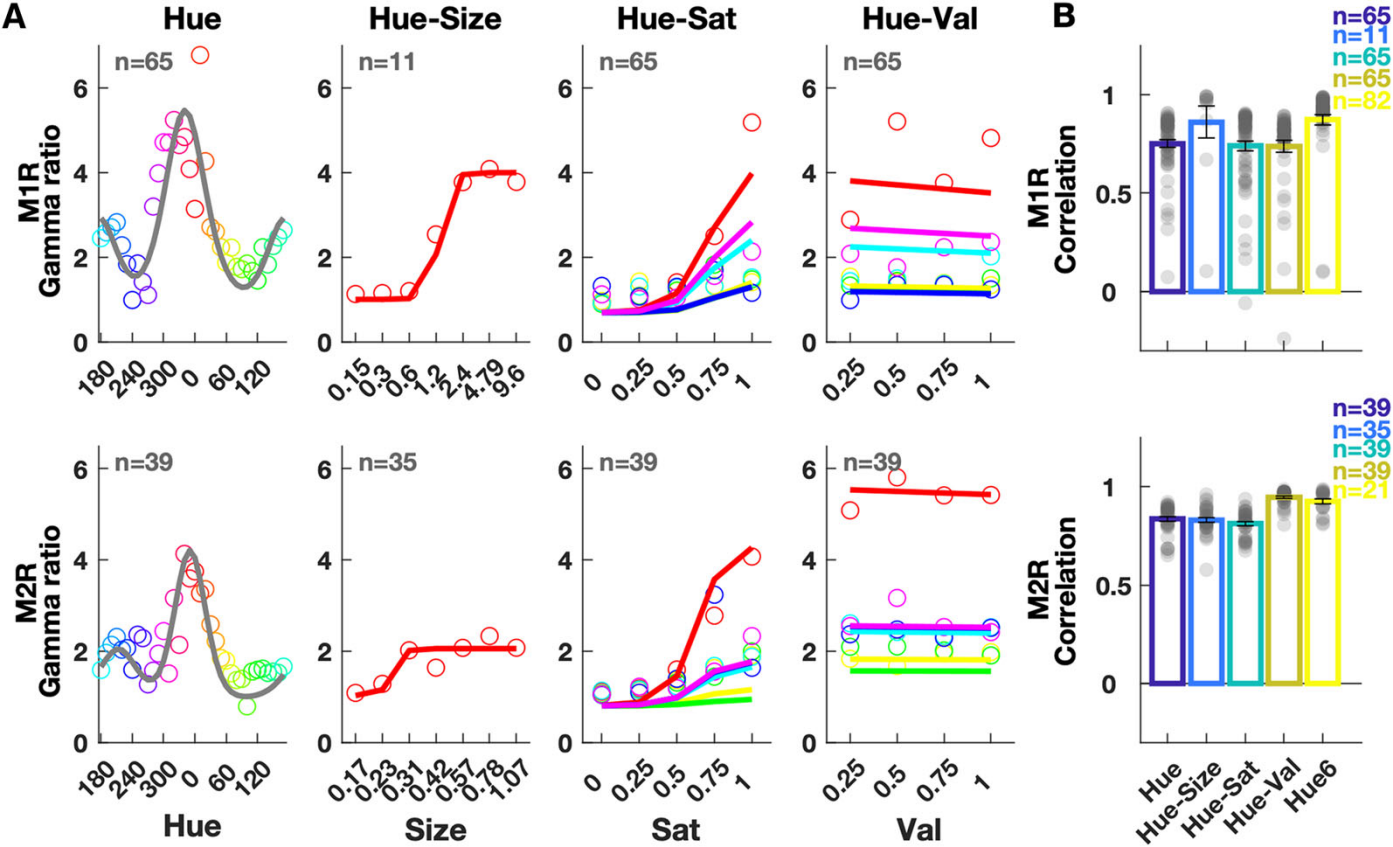
Gamma responses to hue stimuli and estimates. Response refers to the change in gamma band (30–80 Hz) power from baseline in 250–500 ms period after stimulus onset. ***A***, Actual response (open circles) and estimated responses to the Hue, Hue-Size, Hue-Sat, and Hue-Val stimuli, averaged across electrodes, and twofolds of a single cross-validated iteration. ***B***, Correlations between actual and estimated responses averaged over three cross-validated iterations for all microelectrodes (gray dots). Bars show the average correlation across electrodes. Error bars denote SEM. The last bar (Hue-6) uses data from the left hemisphere (M1, M2) to 6 full-screen hues, and the estimates are obtained using parameters learnt from the right hemisphere. The distribution of parameters of the model can be observed in Extended Data Figure ure 4-1, and the correlations using individual parameters (corresponding to Figure . 4*B*) are presented in Extended Data Figure ure 4-2.

10.1523/ENEURO.0417-23.2024.f4-1 Figure 4-1 **Distribution of hue parameters.** Distribution of 11 parameters for all electrodes defining the shape of tuning functions to independent features of hue, size, saturation and value, shown for both monkeys. All trials were used to calculate these parameters. Medians across all microelectrodes are shown as triangular markers. Download Figure 4-1, TIF file.

10.1523/ENEURO.0417-23.2024.f4-2 Figure 4-2 **Performance with individual parameters.** The correlation between estimated and actual gamma responses when individual tuning parameters of electrodes were used instead of medians, for the hue protocols. Grey dots correspond to individual microelectrodes. The correlation values of each electrode are averaged over 3 iterations of cross-validation. Error bars correspond to SEM. Download Figure 4-2, TIF file.

Overall, the separability results indicated that two-dimensional responses to parametric stimuli were largely separable and expressed as products of independent functions. These functions could be estimated from the one-dimensional tuning to individual features, as discussed next.

### Joint gamma responses can be modeled as product of individual responses

We modeled the independent feature tuning of gamma by functions reflecting the shape of the gamma tuning curves, utilizing the same data as used for the separability analysis. We did not systematically compare different function classes but chose simple functions popularly used for modeling such responses. We then extended the pairwise separability to all features and expressed the response as a multidimensional product.

#### Grating responses

We modeled the SF tuning as a Gaussian function on the SF values, with a center and a spread parameter (Eq. 6), and the orientation tuning as a von Mises function over the angular space (Eq. 7). Size and contrast were considered to be maximum at the full-screen size and 100% contrast, respectively, and were modeled as sigmoidal functions (Eqs. 8, 9). Each of these functions was described by two free parameters (see Materials and Methods).

Since the features—SF, contrast, and size—were separable from orientation, we extended this finding and expressed their joint response as a product of four individual functions (Eq. 10A; eight free parameters characterizing the centers and spreads for Gaussian and von Mises functions and the slopes and midpoints for the sigmoidal size and contrast functions). We first performed a parameter estimation procedure for each electrode separately, where trials from SF-Ori, Size-Ori, and Con-Ori protocols were divided into two folds, one of which was averaged to yield the gamma responses to calculate the model parameters. Specifically, to get the SF parameters, SF-Ori responses were pooled across orientations and fit to Equation 6 using least squares fitting. Similarly, responses were pooled across SFs to fit orientation parameters (Eq. 7) and so on. Subsequently, the predicted gamma response from the model was compared against the actual response obtained by trial-averaging the second fold.

Extended Data Figure ure 3-1 shows the electrode-wise parameters. As also reported in previous studies (Berens et al., 2008; Jia et al., 2011; Murty et al., 2018; Dubey and Ray, 2020), the parameters tended to cluster together allowing for a major simplification: we used a common set of parameters for all electrodes of each monkey by choosing the medians of these distributions. Therefore, all electrodes used the same shape of tuning functions resulting in a common estimated value (Eq. 10A). Electrode estimates could therefore only vary by gain and offset terms (Eq. 10B).

Figure ure 3*A* displays the actual gamma response (open circles) and the estimates (lines; using Eq. 10B) averaged across microelectrodes. Despite using the same parameters, most electrodes had highly correlated estimated and actual gamma responses, especially for SF-Ori and Con-Ori protocols (Figure . 3*B*, gray markers). The values were lower for Size-Ori protocol of M2, for which the data points (Figure . 3*A*, second row) did not smoothly increase with size. This was true of most microelectrodes (Figure . 3*B*, gray markers), extending from their lower Size-Ori separability (Figure . 2). Interestingly, the same model also predicted ECoG responses reasonably well (Figure . 3*B*, red markers). The performance using individual electrode parameters (Extended Data Figure . 3-2) was noticeably better for most ECoGs, but not microelectrodes. The medians were biased toward the microelectrodes because of their higher number compared with ECoGs. Although ECoG and microelectrode tuning preferences tend to be similar (Dubey and Ray, 2020), differences in RF size and location may cause some variation in the exact shape of the function. Despite this, the median parameters could represent feature dependencies of ECoG gamma reasonably well. The median parameters obtained by using all trials (not two folds) are shown in Table 2.

**Table 2.** Median values of parameters across electrodes obtained by using all trials

Parameter	M1	M2
SF center, *μ*	5.0196	3.0521
SF spread, *σ*	0.9413	1.0300
ORI center, *α*	109.3960	23.9108
ORI spread, *κ*	0.8362	0.1552
SIZE slope, *m*	0.3674	6.3396
SIZE midpoint, *σ*_0_	1.2904	0.3190
CON slope, *k*	1.4808	1.1180
CON midpoint, *c*_0_	22.8935	32.1019
HUE center R, *αr*	5.9789	6.2326
HUE spread R, *κr*	1.9068	4.2599
HUE center C, *αc*	3.0042	3.7618
HUE spread C, *κc*	2.5095	1.8884
HUE gain, *a_c_*	0.4335	0.4030
SIZE slope, *m*	1.6175	0.9333
SIZE midpoint, *σ*_0_	1.2120	0.2257
SAT slope, *l*	3.2922	4.2867
SAT midpoint, *s*_0_	0.7477	0.6463
VAL slope, *g_v_*	0.0807	0.00027
VAL intercept, *o_v_*	0.3472	0.4753

#### Hue responses

Apart from the stimuli used for separability analysis (Hue-Sat and Hue-Val), we also recorded protocols in which only hue (Hue protocol) or size was varied (Hue-Size protocol). In the Hue protocol, 36 full-screen hues were displayed at maximum saturation and value. Hue-Size protocol displayed red hue at seven sizes. Hue-Sat and Hue-Val protocols used six hues at five levels of saturation and value, respectively (see Materials and Methods). We first modeled the gamma dependence on individual features as one-dimensional tuning functions and then used the separability result to express the gamma response as a product of these functions.

The hue response was modeled as the sum of two von Mises functions, each with a center and a spread parameter (Eq. 11). The size and saturation were considered as scaling parameters which are maximum at full screen and full saturation. Their tunings were modeled as sigmoidal functions, each with a slope and a midpoint parameter (Eqs. 12, 13). The value dependence was modeled as a linear function (Eq. 14). We previously found that changing value above 25% caused no change in gamma response (Shirhatti and Ray, 2018), and since we did not sample the 0–25% interval, we skipped the first point (0%) for this fit. The slope of value dependence was close to 0 (the curve was almost flat, “Hue-Val” plot, Figure . 4*A*), so value had little effect on the gamma response. Reasons for this poor correlation between value and gamma responses are discussed later. For all protocols, trials were separated into two folds, parameters were obtained using one fold, and gamma responses from the other fold were correlated against the predicted gamma. Parameters obtained for different electrodes are shown in Extended Data Figure ure 4-1. Similar to the achromatic case, these values clustered together, so we chose their medians to represent all electrodes in Equation 15A, resulting in the same modeled response for all electrodes except differences in gain and offset. The gain and offset were individually estimated by fitting the function (Eq. 15B) with data from the other fold to obtain magnitude matched estimates (Figure . 4*A*). The linear correlation values between modeled response and data are shown in Figure ure 4*B*. Extended Data Figure ure 4-2 shows correlations obtained using individual electrode parameters. Further, we used the median parameters of this hue model to predict responses to six full-screen hues and correlated that to the recorded responses from the left hemisphere. The results are shown in the last bar of Figure ure 4*B*, and this model could predict the responses fairly well.

Once the model parameters were fixed for each monkey (Table 2), the gamma response to any achromatic grating specified by four parameters—SF, Ori, Con, and Size—was completely determined by Equation 10A. Similarly, response to any chromatic patch specified by four parameters—Hue, Sat, Val, and Size—was determined by Equation 15A. Different electrodes could vary only in gains and offsets (Eqs. 10B, 15B) but that does not change the correlation between actual and predicted gamma responses.

### Prediction of image gamma responses

In the natural images protocols, 16 natural images from one of five categories (a total of 80 images) were displayed on the full screen (see Materials and Methods and Kanth and Ray, 2020). One or two categories (16 or 32 images) were displayed in a single protocol. We also used grayscale versions of these images. To predict gamma responses to an image, we checked whether image patches around the RF of an electrode could be represented by a two-dimensional Gabor or a uniform hue patch. In all, we analyzed 8,240 patches [80 images × (82 + 21) electrodes] for a potential match with a grating or a chromatic patch. Since 16 images occurred per category, these patches were distributed across 515 (5 categories × (82 + 21) electrodes) electrode-category sets.

We first approximated the grayscale image patches to Gabors having SF, orientation, size, and contrast features. We masked the image by a spatial Gaussian of increasing sigma (starting from 0.3°) centered at the electrode's RF and performed a two-dimensional Fourier transform to identify the dominant SF and orientation within each size before applying some selection criteria (see Materials and Methods). However, since natural images have a slow changing feature profile (higher power at low SFs), only a small fraction (1 ± 1.3 out of 16, averaged over electrode-categories) could be approximated with Gabors. We therefore could perform further analysis only for hue patches of colored stimuli, as described below.

For the colored images, we centered disks of iteratively increasing radius (starting with 0.3°) on the RF center and calculated the means and standard deviations of hue, saturation, and value of pixels contained within the enclosed rings. We then selected a radius (*R*) for which these quantities varied within thresholds (see Materials and Methods). Patches that did not qualify at the smallest size were assigned a radius of zero. Using pixels within the patch of radius *R*, we calculated the mean hue (*H*, Eq. 16), mean saturation (*S*, Eq. 17), and average value (*V*) to represent the uniform hue patches. Most images were approximated by at least a small patch. We also tested whether the Value layer could be approximated with a Gabor, as described before.

Images from the Flora category, their magnified sections, and approximations for a typical electrode from M1 and M2 are shown in Figure ure 5. Image 4 for M1 and Image 5 for M2 were classified as Gabors based on the Value layer, but since it was not achromatic, gamma response could not be predicted from Equation 10A. Nonzero radii were obtained for 10 and 12 of the remaining images of M1 and M2, respectively. Using the radius and average hue, saturation, and value with Equation 15A gave an estimated gamma response for these images (Figure . 5*D*, selected set, filled circles). For images with a patch size of 0 (Images 1, 3, 6–8 in Figure . 5*B* and Images 1, 7 and 8 in Figure . 5*C*), the model prediction was trivially 0 (Figure . 5*D*, open circles). The frequency domain response (Figure . 5*B*,*C*, bottom row) shows gamma peaks for some images. Linear correlation between the actual and estimated gamma response was calculated using all 16 images (*r*Full = 0.58 and 0.56 for the two electrodes; Figure . 5*D*) as well as the selected set of images (*r*Sel = 0.58 and 0.8). Note that the gain and offset calculation (Eq. 15B) would simply change the plot (Figure . 5*D*) to make the points match in magnitude, but not change the correlations. Also, splitting the data for training and testing is not needed since the model parameters have already been estimated solely using the parametric data.

**Figure 5.**
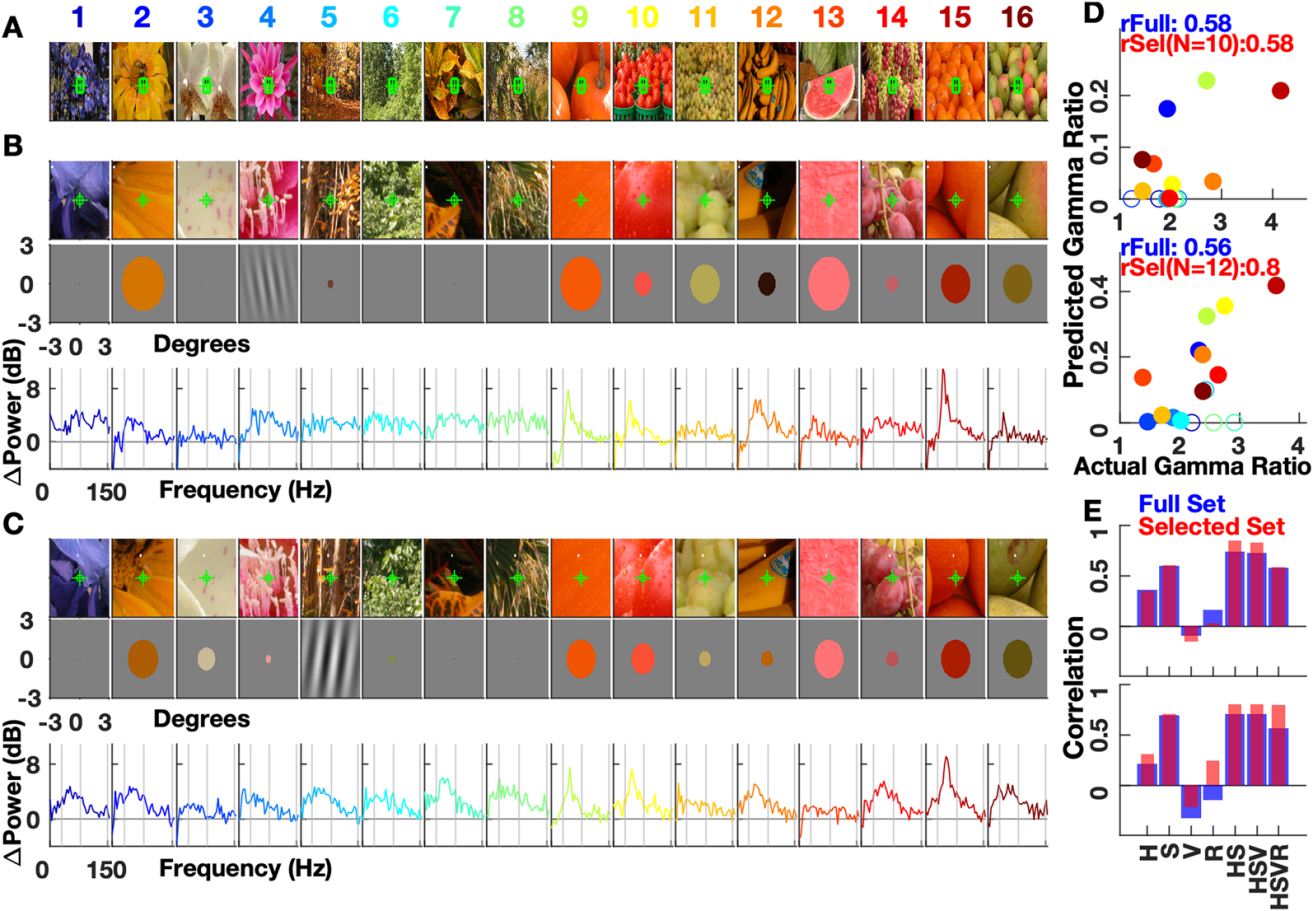
Response predictions for typical electrodes (M1 e34, M2 e7). ***A***, 16 stimulus images from Flora image set. ***B***, 3 × 3 degree patches around the RF of electrode 34 of M1. The RF center is marked by a green cross. The second row shows the approximations of patches. The bottom row shows the log change in power from baseline in the 250–500 ms period after stimulus onset as obtained by Multitaper analysis. Vertical lines denote 30, 80, and 150 Hz. ***C***, Same as ***B*** for M2 electrode 7. ***D***, Predicted gamma (30–80 Hz) responses versus actual responses for M1 (top) and M2 (bottom). Response refers to the change in gamma power from baseline. The filled data points correspond to images that are selected as patches in ***B*** and ***C***. rFull is the correlation for all 16 images with unselected patches getting a prediction of 0, and rSel uses only the selected patches. ***E***, Correlations for full and selected set when chosen patch features [on *x*-axis: H(ue), S(aturation), V(alue) and R(adius)] were used with the model for M1 (top) and M2 (bottom). The other features were given maximum values, or 0° in case of Hue. The HSVR bar corresponds to data shown in ***D***.

To check how individual features affect the response estimate, we input only selected features to the model, keeping others constant. For instance, using only the correct saturation (*S*), and keeping hue at red (0°), value at maximum (1), and a large size (10°), the correlation across 16 images was 0.59. The correlations for different parameter combinations are shown in Figure ure 5*E*, for both the full and the selected set. The last bar (HSVR) corresponds to the correlation values shown in Figure ure 5*D*.

### Population results for image response prediction

We analyzed image patches at all electrode RFs for uniform HSV features. These features were used to obtain the response estimate, and the correlation with actual gamma responses was calculated for each electrode and category. The first row in Figure ure 6 shows the average correlation using different feature combinations in the model (like Figure . 5*D*). The “Full set” used all 16 images per category [M1:82 × 5, M2:21 × 5 (electrodes × categories)]. We then dropped patches with size of 0 or a Gabor match to get correlation for the “selected set.” Out of 8,240 patches, 4,739 qualified as chromatic patches with an average of 9.20 ± 2.8 SD (of 16) patches per electrode-category while only 1.13 ± 1.3 SD had a Gabor approximation. To eliminate trivial correlations arising due to fewer data points, we only selected electrode–category combinations for which ≥8 (of 16) images qualified as patches (Figure . 6, selected set). For M1, 257 out of 410 and for M2, 74 out of 105 electrode categories were selected.

**Figure 6.**
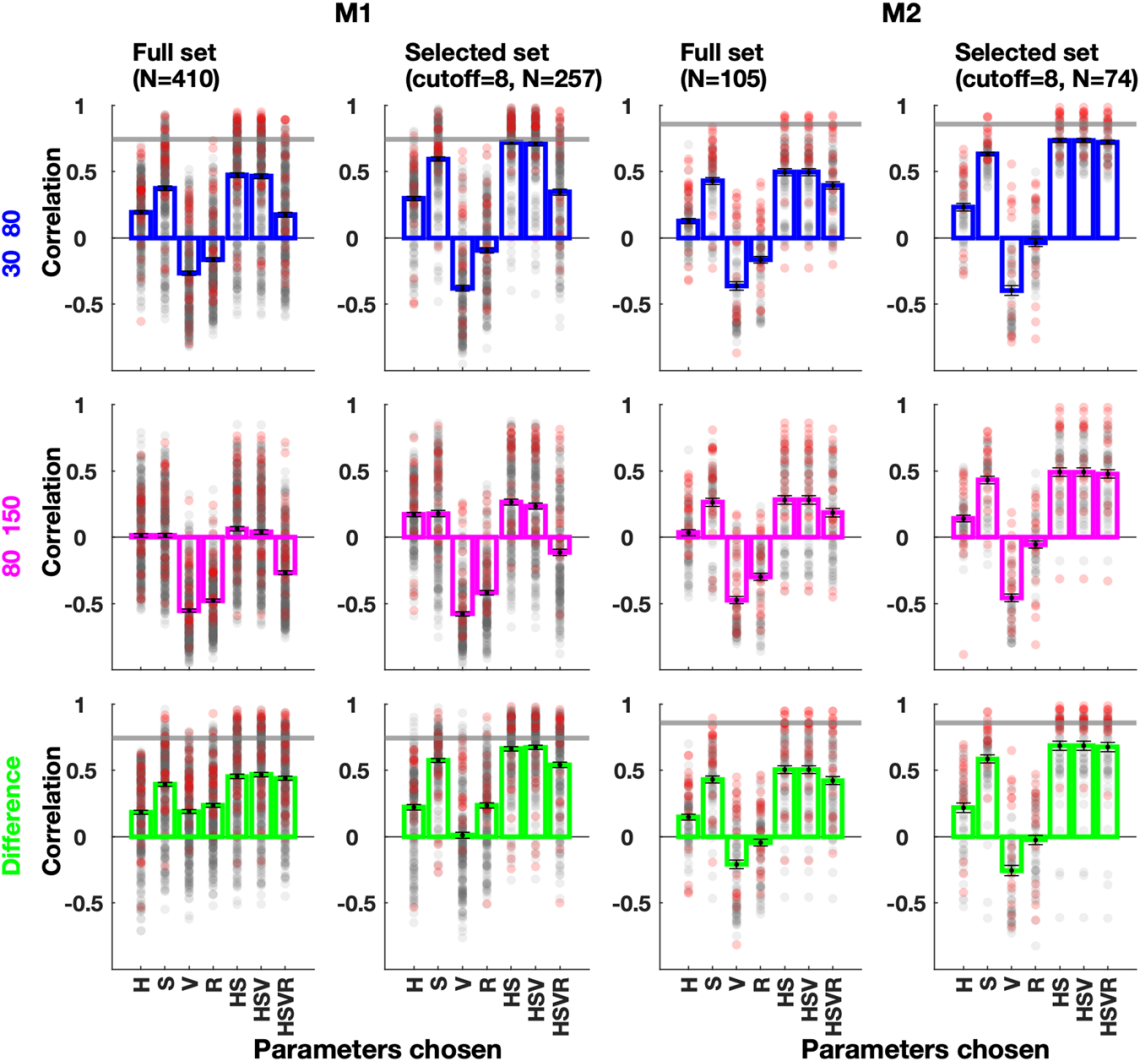
Response correlations across electrodes. The correlations between actual response and predicted response modeled with one or more image features. Actual response was chosen as change in power in gamma (30–80 Hz, blue) or high gamma (80–150 Hz, magenta) range. The third row (green) is the correlation of predicted response with difference between the actual gamma and high gamma response. All sessions [5 image sets × (82 or 21 electrodes)] are averaged (full set). The second and fourth columns (selected set) only average sessions with ≥8 (out of 16) images selected as patches. Markers show performance of individual electrodes. The horizontal gray lines are the average correlation (across protocols) achieved by the hue model when applied on parametric patches as shown in Figure ure 4.

Interestingly, using only value (*V*) or size (*R*) resulted in negative correlations. While HS features could predict responses fairly well, addition of R caused a reverse effect, especially for M1. This is because we calculated gamma response as change in power from baseline but that allows for higher responses even with a wideband pedestal-like increase without an actual gamma peak (e.g., Images 5 and 14 vs 9, 10, 15 in Figure . 5*B*). The reason for this discrepancy is straightforward: image patches tended to be represented as small hue patches when there were salient changes around the RF (e.g., edges), which tended to elicit strong firing. Firing rates have been associated with a broadband spectral response prominent in the high-gamma range (Ray and Maunsell, 2011). Therefore, small radii as well as dark patches tended to produce higher broadband responses, leading to a negative correlation between *V* and *R* parameters and the gamma response.

One solution is to separate the broadband pedestal and the oscillatory response, using recent techniques like FOOOF (Donoghue et al., 2020). However, since the gamma oscillatory response was ambiguous for many images, such separation could depend on the inputs given to the algorithm. We instead followed a simpler strategy: we approximated the wideband response by calculating the change in power in the high-gamma band (80–150 Hz). As expected, the correlations between estimates and high-gamma response were more negative for the *V* and *R* only models (magenta bars). To counter the effect of the broadband response, we modified the actual response by taking the difference of the gamma and the high-gamma response. The correlation of this modified response with the estimates (green bars) did not show a steep decrease upon including the size parameter but did not improve the prediction either.

Overall, the hue and saturation accounted for the most contribution to gamma response, as seen by the HS bars. The correlations for the selected set and HS model were 0.66 ± 0.27 SD (M1) and 0.69 ± 0.3 SD (M2), while the correlations obtained on parametric hue patches were 0.74 ± 0.21 SD and 0.86 ± 0.08 SD (averages of points in Figure . 4*B*, gray lines in Figure . 6). “Value” did not make a significant contribution as expected since gamma versus value was almost flat (Figure . 4*A*). Size also did not improve the performance partly because the response saturated (Figure . 4*A*) and partially due to the confounding effect of the broadband response.

The patch parameters depended on the thresholds on the means and standard deviations of *H*, *S*, and *V* parameters: using stringent thresholds leads to smaller radii in general. Since gamma dependence on value was minimal, we had a more relaxed threshold for the *V* parameter. This can be observed in Images 15 and 16 in Figure ure 5*B*, where the estimated patches seem to be larger than expected because the discontinuities were mainly in the “Value” layer. However, since the size did not improve prediction, we also used a basic model in which all patches had a fixed size, over which average *H* and *S* were computed as before, and gamma response was estimated using Equation 15A. This basic model also performed reasonably well. For example, for fixed radii of 0.5, 1, and 2°, the respective average correlations for the selected set (same images and electrode-categories as selected with variable radii for Figure . 6) were 0.67 ± 0.26 SD, 0.67 ± 0.26 SD, and 0.66 ± 0.26 SD for M1 and 0.66 ± 0.32 SD, 0.68 ± 0.30 SD, and 0.60 ± 0.32 SD for M2, as compared with 0.66 ± 0.27 SD and 0.69 ± 0.30 SD when *H* and *S* were computed over variable radii. This shows that even a simple model using only the average hue and saturation of a patch around the RF can estimate the gamma response with reasonably high accuracy.

## Discussion

We found that gamma responses to two stimulus features are largely separable, for both LFP and ECoG scales, and could be modeled as a product of individual feature dependencies. We further developed a feature-based image-computable model which could predict the gamma responses to chromatic images with reasonably high correlation.

Joint tuning of V1 neuronal responses has been studied for SF and orientation (Vidyasagar and Sigüenza, 1985; Mazer et al., 2002; Bressloff and Cowan, 2003; Nauhaus et al., 2012; Ayzenshtat et al., 2016) and features like disparity and orientation or direction selectivity (Grunewald, 2004), with varying conclusions about separability. While membrane potentials (Peña and Konishi, 2001) and fMRI responses (Leoné et al., 2014) have been investigated for separability, LFP responses have not. Our results show that the LFP gamma tuning to low-level features of luminance gratings and chromatic patches is largely separable. The separability is maintained across scales as evidenced by the ECoG responses which record from a larger neuronal population. Quantifying the separability also raises questions about data not explained by separable models. This “inseparable component” could result from biophysical or experimental noise but could indicate other interactions, e.g., subtle shifts in one preferred feature arising from changes in another (as reported in mice; Ayzenshtat et al., 2016), or nonlinear processes contributing to extracellular potentials. Such nonlinearities can potentially be studied via models explaining gamma oscillations through nonlinear interactions between excitatory and inhibitory cortical activity (Wilson and Cowan, 1972; Jadi and Sejnowski, 2014). The separability results are surprising, since gamma response was simply the summed power over a fixed (30–80 Hz) band, ignoring the gamma peak frequency shifts that occur with stimulus feature changes such as contrast.

We developed a simple model to estimate responses from stimulus features for luminance gratings and hue patches. The model tuning parameters were consistent across electrodes, allowing us to use their medians. Using electrode-wise parameters might improve the model performance (Extended Data Figure s. 3-2, 4-2), but we traded accuracy for simplicity, generalizing our findings over a cortical area, across scales (LFP and ECoG), and indeed across hemispheres (hue parameters). For the color model, this simplification was necessary since the model was trained on recordings from one brain hemisphere and used to predict responses recorded later from the other hemisphere.

Image-computable models of gamma predict responses to novel images. Earlier attempts in the grayscale space have used luminance contrast as a primary feature. While some studies have shown gamma depends on grayscale image features (N. M. Brunet and Fries, 2019), others have further predicted the response based on luminance features like orientation (Hermes et al., 2019) or by using neural networks (Uran et al., 2022). Though the effects of color are now being studied, to our knowledge, this is the first attempt at an image-computable gamma model based on chromatic features. Our approach to obtain patches may seem similar to uniformity (N. M. Brunet and Fries, 2019; Peter et al., 2019) and predictability (Uran et al., 2022). However, we start analyzing the image at the RF whereas Uran and colleagues excluded the RF. We also use the OV metric of (Hermes et al., 2019), not to calculate gamma but instead as a selection criterion for grating approximations.

Nevertheless, there are several simplifications and limitations of this model. First, the gamma peak frequency is known to vary with stimuli, e.g., increasing with contrast (Dubey and Ray, 2020), which we did not take into account. Second, our model does not account for some key drivers of neural activity, such as the luminance change between background and stimulus. Further, the “value” in the HSV space varies between 0 and 1 for all hues but corresponds to different luminance for each hue. For example, the interstimulus gray screen (value = 0.5) had a luminance of 60 cd/m^2^ in our experiments, while the same value (=0.5) for blue, red, and green primaries had luminance of 3.5, 13, and 43.5 cd/m^2^ [see Shirhatti and Ray (2018), their Table S1, for stimulus CIE (CIE TC 1-85, 2018) (*x*, *y*, *Y*) coordinates; *Y* is the luminance]. While neural activity and gamma response depend on the luminance change, it is not fully captured by the value parameter in HSV space. A better alternative is to convert colors to the physiologically more relevant DKL space (Derrington et al., 1984) but is beyond the scope here since we did not have responses for stimuli varying along the DKL space. There is some evidence (Stauch et al., 2022) that when using parametric DKL colors, human MEG gamma is not as strongly biased toward reddish hues, but whether the same holds for V1 LFP gamma responses remains to be tested.

Further, gamma responses to at least two more classes of parametric stimuli are required to properly use our model. The first are achromatic stimuli with mean luminance different from the pre-stimulus gray. Our parametric achromatic stimuli had the same mean luminance of 60 cd/m^2^ as the prestimulus gray screen (e.g., a 50% contrast grating would have luminance varying between 30 and 90 cd/m^2^ across space). However, a natural image can be represented by a 50% contrast grating having a different mean luminance (e.g., a grating whose luminance varies between 10 and 30 cd/m^2^ also has 50% contrast but mean luminance of 20 cd/m^2^). When such a grating follows a gray screen of 60 cd/m^2^, the resulting response will be due to two factors—a mean luminance change from 60 to 20 cd/m^2^ and a spatial contrast variation of 50%. But gamma dependence on luminance and particularly on the combination of contrast and mean luminance remains unknown. We could have predicted gamma responses to most achromatic images (since they were mainly grayscale uniform patches) had we known the responses for gray at varying luminance but we did not record this data.

The second stimulus class involves chromatic luminance gratings (like the magenta and white stripes in image 4 of Figure . 5). These are gratings with a particular hue (say red) whose luminance varies with space (generating red-and-black or red-and-white gratings). How the hue, contrast, and other factors like orientation combine to produce a gamma response is also unknown. Though previously considered separate pathways, joint coding of color and orientation by V1 neurons has been reported (Garg et al., 2019) and may affect the population (gamma) response also. Note that these are different from the iso-luminant chromatic gratings (red-and-green of equal luminance) used in the DKL space. Future studies where these classes of parametric stimuli are also used will allow us to predict a larger set of natural images.

Despite these limitations, we show that a simple model based on mainly the hue and saturation of the pixels inside/around the RF can predict the gamma responses of color images reasonably well. A better understanding of gamma dependence on other classes of parametric stimuli as well as other image-derived measures (like predictability and compressibility) will shed new light on the biophysical mechanisms underlying gamma generated by natural images and their potential role in coding or cognition.

## Synthesis

Reviewing Editor: Fabienne Poulain, University of South Carolina Decisions are customarily a result of the Reviewing Editor and the peer reviewers coming together and discussing their recommendations until a consensus is reached. When revisions are invited, a fact-based synthesis statement explaining their decision and outlining what is needed to prepare a revision will be listed below. The following reviewer(s) agreed to reveal their identity: Brett Foster. Note: If this manuscript was transferred from JNeurosci and a decision was made to accept the manuscript without peer review, a brief statement to this effect will instead be what is listed below.

Your manuscript has been evaluated by two reviewers with expertise in visual processing. As you will see in their comments summarized below, both reviewers agree that while your study is significant and addresses an important question, the specifics of the methodological approach are extremely difficult to comprehend and contain many analytic caveats in data processing. Therefore, the presentation of the work requires substantial improvement in order to fully assess its scientific merits and to be considered for publication in eNeuro.

Comments from the reviewers:

In their work, Kanth and Ray seek to quantify the joint tuning properties of visual gamma responses across key features of achromatic gratings and chromatic hue patches in the macaque. Next, they aimed to create image computable models such that for a given receptive field and natural image content, the associated gamma response could be predicted and compared to recorded responses. While much of the experimental observations have previously been documented (many by the authors) regarding low-level feature tuning of gamma, along with image-based estimation for achromatic stimuli (noted above), there have been no systematic models of how low-level hue features of stimuli influence visual gamma responses to complex stimuli, and therefore how to model observations of structural and color related features jointly modulate such responses. It is this domain that is the new contribution of the authors work. Overall, while addressing an important question, the specifics of the methodological approach are difficult to follow and contain many analytic caveats in data processing. It appears that ultimately, the authors are unable to use their tuning data from achromatic stimuli (much of the paper) and can only apply their hue-based model to a small subset (<16) of natural images. In addition, hue screening experiments come from prior data recorded in the opposing visual hemisphere. Therefore, it is not clear if any of the joint tuning data make it into the authors natural image predictions, leading to the authors title prediction of low-level hue driving natural image gamma responses (which has been empirically shown previously by the authors Kanth &Ray 2020 and others; Bartoli et al. 2019).

Major points:

-The manuscript methodology is difficult to follow given the many details of visual stimulus features and analysis decisions, without any supporting figures or schematics. Given the central focus on stimulus feature tuning, it is essential that all stimuli/features are shown early in the manuscript (currently only natural images shown in figure 5) to provide the reader with a clear concept for what is shown and being manipulated. Similar work in the field (Hermes et al. 2019; Uran et al. 2022) provides good examples for depicting the important stimulus-response mappings (i.e. stimulus, receptive field, predicted response, observed response). For example, the authors are trying to link low-level feature tuning to responses observed during natural images - but there are no figures showing this primary data. Finally, figure 5 provides the type of stimulus-response data that would greatly benefit the reader, but is only shown for one animal, both monkeys should be shown.

-The authors state "... only a small fraction (1 +/- 1.3 out of 16, averaged over electrode-categories) could be approximated with Gabors. We therefore could perform this analysis only for hue patches of colored stimuli, as described below." Does this mean none of the achromatic (grating) tuning data is used for predicting natural image gamma responses, only the hue model? If true, this seems to render much of the other methods/results documenting the tuning for achromatic stimuli useless towards the author's goals (much of the manuscript). Furthermore, if only the hue model is employed for predicting gamma responses to natural images this data: i) is based on hue screening responses recorded in different experiment/hemisphere from natural image experiments; ii) using a simple hue model for 16 images - with clear results only occurring for stimuli containing large uniform patches of longer wavelength color (red/orange). Given prior work showing these properties experimentally (Kanth &Ray 2020; Bartoli et al. 2019), which includes the author's own analysis of the same dataset, and that ultimately no join-tuning is used for modeling responses, it is not clear how the goals of the study outlined in the introduction are achieved, beyond replicating color tuning of gamma.

- clarification of the methods (with figures) and to what extent the joint tuning analysis (achromatic &chromatic) informs predictions of natural image gamma responses is critical to assessing the author's work.

- The majority of experimental techniques, recording sites, visual stimuli, and analysis methods are referenced to the authors' previous papers. Consequently, understanding the experimental methods solely from reading this paper is challenging. It is imperative that the paper be presented in a manner that can be comprehended independently. Attention should also be given to the order of figure mentions in the main text, and technical terms should not be used without proper definitions (e.g., L709: OV what?). All parameters in formulas should be explained.

- It is strongly recommended that the authors enlist the services of English editing before resubmitting their manuscript.

## Author Response

Synthesis of Reviews:

Synthesis Statement for Author (Required):

Your manuscript has been evaluated by two reviewers with expertise in visual processing. As you will see in their comments summarized below, both reviewers agree that while your study is significant and addresses an important question, the specifics of the methodological approach are extremely difficult to comprehend and contain many analytic caveats in data processing. Therefore, the presentation of the work requires substantial improvement in order to fully assess its scientific merits and to be considered for publication in eNeuro. >> We thank the editorial team and the reviewers for the feedback. We have made several changes to improve the presentation of our results.

Comments from the reviewers:

In their work, Kanth and Ray seek to quantify the joint tuning properties of visual gamma responses across key features of achromatic gratings and chromatic hue patches in the macaque. Next, they aimed to create image computable models such that for a given receptive field and natural image content, the associated gamma response could be predicted and compared to recorded responses. While much of the experimental observations have previously been documented (many by the authors) regarding low-level feature tuning of gamma, along with image-based estimation for achromatic stimuli (noted above), there have been no systematic models of how low- level hue features of stimuli influence visual gamma responses to complex stimuli, and therefore how to model observations of structural and color related features jointly modulate such responses. It is this domain that is the new contribution of the authors work. Overall, while addressing an important question, the specifics of the methodological approach are difficult to follow and contain many analytic caveats in data processing. It appears that ultimately, the authors are unable to use their tuning data from achromatic stimuli (much of the paper) and can only apply their hue-based model to a small subset (<16) of natural images. >> The reviewers raise an important point about using achromatic and colour models together. However, in this study, we have not jointly used the achromatic and chromatic models. For both model-building and prediction, the chromatic and achromatic stimuli are treated separately. The study had three aims - (1) to test the separability of features, (2) to build a multiplicative model for parametric stimuli (both chromatic hue patches and achromatic gabors), and (3) use the multiplicative model to predict gamma response for natural images which could be approximated as hue patches or achromatic Gabors. For the first part, the joint tuning was studied for both achromatic features (like spatial frequency and contrast) and chromatic features (like hue and saturation). Similarly, the multiplicative model was built for both achromatic and chromatic parametric stimuli separately. It is only the third aim for which achromatic images could not be used, since those images could be rarely approximated by achromatic Gabors that were used in part 2.

The hue based model was applied to 16 images per category, but there were 5 such categories (80 images in total), and we predicted the gamma responses for each electrode (the image patches for different electrodes were slightly different because each patch was centred on the receptive field of that electrode). Since we had 103 electrodes (82 + 21 for the two monkeys), we had a total of 103 x 80 = 8240 image patches. We found that out of these, 4739 image patches could be approximated as hue patches and hence the multiplicative model could be used to predict the responses. Hence, starting with a set of 16 image patches (there were 103 x 5 such sets; one set is shown in Figure 5), gamma response could be predicted for 9.2 (4739/8240 x 16) of these 16 image patches on average. Therefore, the prediction was actually tested for a large number of patches. We have now presented these numbers in the Results section under "Prediction of image gamma responses" and "Population results for image responses".

In addition, hue screening experiments come from prior data recorded in the opposing visual hemisphere. Therefore, it is not clear if any of the joint tuning data make it into the authors natural image predictions, leading to the authors title prediction of low- level hue driving natural image gamma responses (which has been empirically shown previously by the authors Kanth &Ray 2020 and others; Bartoli et al. 2019). >> The reviewer correctly notes that the hue tuning data comes from the right hemisphere. The motivation to use this was that we had tested a much larger number of colour stimuli for these recordings compared to subsequent recordings from the left hemisphere using the hybrid grid. We had previously shown that gamma tuning remained highly consistent across different locations (Dubey and Ray, 2020), and therefore used the same tuning across hemispheres as well. Now, in order to confirm that the tuning is indeed consistent across hemispheres, we have included a new dataset from the left hemisphere where we displayed 6 hues. Indeed, the tuning across hemisphere is highly consistent, as shown in our additional extended figure (1-2). Furthermore, we used the hue model parameters from the right hemisphere (previous recordings) to predict responses to six full screen hues in the left hemisphere (later recordings), and our model was successful in doing so (yellow bar in the new Figure 4B).

Regarding the question of whether the joint-tuning data make it into the predictions - it indeed does because the joint tuning results are used to build the multiplicative chromatic tuning model (Equation 15A). After this model is built (i.e., the free parameters in Equation 15A are estimated using only the parametric stimuli), once the H,S,V and Radius values of a chromatic patch are specified, the gamma response can be predicted using Equation 15A. Therefore, for natural images, we just need to find a chromatic patch (i.e., HSVR values) that approximates the image patch within the RF (as shown in Figure 5). Our previous studies only showed the tuning for individual features, not the multiplicative model which is used for gamma prediction for natural images.

Major points:

-The manuscript methodology is difficult to follow given the many details of visual stimulus features and analysis decisions, without any supporting figures or schematics. Given the central focus on stimulus feature tuning, it is essential that all stimuli/features are shown early in the manuscript (currently only natural images shown in figure 5) to provide the reader with a clear concept for what is shown and being manipulated. Similar work in the field (Hermes et al. 2019; Uran et al. 2022) provides good examples for depicting the important stimulus-response mappings (i.e. stimulus, receptive field, predicted response, observed response). For example, the authors are trying to link low-level feature tuning to responses observed during natural images - but there are no figures showing this primary data. Finally, figure 5 provides the type of stimulus-response data that would greatly benefit the reader, but is only shown for one animal, both monkeys should be shown. >> We thank the reviewer for pointing this out. We have added primary data that shows the dependence of gamma on different features in two extended figures that support Figure 1. We have also added data from a representative electrode for the second monkey to Fig 5.

-The authors state "... only a small fraction (1 +/- 1.3 out of 16, averaged over electrode-categories) could be approximated with Gabors. We therefore could perform this analysis only for hue patches of colored stimuli, as described below." Does this mean none of the achromatic (grating) tuning data is used for predicting natural image gamma responses, only the hue model? If true, this seems to render much of the other methods/results documenting the tuning for achromatic stimuli useless towards the author's goals (much of the manuscript). >> We thank the reviewer for the comment and the question. The grating data is not used for predicting the image responses because too few images had the required features to properly assess the correlations of the estimates. However the analysis and model generation for achromatic grating stimuli is an independent result highlighting the feasibility of a multiplicative model. We used it to successfully predict responses to parametric gratings (Figure 3B). Cross validation was done so that the predictions were done on data unseen by the model.

The inability to use this model for achromatic images simply stems from the fact that natural images have a dominant "1/f" spatial frequency spectrum (see (Kanth and Ray, 2020), Figure 15A), due to which very few of them had dominant grating-like features. However, our cross validated results on parametric stimuli open up the possibility of using such a feature based model on a better suited grayscale image set which would have more grating like spatial components.

Furthermore, if only the hue model is employed for predicting gamma responses to natural images this data: i) is based on hue screening responses recorded in different experiment/hemisphere from natural image experiments; ii) using a simple hue model for 16 images - with clear results only occurring for stimuli containing large uniform patches of longer wavelength color (red/orange). Given prior work showing these properties experimentally (Kanth &Ray 2020; Bartoli et al. 2019), which includes the author's own analysis of the same dataset, and that ultimately no join-tuning is used for modeling responses, it is not clear how the goals of the study outlined in the introduction are achieved, beyond replicating color tuning of gamma. >> Regarding (i) - we agree that the hue tuning data and image data was recorded from different hemispheres. But the hue preference of V1 gamma remains preserved across hemisphere and indeed across scales (see the added extended figure 1-2).

Being able to learn the hue tuning parameters from one hemisphere and utilise them on the second hemisphere actually is an advantage that can allow us to build more robust and generalizable models which depend largely on the image statistics and not on the exact site within V1.

Regarding (ii) - it is important to note that for response prediction, we have used joint tuning and included the effect of saturation, value and size in addition to hue, and therefore it is not just a replication of the hue results. Our goal was to predict the response based on simple features extracted from the image, and for these chromatic images we achieved that. The multiplicative model predicts response based on tuning parameters learnt for all the features. The hue response is heavily modulated by the saturation of the pixels. While we found that 'Value' and 'Size' features did not affect the prediction as much, the reasons for this are explored in the results under "Population results for image responses".

We would also like to point out that while Figure 5 shows only 16 images, it is a representative image for an example electrode. Overall, we had 5 sets of 16 images and 103 electrodes (82 for M1, 21 for M2) on which the analysis was carried out, and the overall results are presented in Figure 6.

- clarification of the methods (with figures) and to what extent the joint tuning analysis (achromatic &chromatic) informs predictions of natural image gamma responses is critical to assessing the author's work. >> We thank the reviewer for the comment. We have presented the Methods in more detail to increase clarity. We would like to point out that the joint tuning is not between achromatic and chromatic features. For both the model-building and the prediction, the chromatic and achromatic stimuli are treated separately. The joint tuning comes from using several achromatic features (like spatial frequency and contrast) together for achromatic stimuli, or several chromatic features (like hue and saturation) for colored stimuli.

- The majority of experimental techniques, recording sites, visual stimuli, and analysis methods are referenced to the authors' previous papers. Consequently, understanding the experimental methods solely from reading this paper is challenging. It is imperative that the paper be presented in a manner that can be comprehended independently. Attention should also be given to the order of figure mentions in the main text, and technical terms should not be used without proper definitions (e.g., L709: OV what?). All parameters in formulas should be explained. >> We thank the reviewer for these suggestions, and they have been included. We have expanded the experimental techniques and the description of visual stimuli to reduce the dependence of this text on our earlier work. The description of analysis methods have also been reworded to increase clarity.

OV stands for Orientation Variance. It is first mentioned in the Introduction L65, and therefore has not been expanded later.

- It is strongly recommended that the authors enlist the services of English editing before resubmitting their manuscript. >> We thank the reviewer for the suggestion. The manuscript (especially the Materials and Methods section) has been edited and many sentences have been reworded to improve the clarity and grammar.

References:

Dubey A, Ray S (2020) Comparison of tuning properties of gamma and high-gamma power in local field potential (LFP) versus electrocorticogram (ECoG) in visual cortex. Sci Rep 10:5422.

Kanth ST, Ray S (2020) Electrocorticogram (ECoG) Is Highly Informative in Primate Visual Cortex. J Neurosci 40:2430-2444.
