# Supplementary figures and images for: Gamma Responses to Colored Natural Stimuli Can Be Predicted from Local Low-Level Stimulus Features
Source: eNeuro. 2024 Jul 22;11(7):ENEURO.0417-23.2024. doi: 10.1523/ENEURO.0417-23.2024 (PMC11277289; doi:10.1523/ENEURO.0417-23.2024)

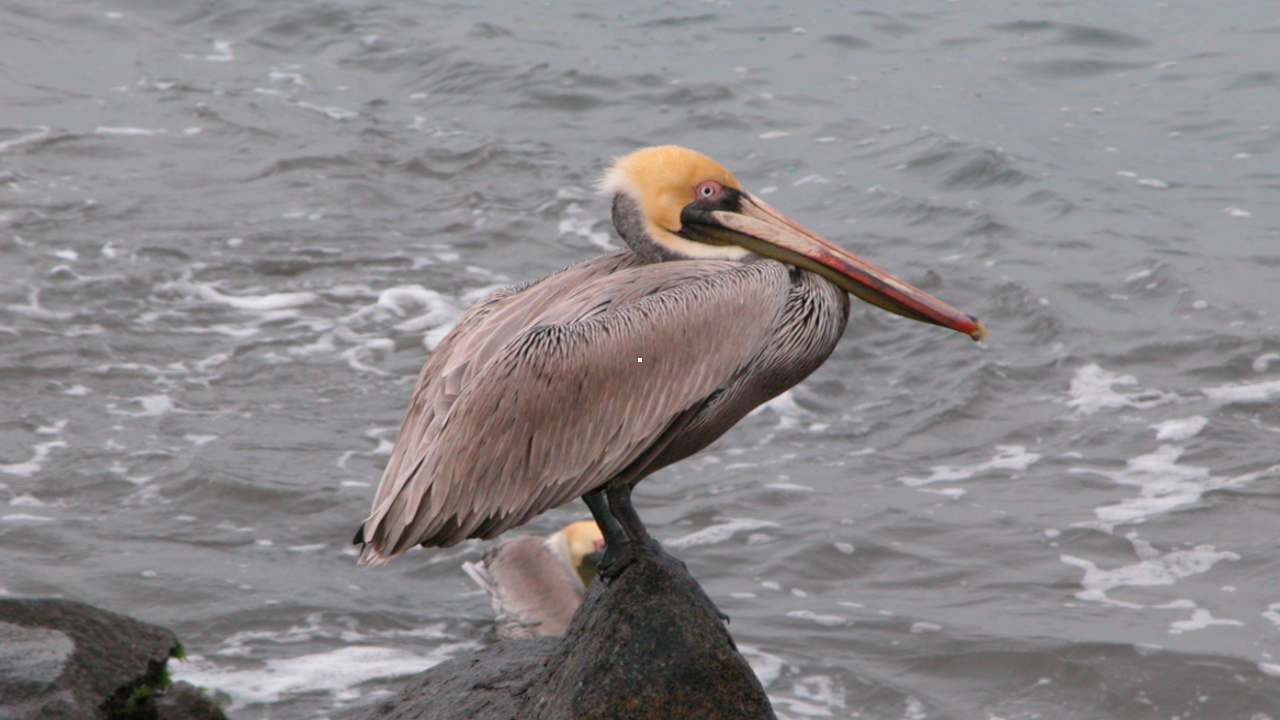

Supplement: Extended Data — Download Extended Data, ZIP file. [file eneuro-11-ENEURO.0417-23.2024-s008.zip › data/images/ImagesAF/Image1.png]

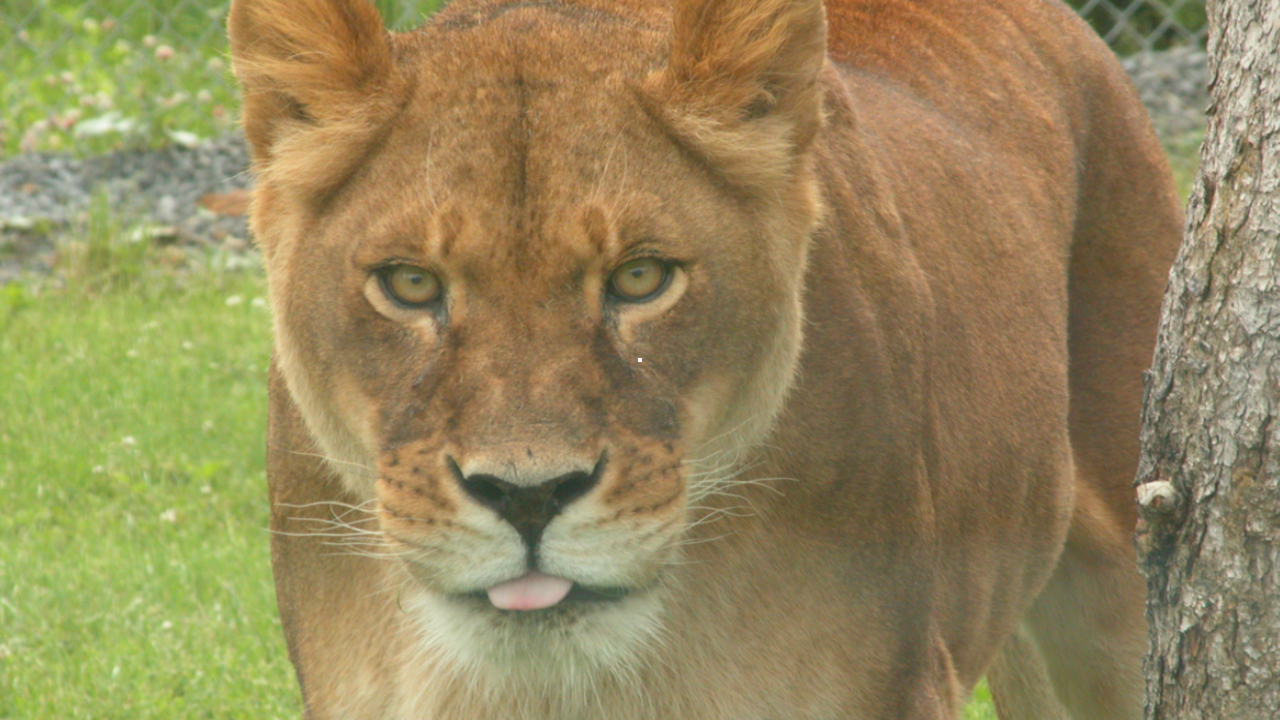

Supplement: Extended Data — Download Extended Data, ZIP file. [file eneuro-11-ENEURO.0417-23.2024-s008.zip › data/images/ImagesAF/Image10.png]

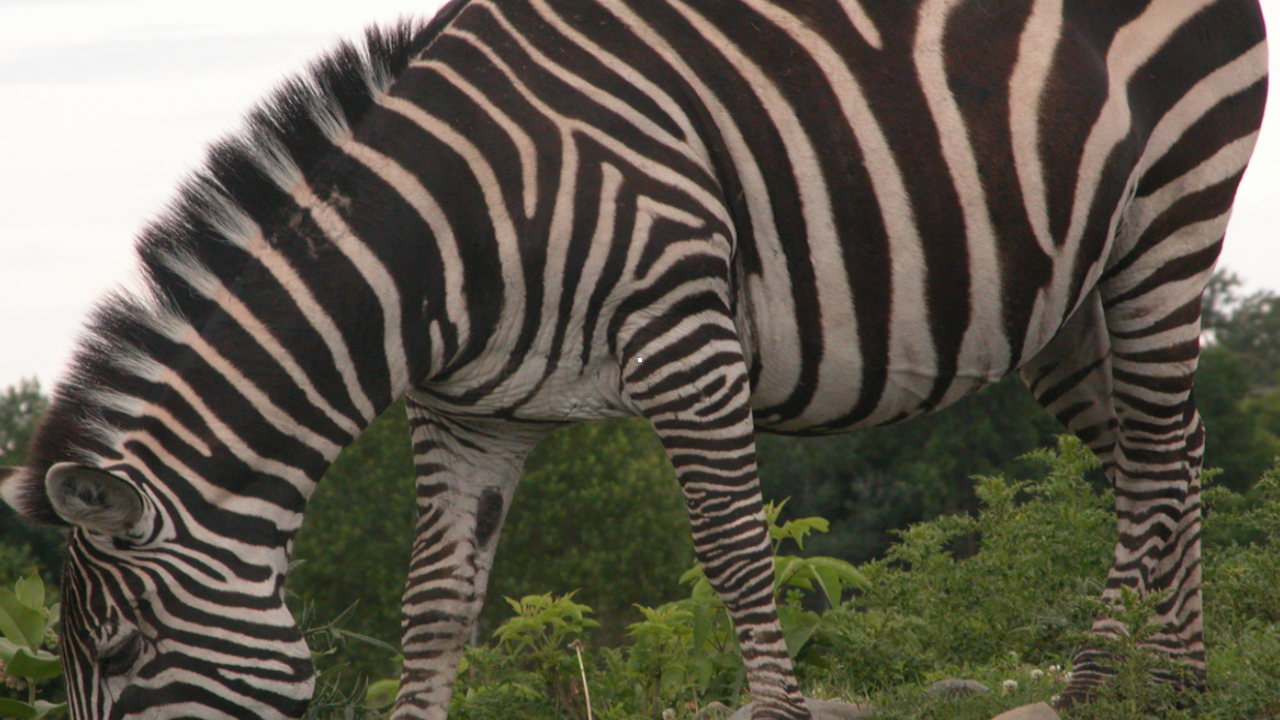

Supplement: Extended Data — Download Extended Data, ZIP file. [file eneuro-11-ENEURO.0417-23.2024-s008.zip › data/images/ImagesAF/Image11.png]

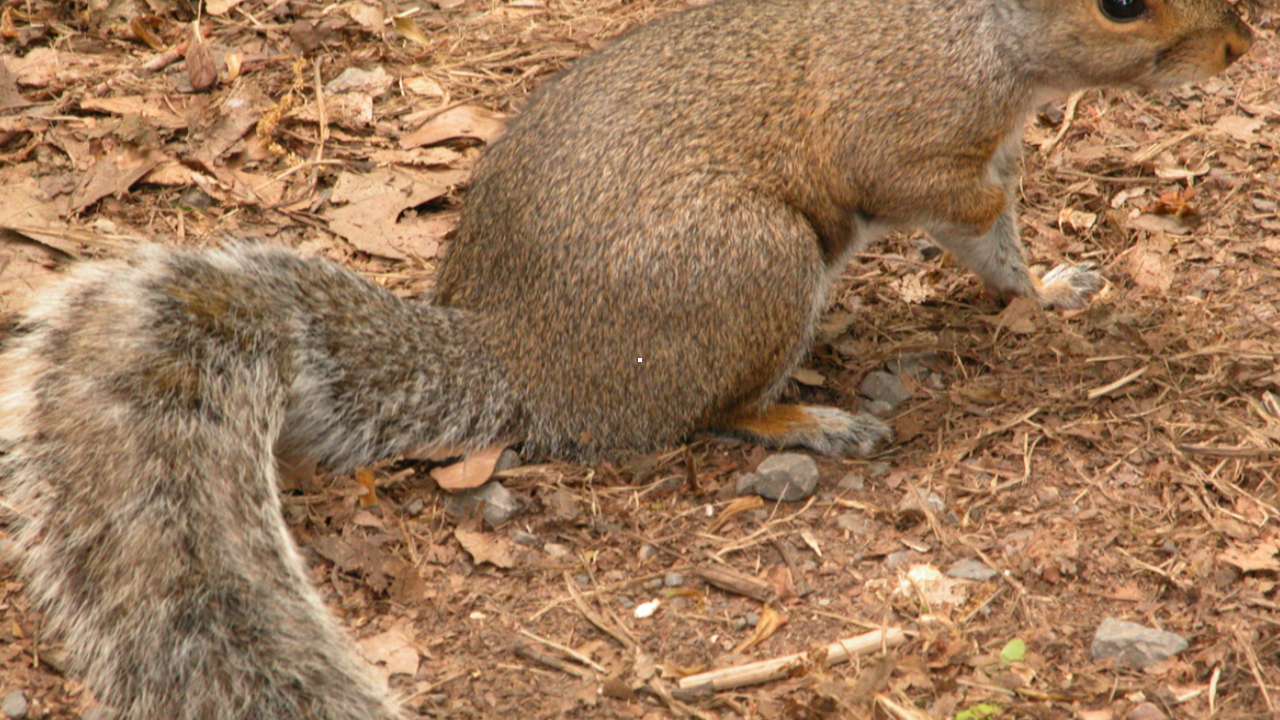

Supplement: Extended Data — Download Extended Data, ZIP file. [file eneuro-11-ENEURO.0417-23.2024-s008.zip › data/images/ImagesAF/Image12.png]

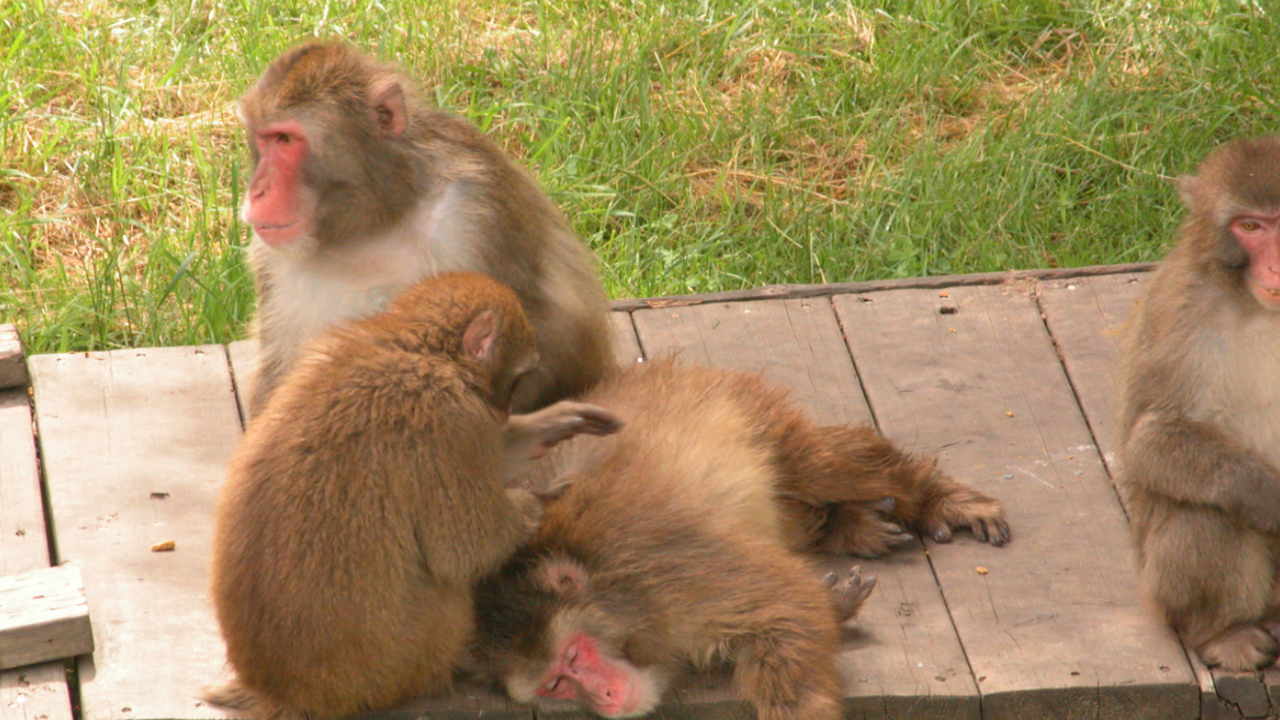

Supplement: Extended Data — Download Extended Data, ZIP file. [file eneuro-11-ENEURO.0417-23.2024-s008.zip › data/images/ImagesAF/Image13.png]

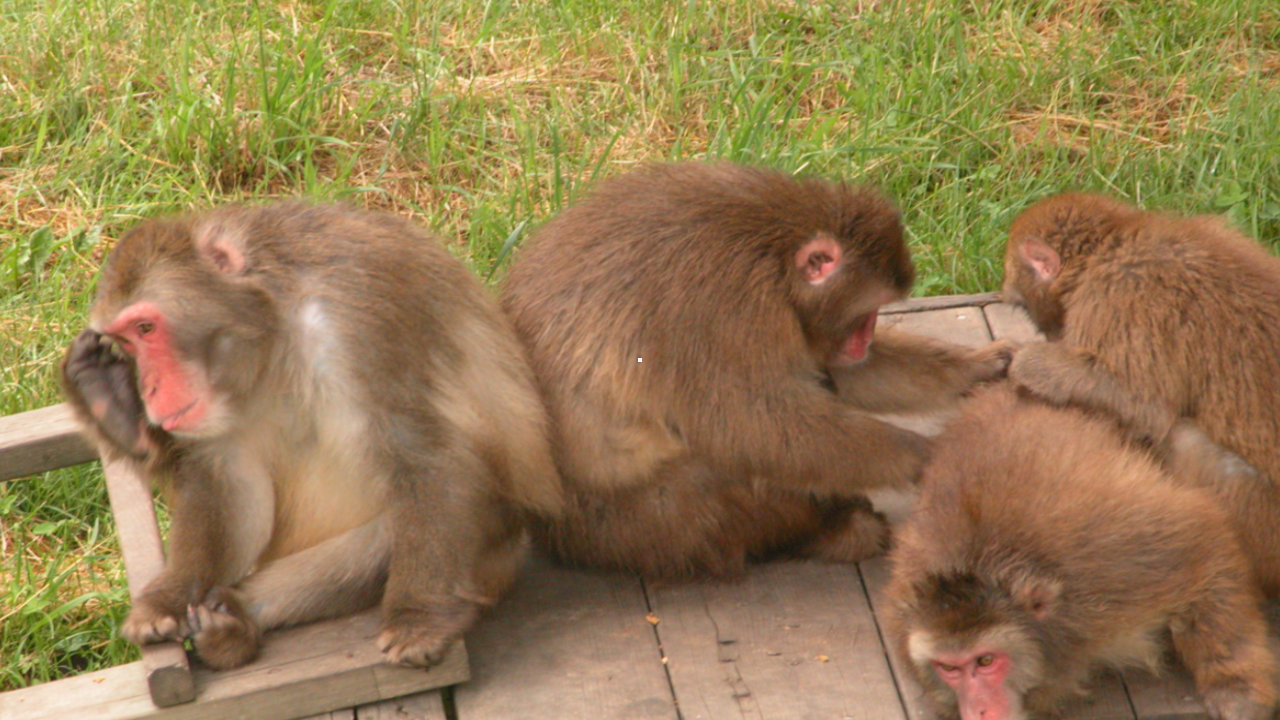

Supplement: Extended Data — Download Extended Data, ZIP file. [file eneuro-11-ENEURO.0417-23.2024-s008.zip › data/images/ImagesAF/Image14.png]

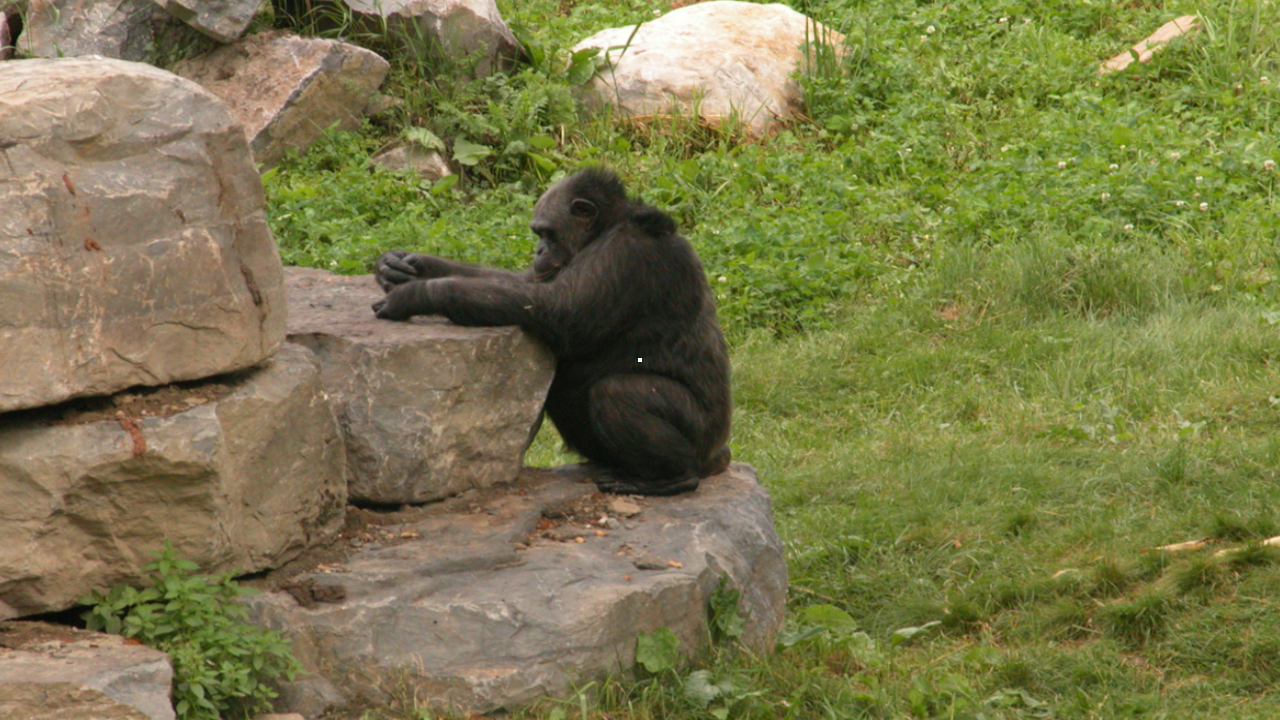

Supplement: Extended Data — Download Extended Data, ZIP file. [file eneuro-11-ENEURO.0417-23.2024-s008.zip › data/images/ImagesAF/Image15.png]

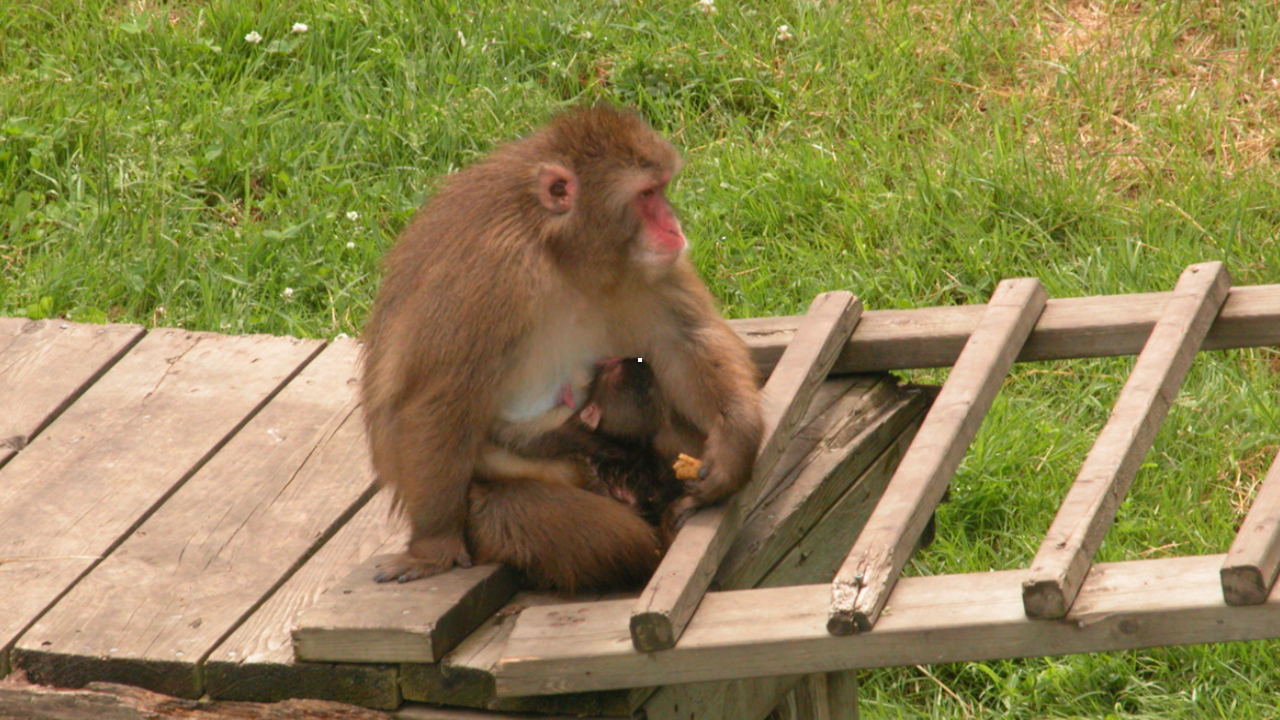

Supplement: Extended Data — Download Extended Data, ZIP file. [file eneuro-11-ENEURO.0417-23.2024-s008.zip › data/images/ImagesAF/Image16.png]

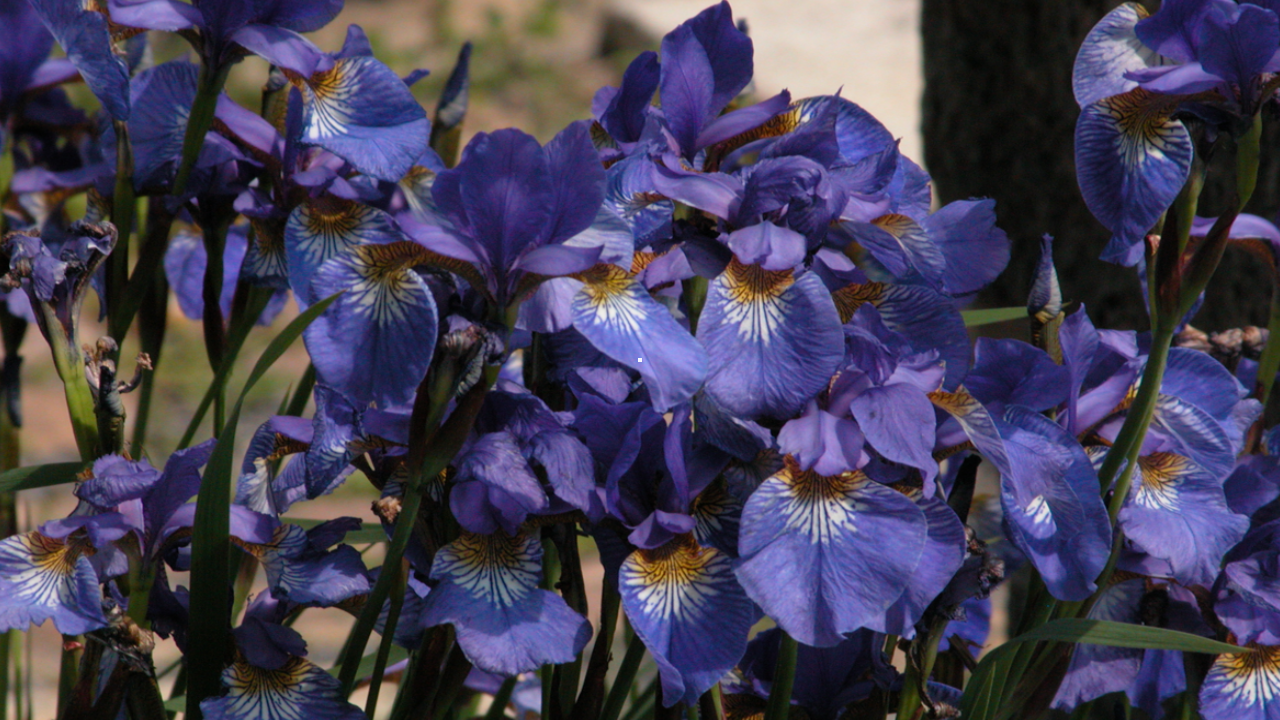

Supplement: Extended Data — Download Extended Data, ZIP file. [file eneuro-11-ENEURO.0417-23.2024-s008.zip › data/images/ImagesAF/Image17.png]

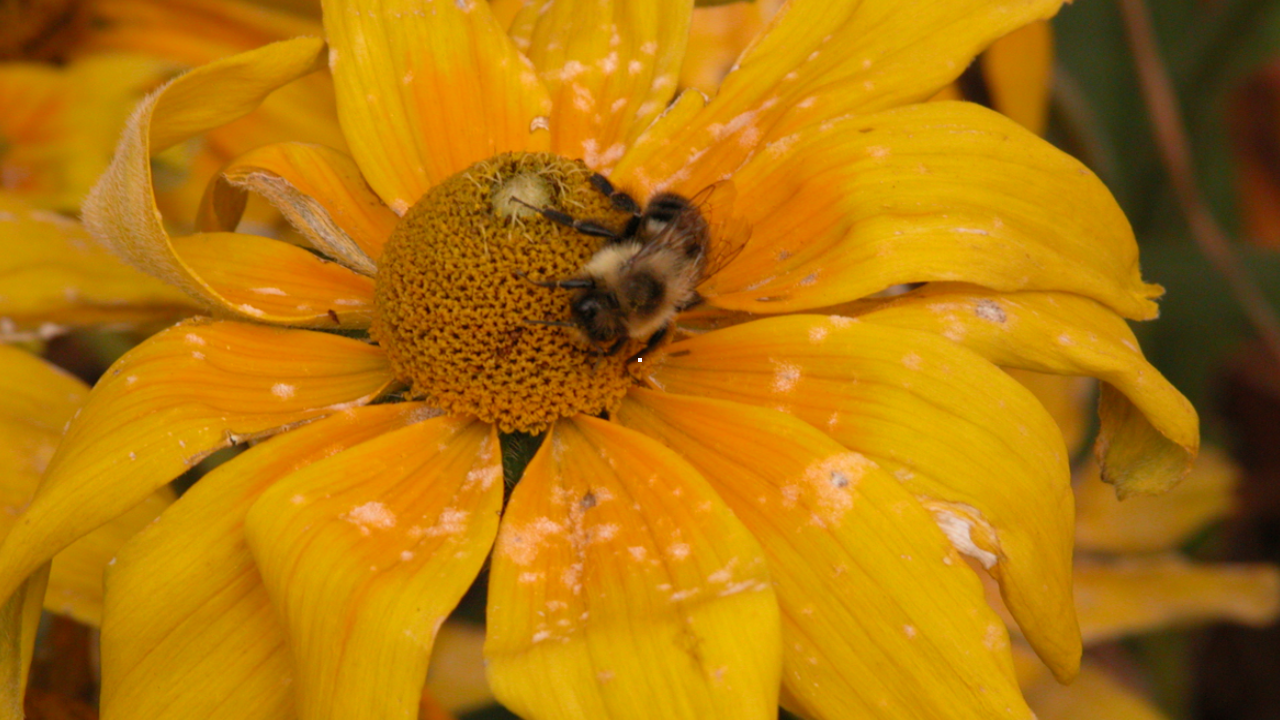

Supplement: Extended Data — Download Extended Data, ZIP file. [file eneuro-11-ENEURO.0417-23.2024-s008.zip › data/images/ImagesAF/Image18.png]

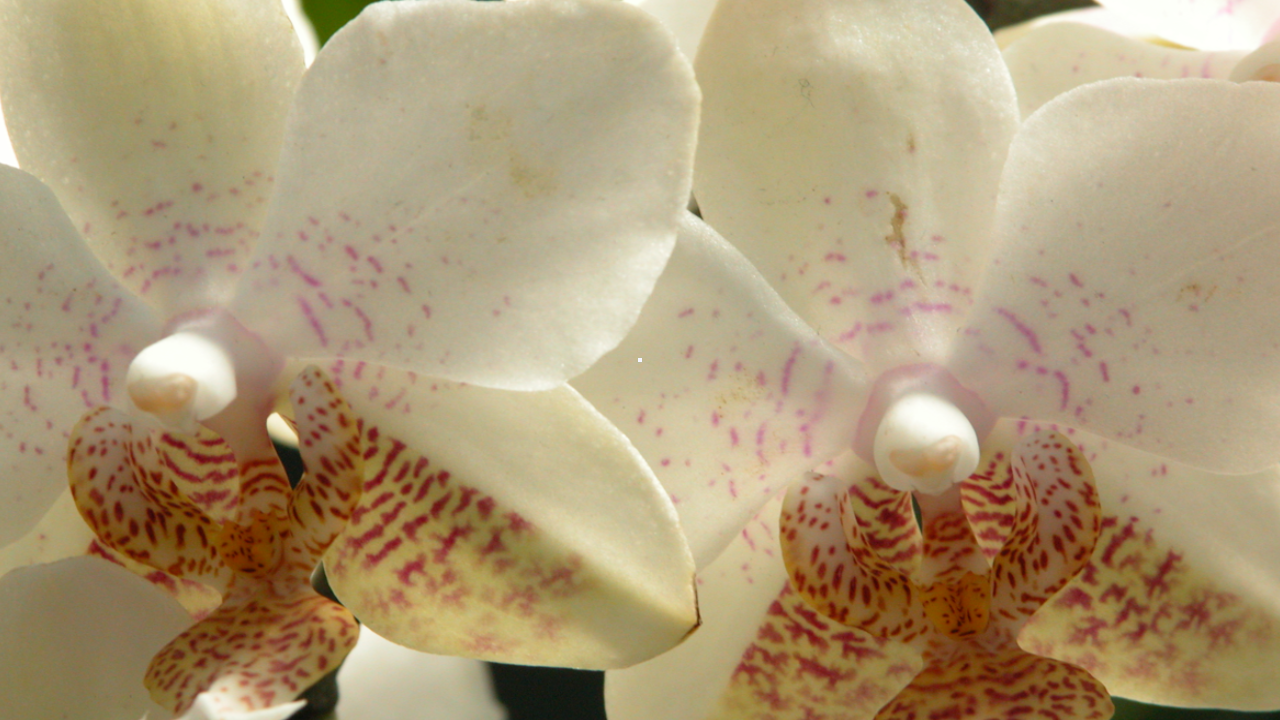

Supplement: Extended Data — Download Extended Data, ZIP file. [file eneuro-11-ENEURO.0417-23.2024-s008.zip › data/images/ImagesAF/Image19.png]

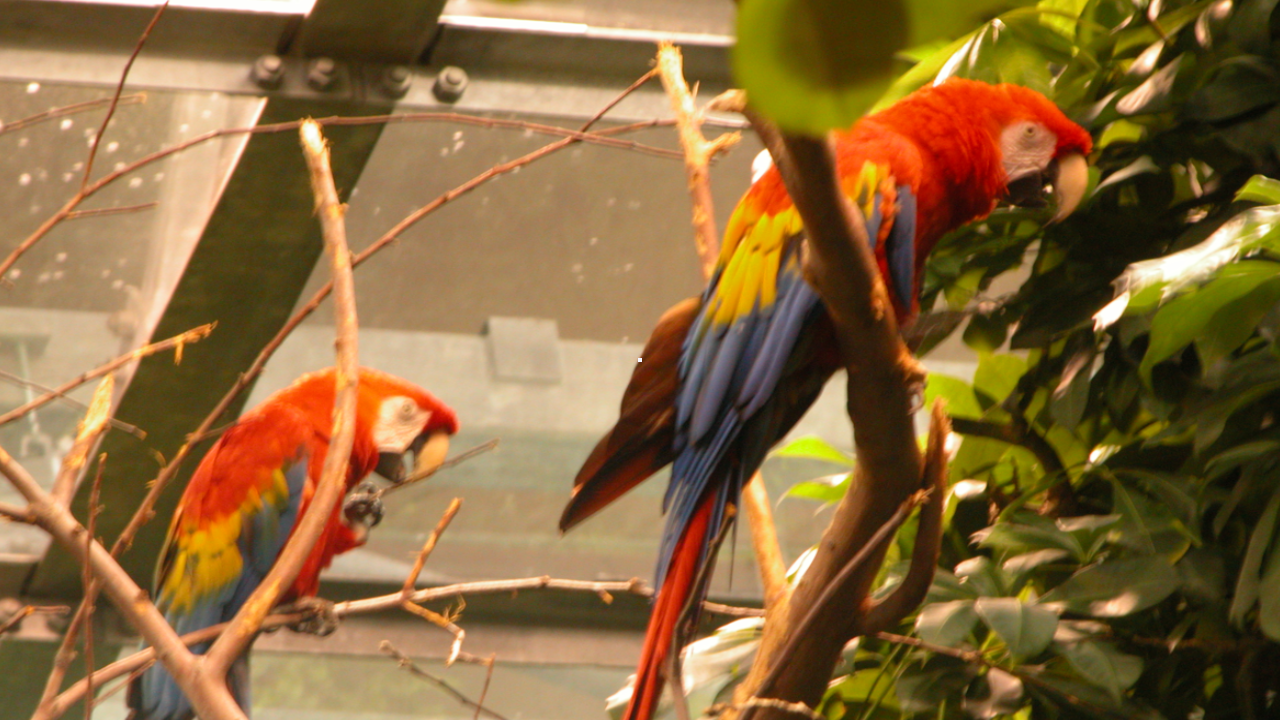

Supplement: Extended Data — Download Extended Data, ZIP file. [file eneuro-11-ENEURO.0417-23.2024-s008.zip › data/images/ImagesAF/Image2.png]

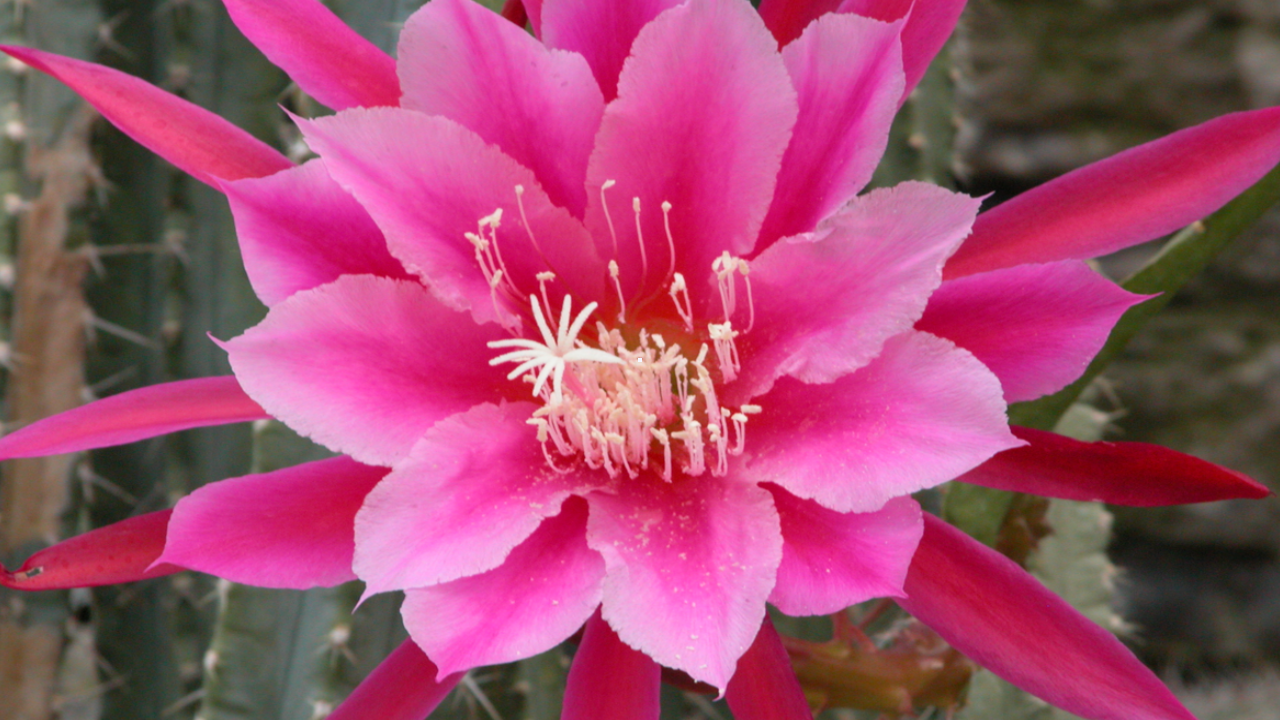

Supplement: Extended Data — Download Extended Data, ZIP file. [file eneuro-11-ENEURO.0417-23.2024-s008.zip › data/images/ImagesAF/Image20.png]

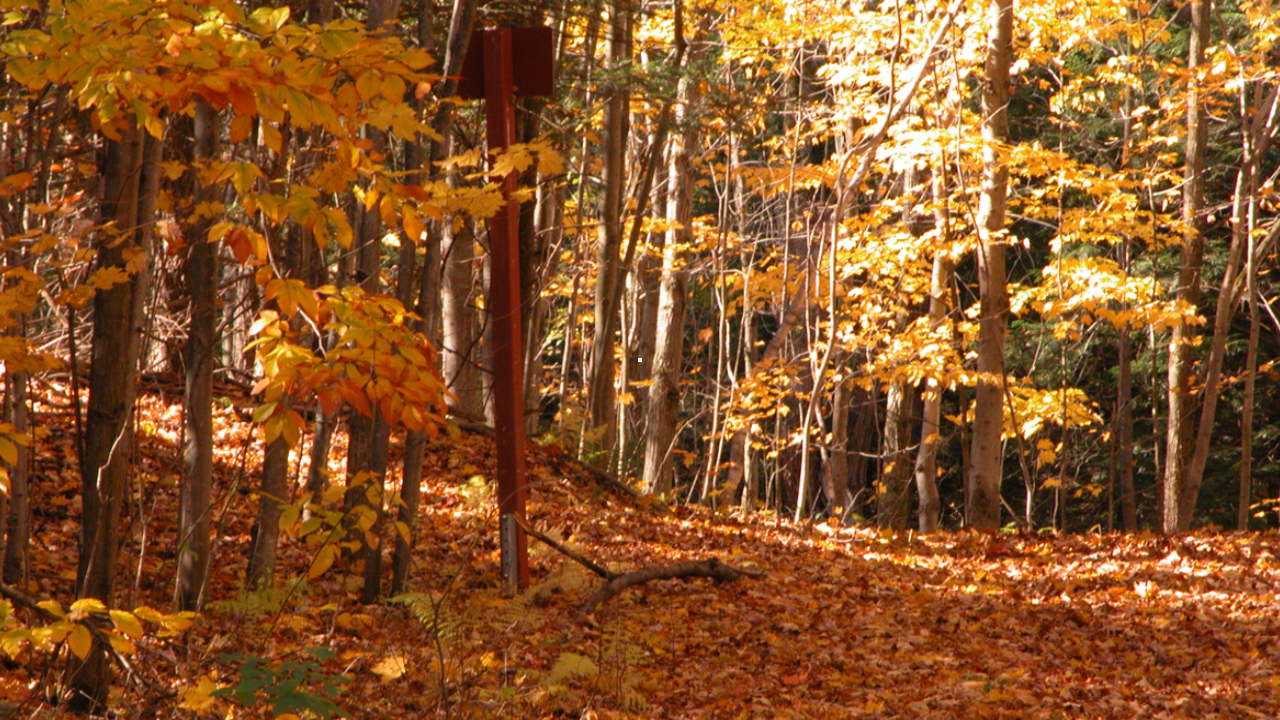

Supplement: Extended Data — Download Extended Data, ZIP file. [file eneuro-11-ENEURO.0417-23.2024-s008.zip › data/images/ImagesAF/Image21.png]

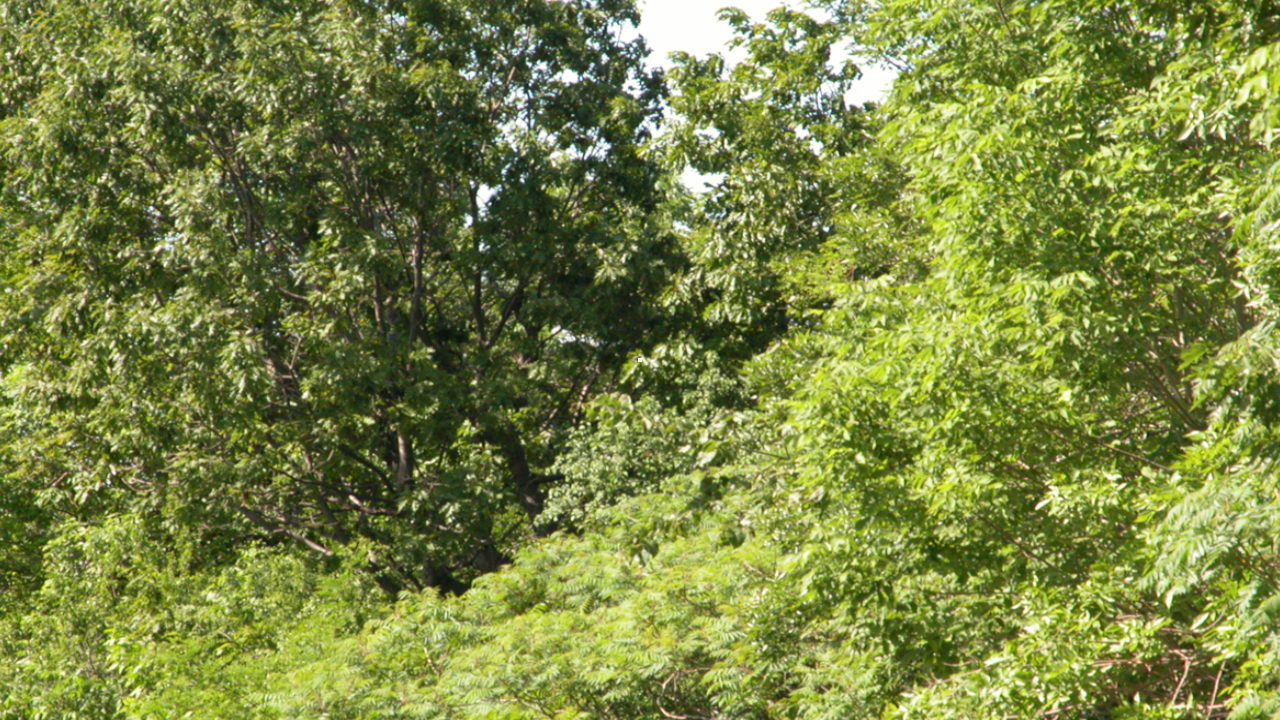

Supplement: Extended Data — Download Extended Data, ZIP file. [file eneuro-11-ENEURO.0417-23.2024-s008.zip › data/images/ImagesAF/Image22.png]

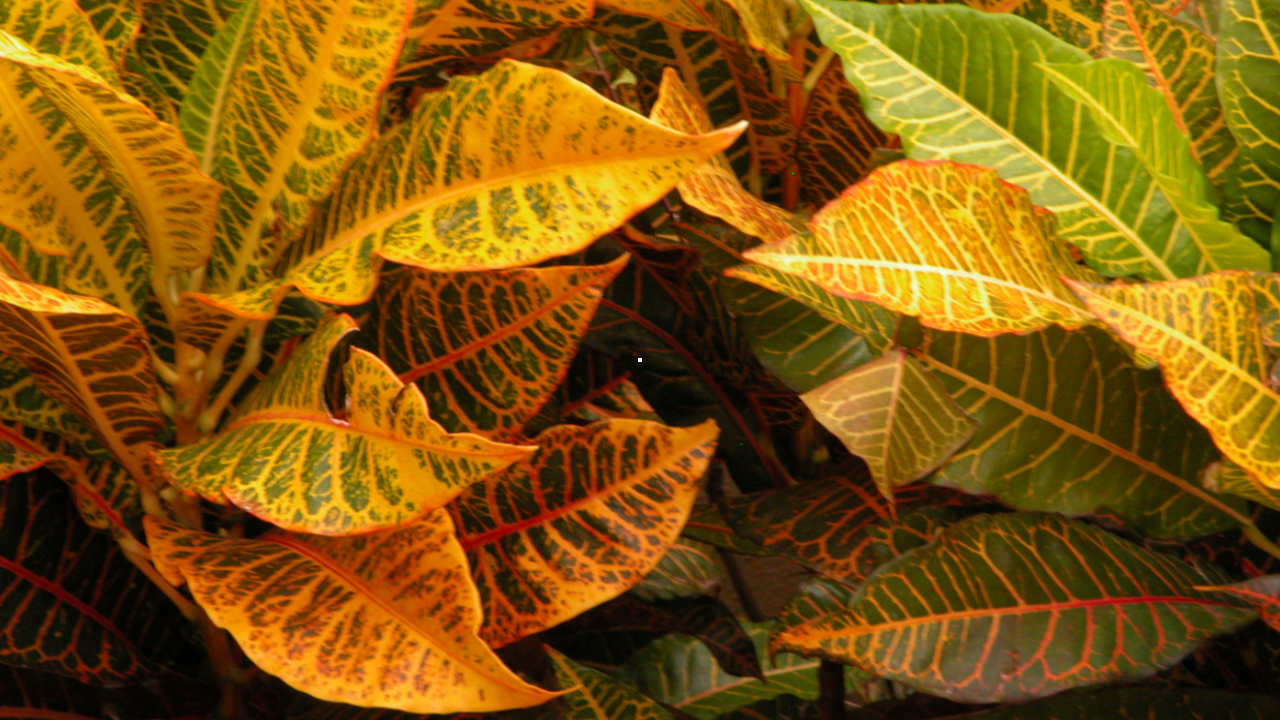

Supplement: Extended Data — Download Extended Data, ZIP file. [file eneuro-11-ENEURO.0417-23.2024-s008.zip › data/images/ImagesAF/Image23.png]

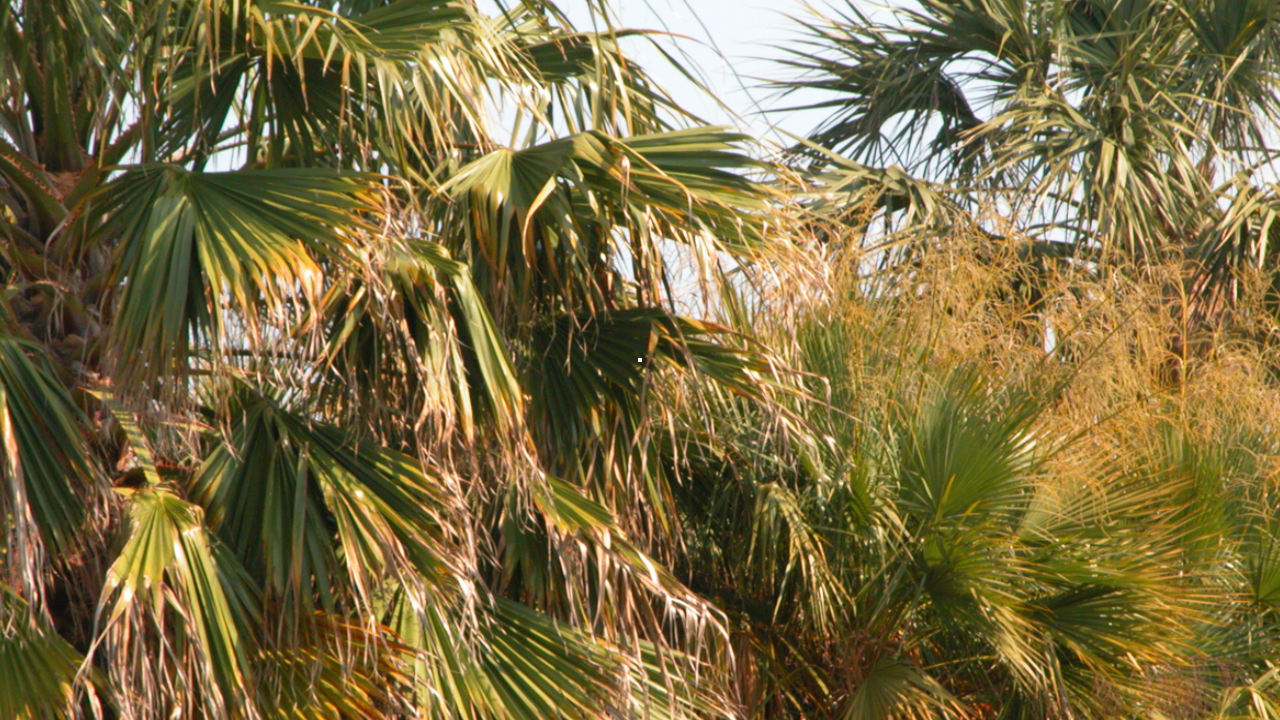

Supplement: Extended Data — Download Extended Data, ZIP file. [file eneuro-11-ENEURO.0417-23.2024-s008.zip › data/images/ImagesAF/Image24.png]

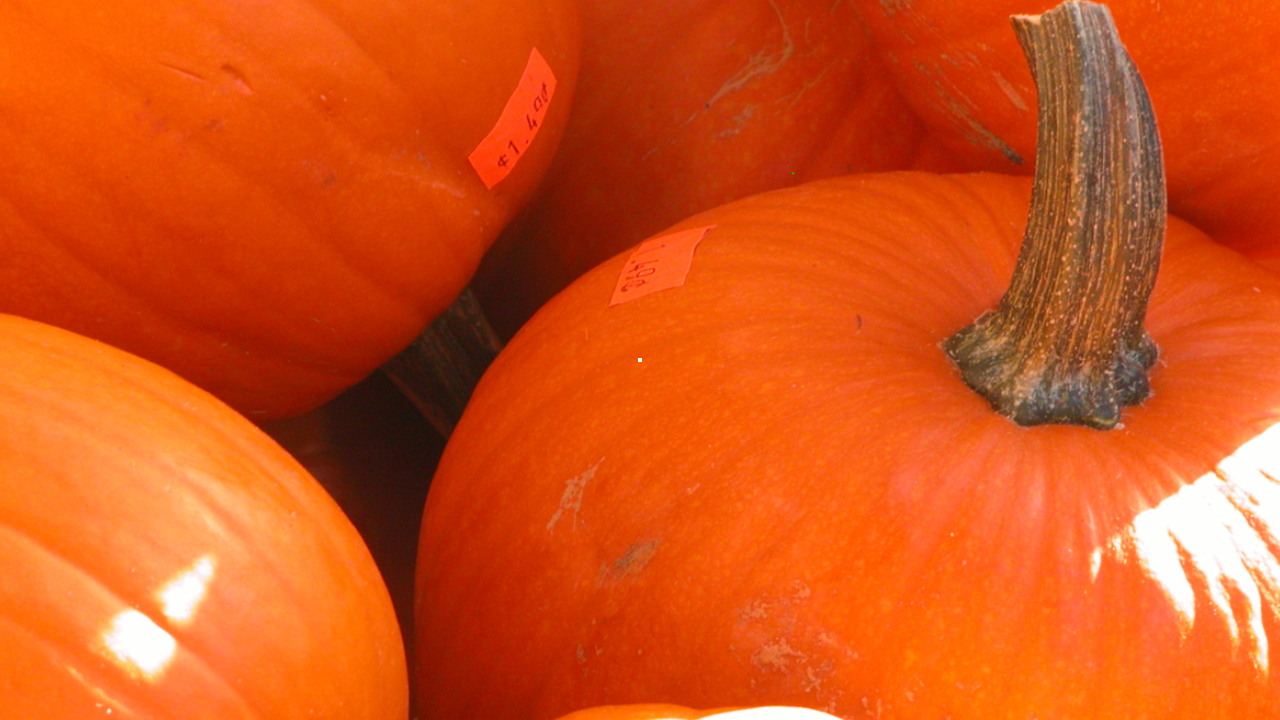

Supplement: Extended Data — Download Extended Data, ZIP file. [file eneuro-11-ENEURO.0417-23.2024-s008.zip › data/images/ImagesAF/Image25.png]

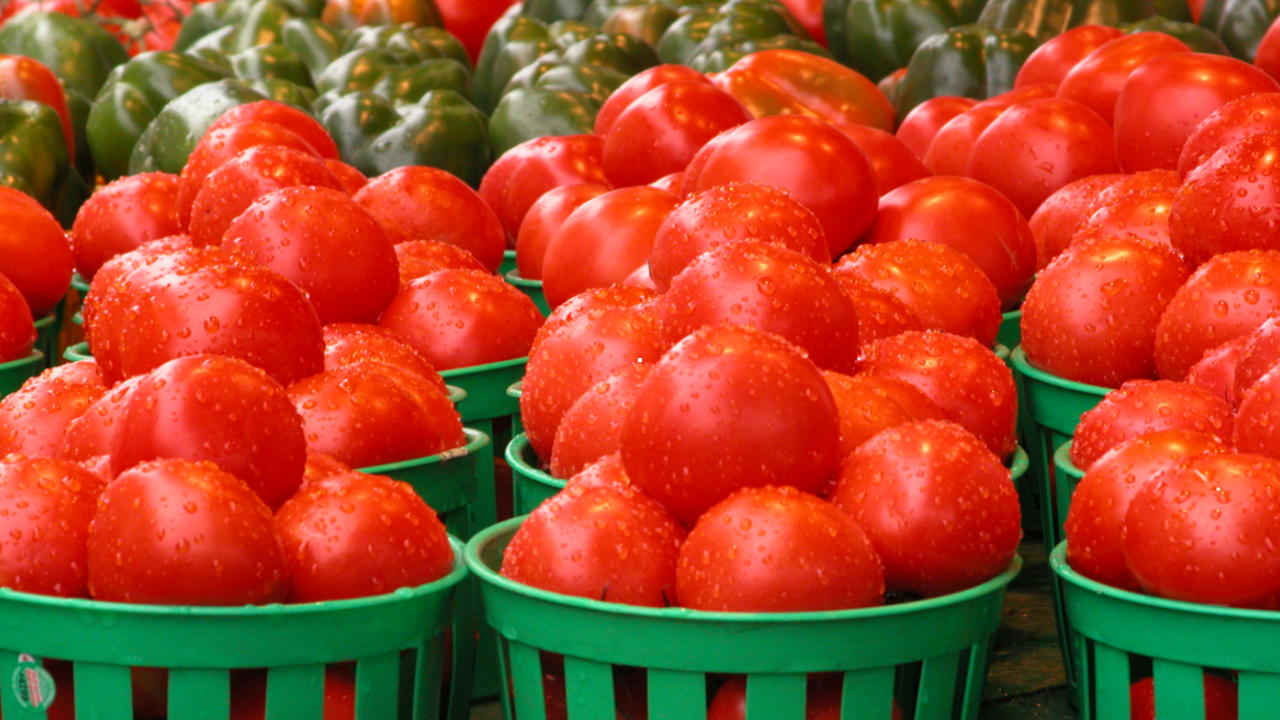

Supplement: Extended Data — Download Extended Data, ZIP file. [file eneuro-11-ENEURO.0417-23.2024-s008.zip › data/images/ImagesAF/Image26.png]

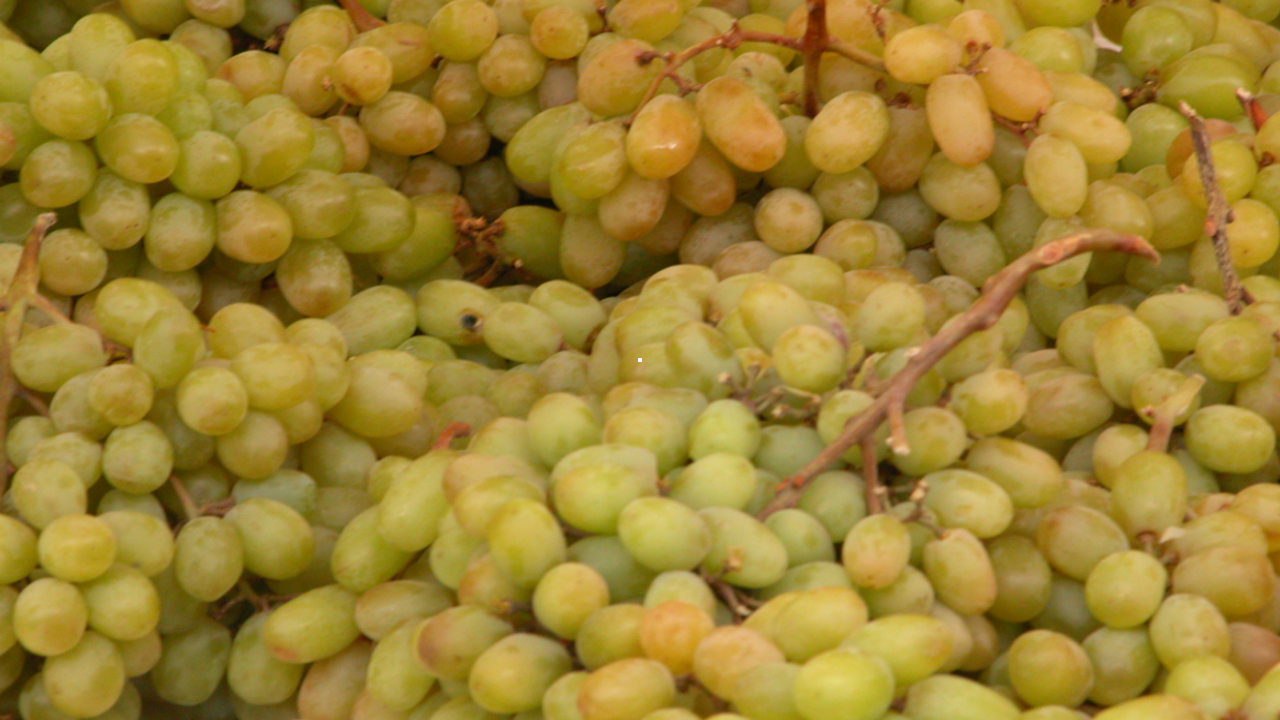

Supplement: Extended Data — Download Extended Data, ZIP file. [file eneuro-11-ENEURO.0417-23.2024-s008.zip › data/images/ImagesAF/Image27.png]

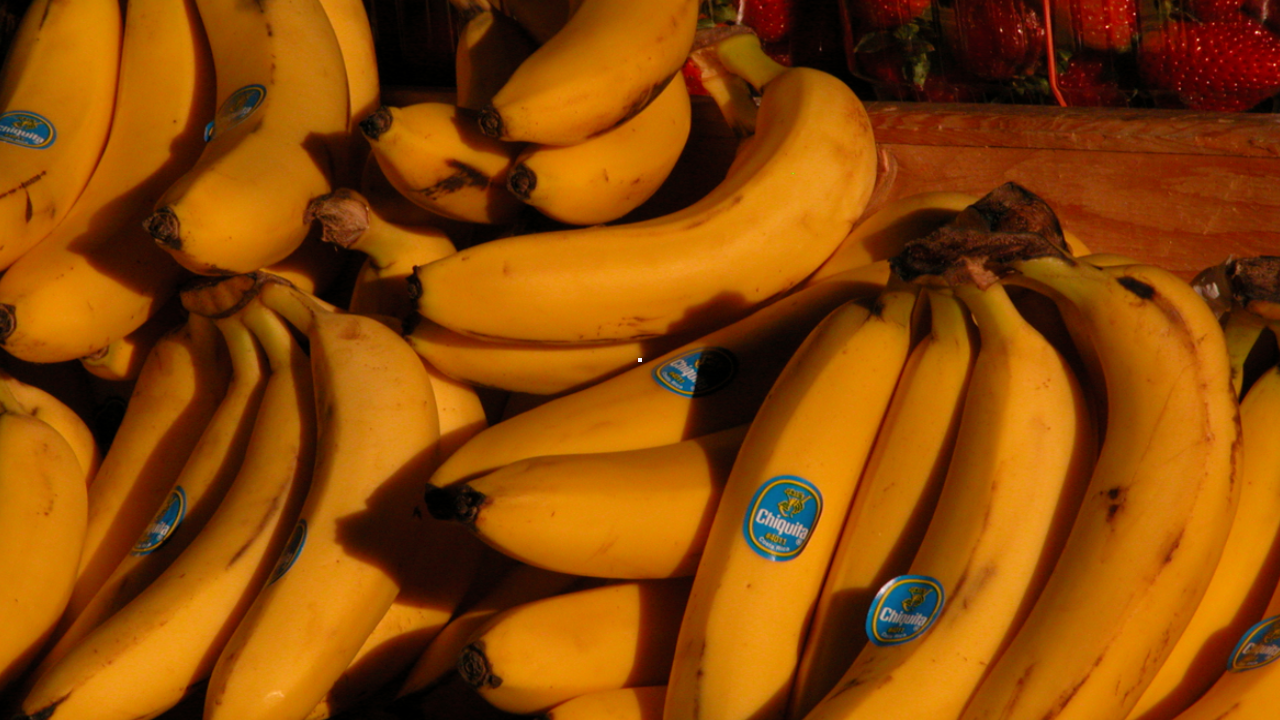

Supplement: Extended Data — Download Extended Data, ZIP file. [file eneuro-11-ENEURO.0417-23.2024-s008.zip › data/images/ImagesAF/Image28.png]

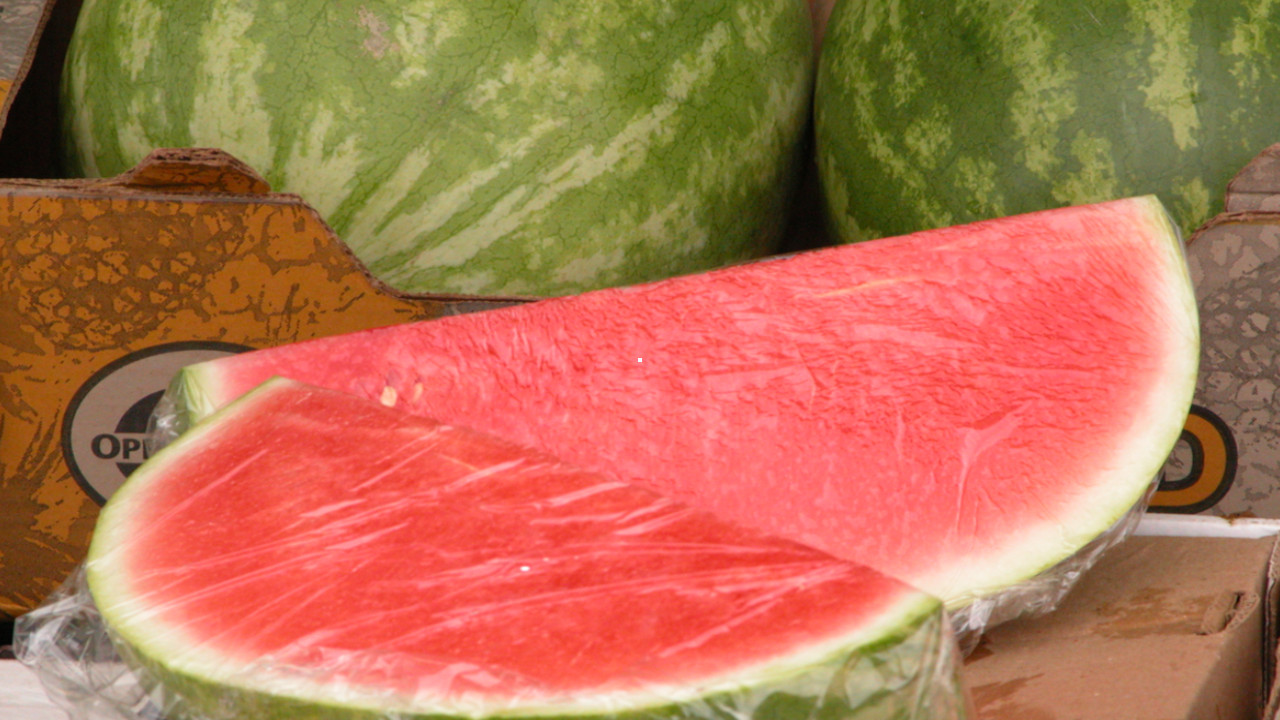

Supplement: Extended Data — Download Extended Data, ZIP file. [file eneuro-11-ENEURO.0417-23.2024-s008.zip › data/images/ImagesAF/Image29.png]

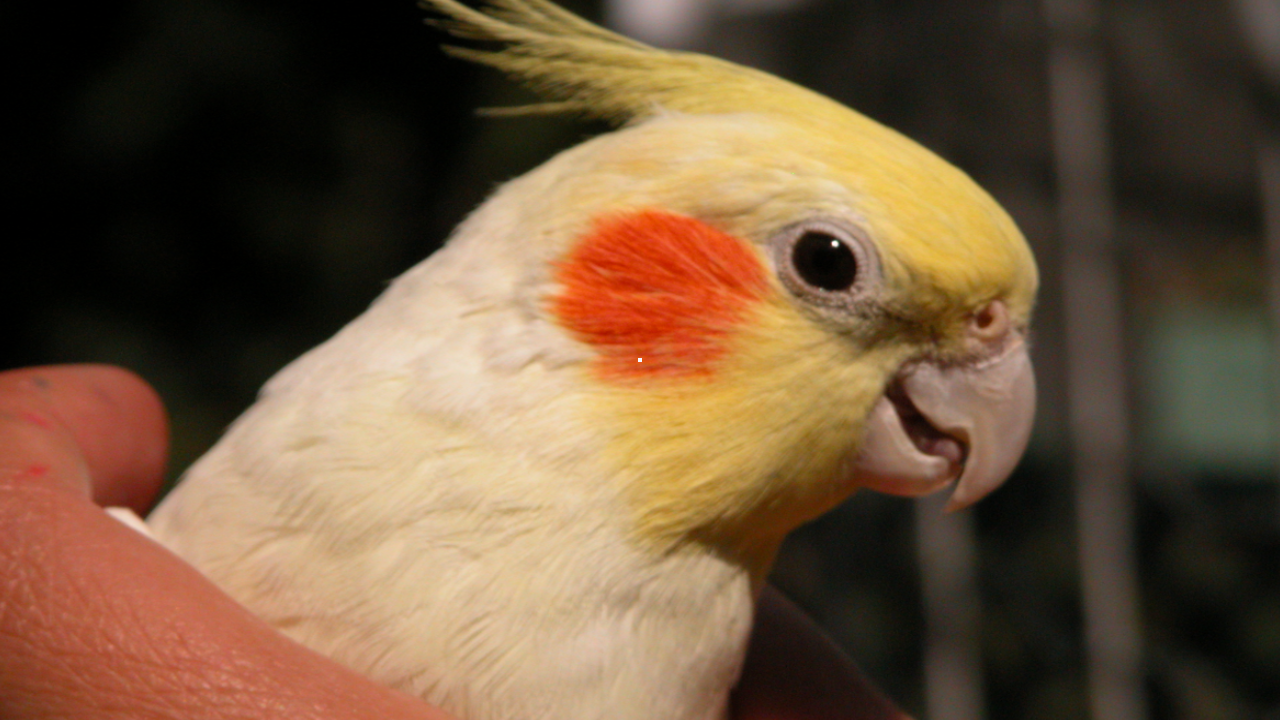

Supplement: Extended Data — Download Extended Data, ZIP file. [file eneuro-11-ENEURO.0417-23.2024-s008.zip › data/images/ImagesAF/Image3.png]

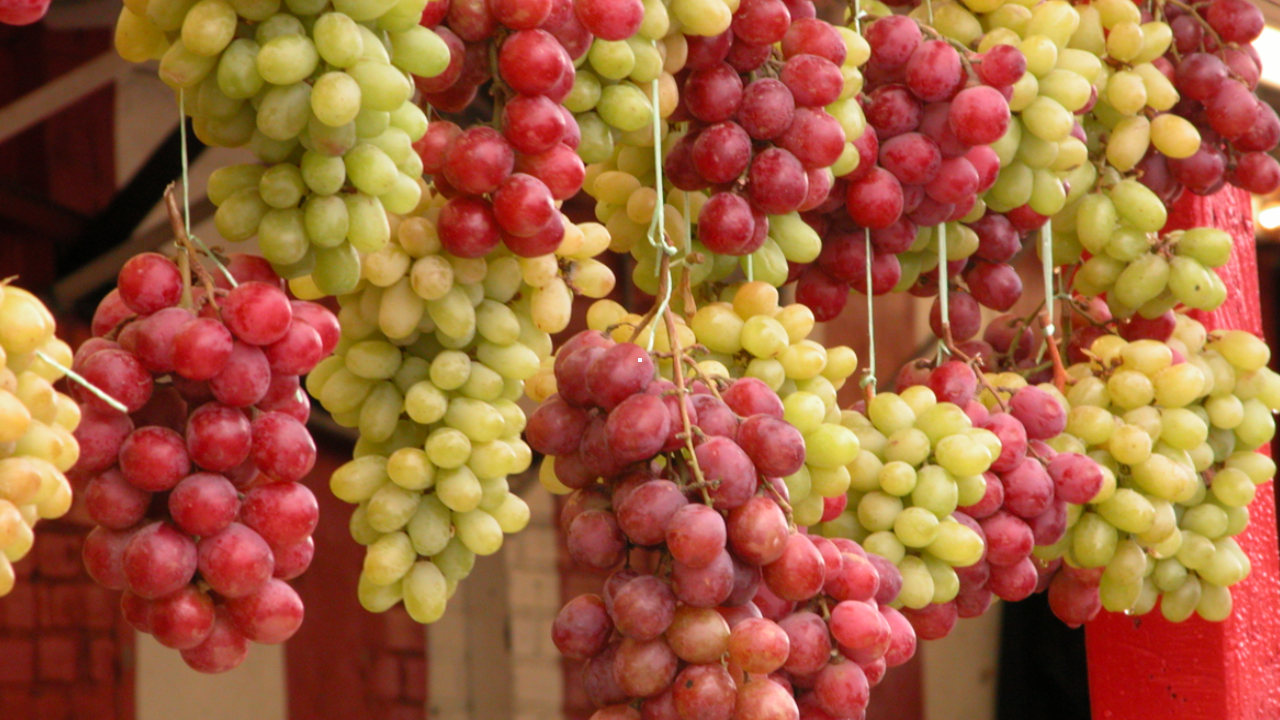

Supplement: Extended Data — Download Extended Data, ZIP file. [file eneuro-11-ENEURO.0417-23.2024-s008.zip › data/images/ImagesAF/Image30.png]

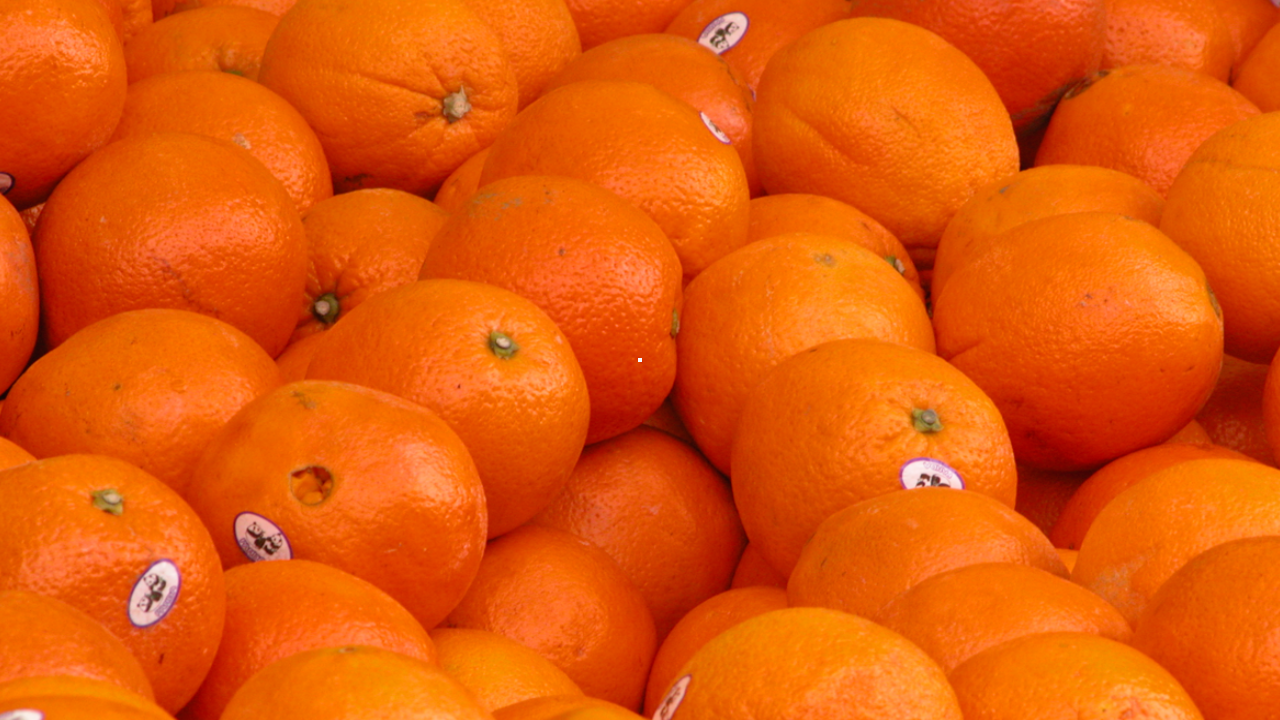

Supplement: Extended Data — Download Extended Data, ZIP file. [file eneuro-11-ENEURO.0417-23.2024-s008.zip › data/images/ImagesAF/Image31.png]

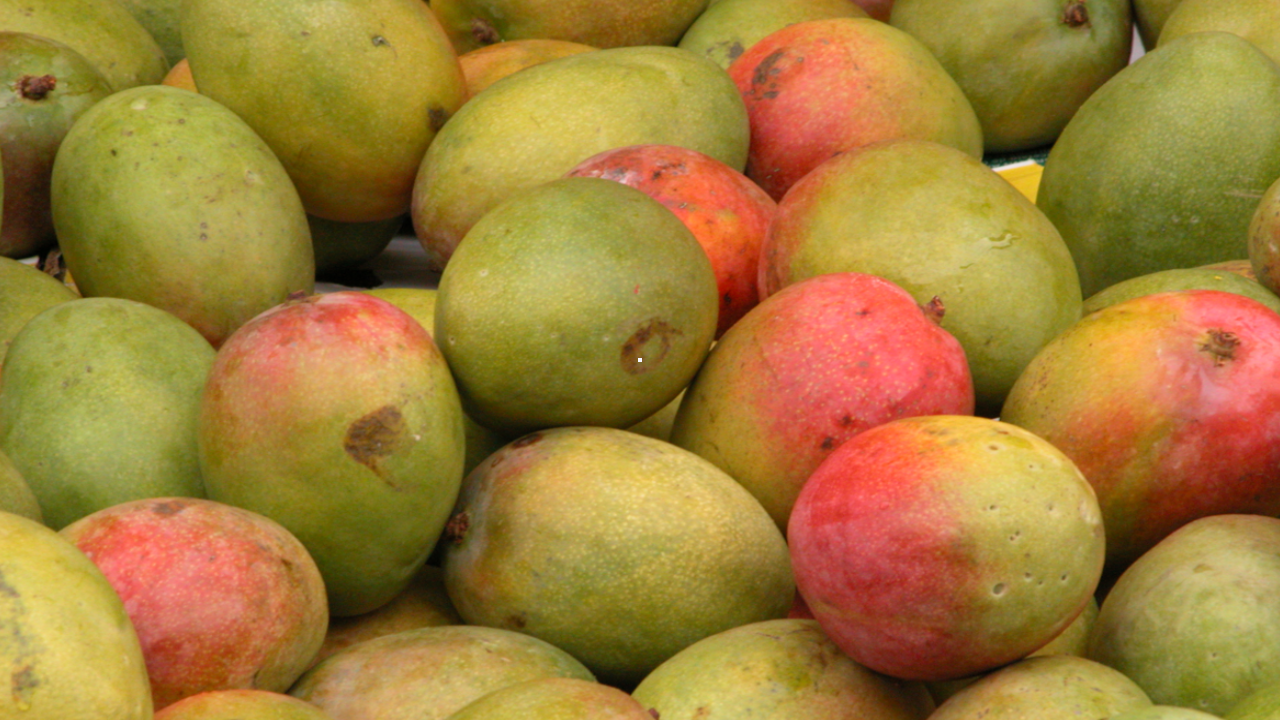

Supplement: Extended Data — Download Extended Data, ZIP file. [file eneuro-11-ENEURO.0417-23.2024-s008.zip › data/images/ImagesAF/Image32.png]

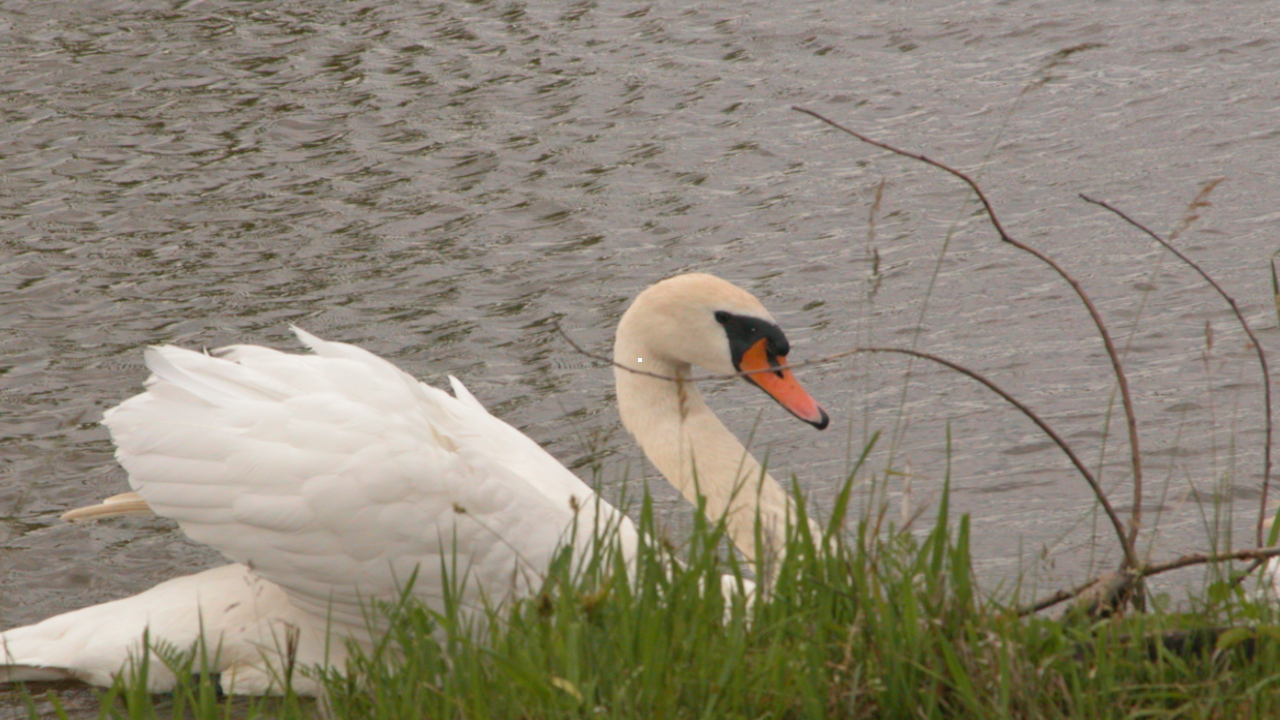

Supplement: Extended Data — Download Extended Data, ZIP file. [file eneuro-11-ENEURO.0417-23.2024-s008.zip › data/images/ImagesAF/Image4.png]

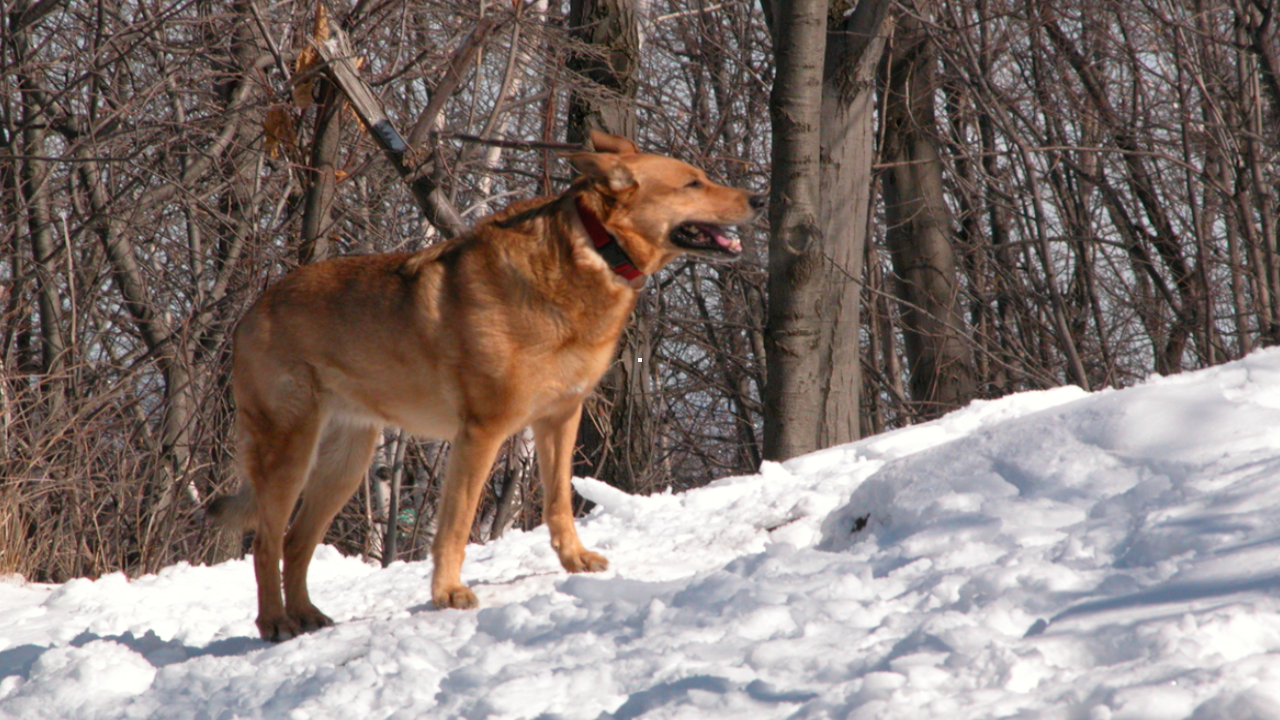

Supplement: Extended Data — Download Extended Data, ZIP file. [file eneuro-11-ENEURO.0417-23.2024-s008.zip › data/images/ImagesAF/Image5.png]

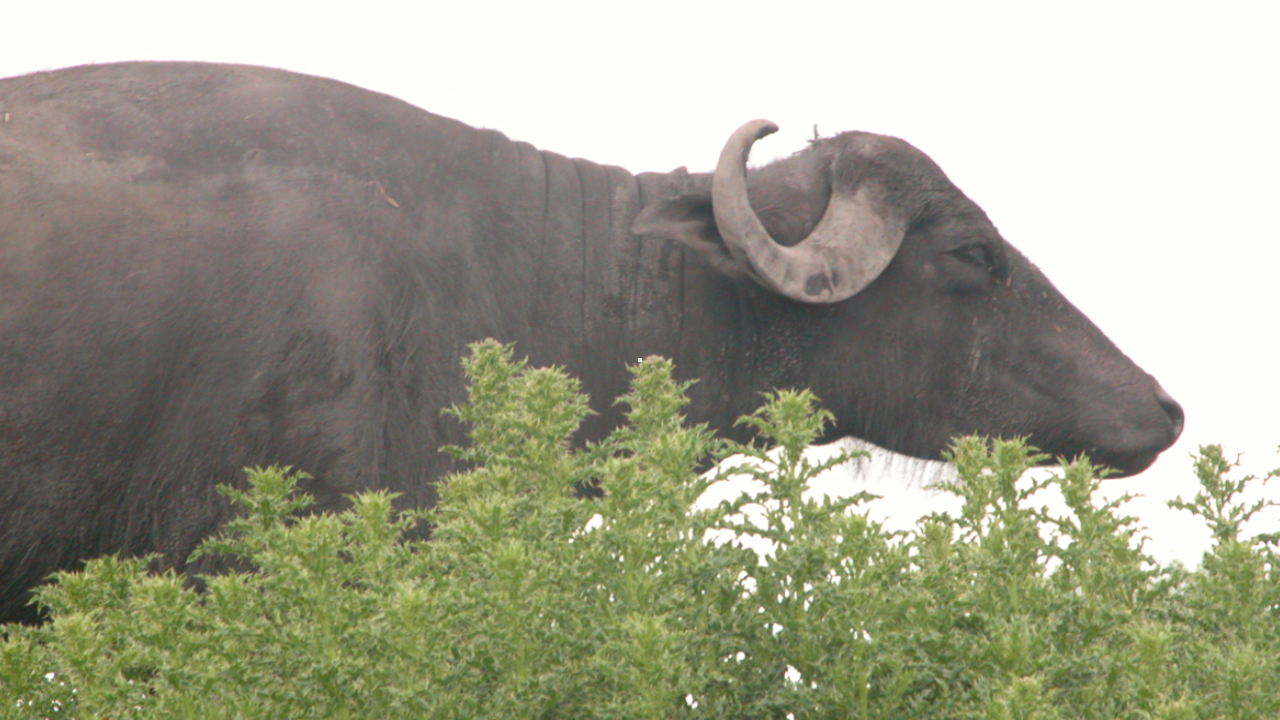

Supplement: Extended Data — Download Extended Data, ZIP file. [file eneuro-11-ENEURO.0417-23.2024-s008.zip › data/images/ImagesAF/Image6.png]

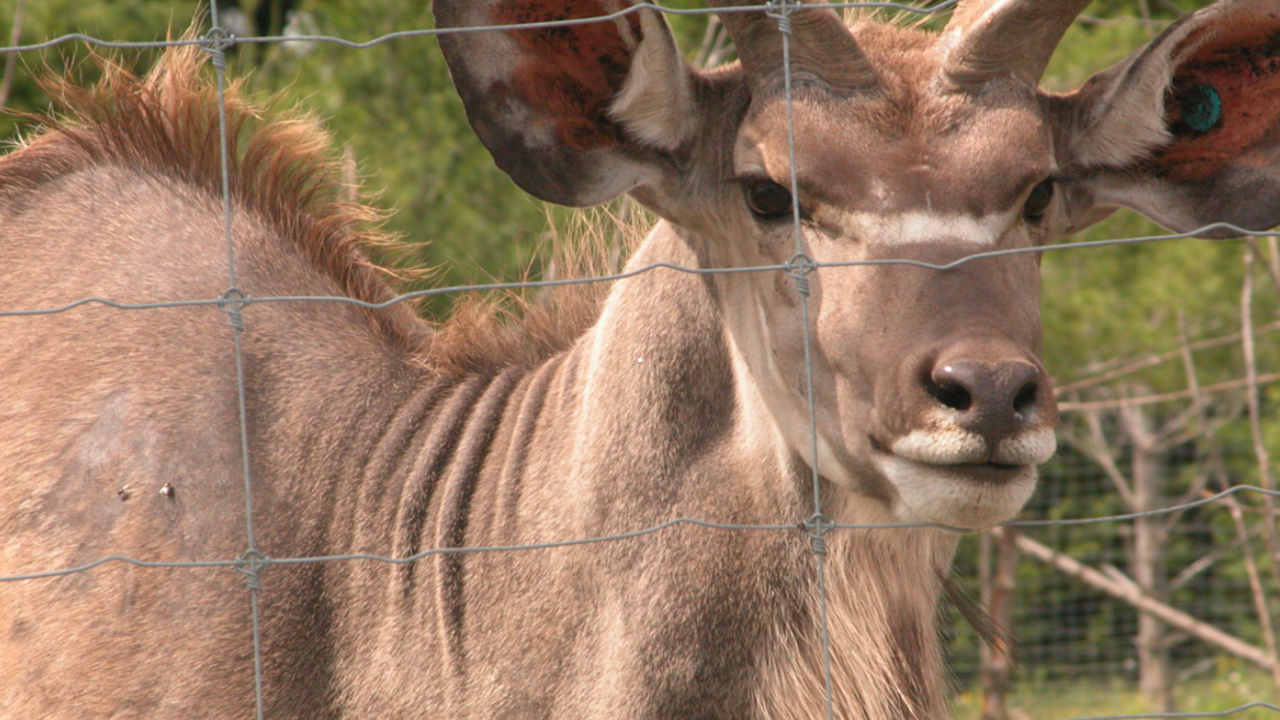

Supplement: Extended Data — Download Extended Data, ZIP file. [file eneuro-11-ENEURO.0417-23.2024-s008.zip › data/images/ImagesAF/Image7.png]

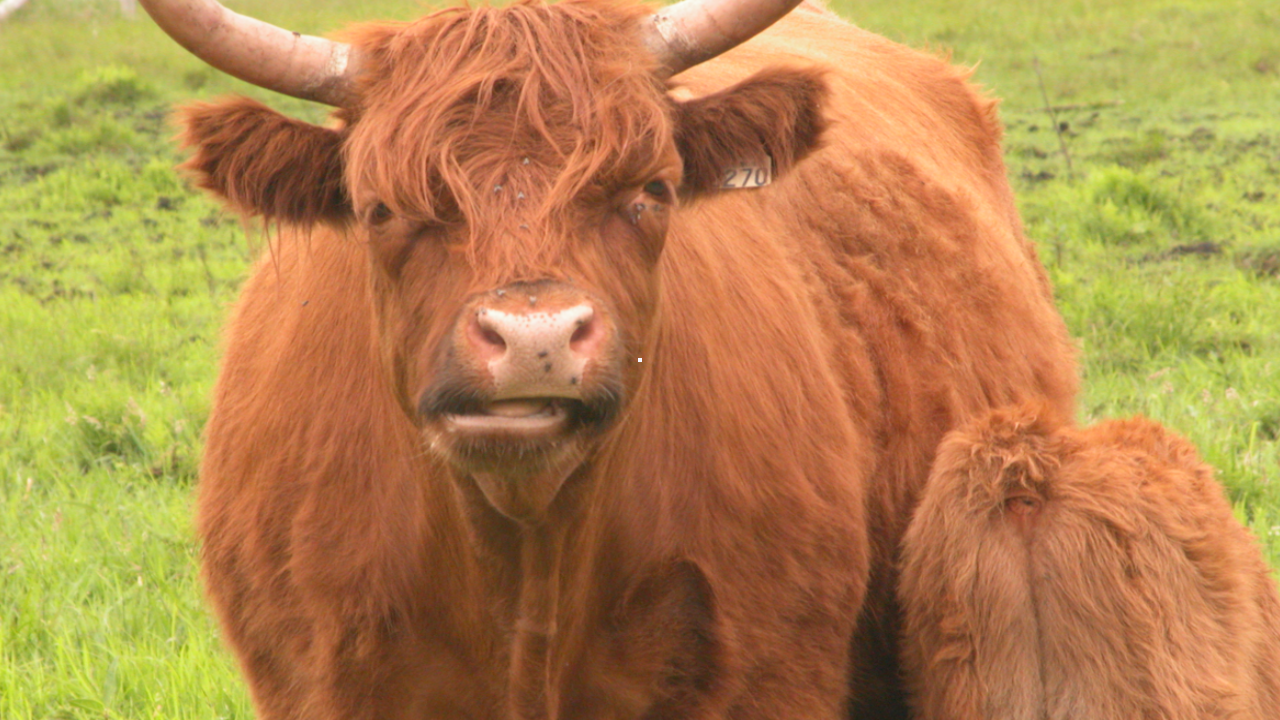

Supplement: Extended Data — Download Extended Data, ZIP file. [file eneuro-11-ENEURO.0417-23.2024-s008.zip › data/images/ImagesAF/Image8.png]

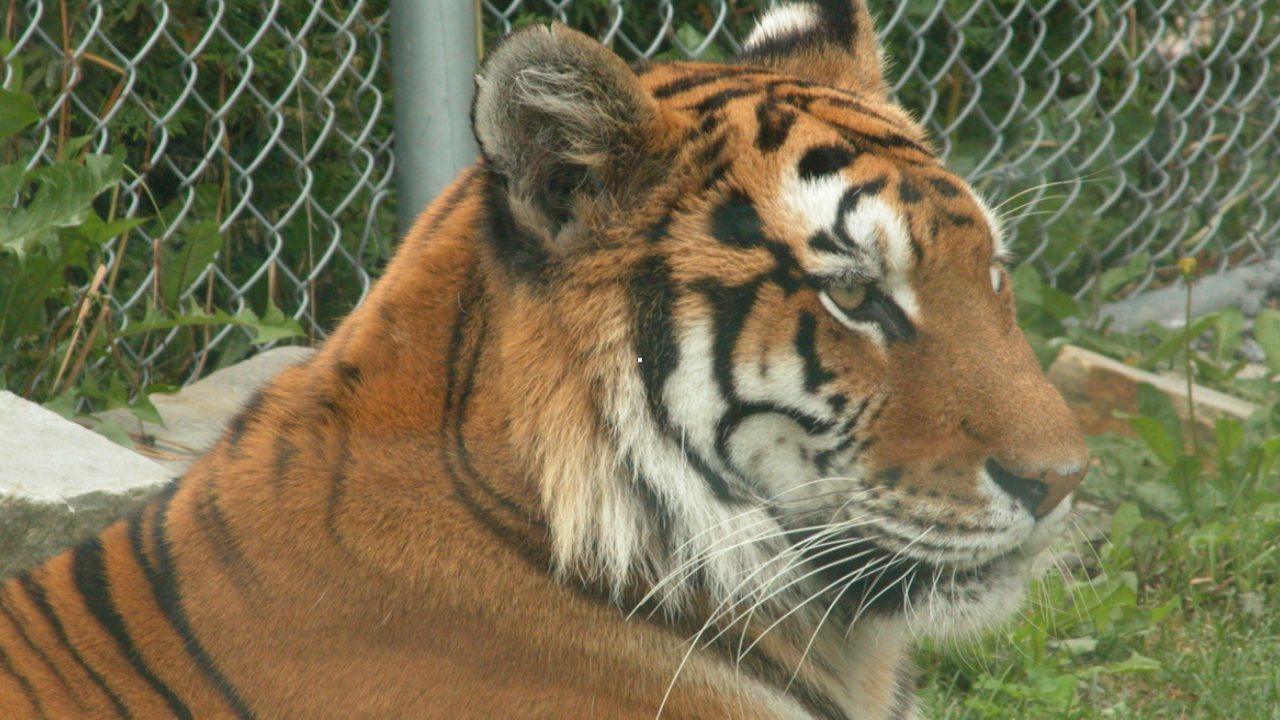

Supplement: Extended Data — Download Extended Data, ZIP file. [file eneuro-11-ENEURO.0417-23.2024-s008.zip › data/images/ImagesAF/Image9.png]

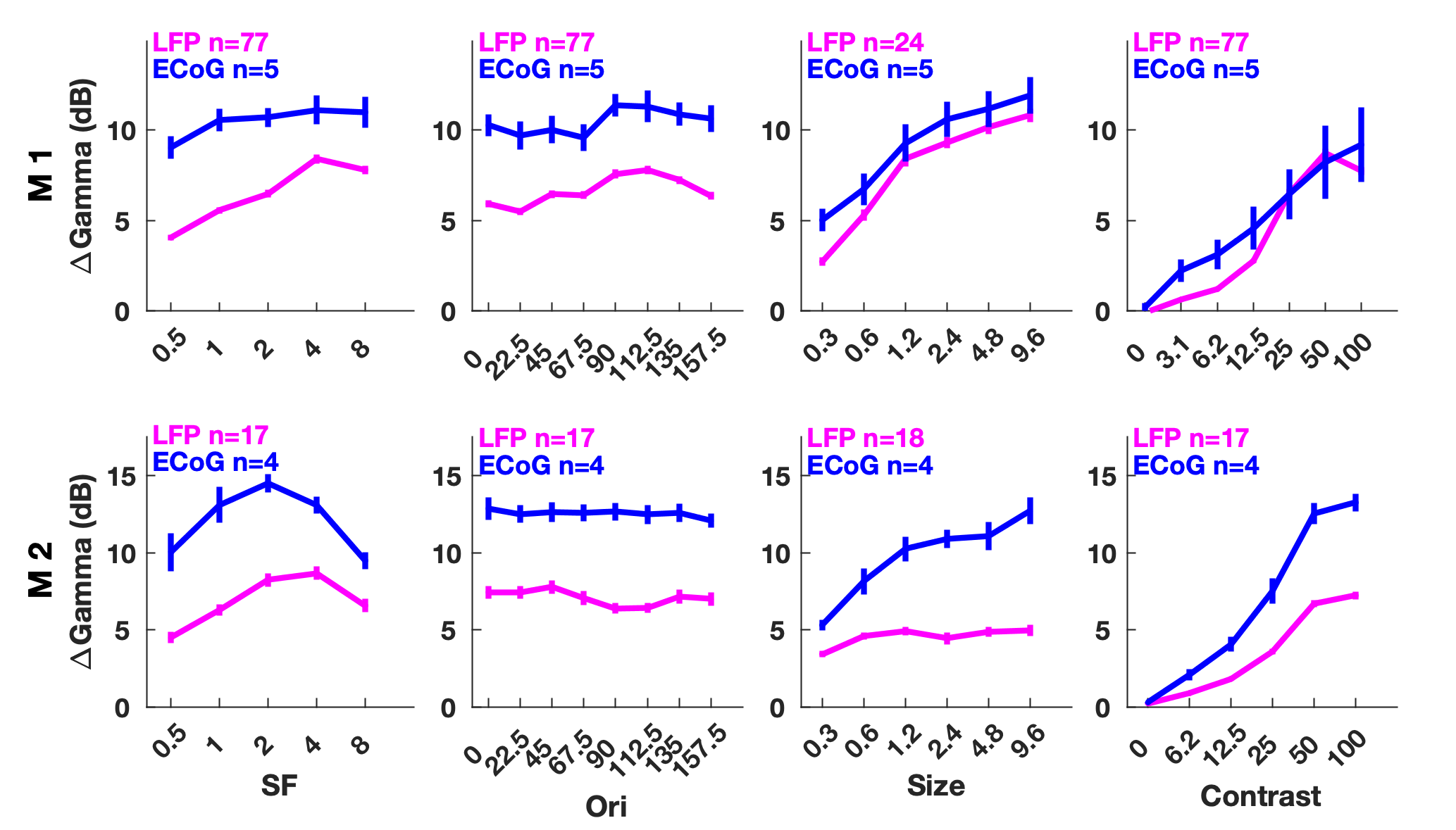

Supplement: Figure 1-1 — Gamma response to grating features. Response refers to the change in gamma band (30-80 Hz) power from baseline (-250-0 ms) in 250-500 ms period, expressed on a log scale in decibels. The spatial frequency (SF) tuning is obtained by averaging over 8 orientations in the SF-Ori data. The orientation tuning uses the same data averaged across 5 SFs. The size response is displayed using Size-Ori data and averaging across 8 orientations, while the contrast response utilizes the Con-Ori data averaged over orientations. The responses are averaged over electrodes (magenta: LFP, blue: ECoG), and error bars denote SEM. Download Figure 1-1, TIF file. [file eneuro-11-ENEURO.0417-23.2024-s002.tif]

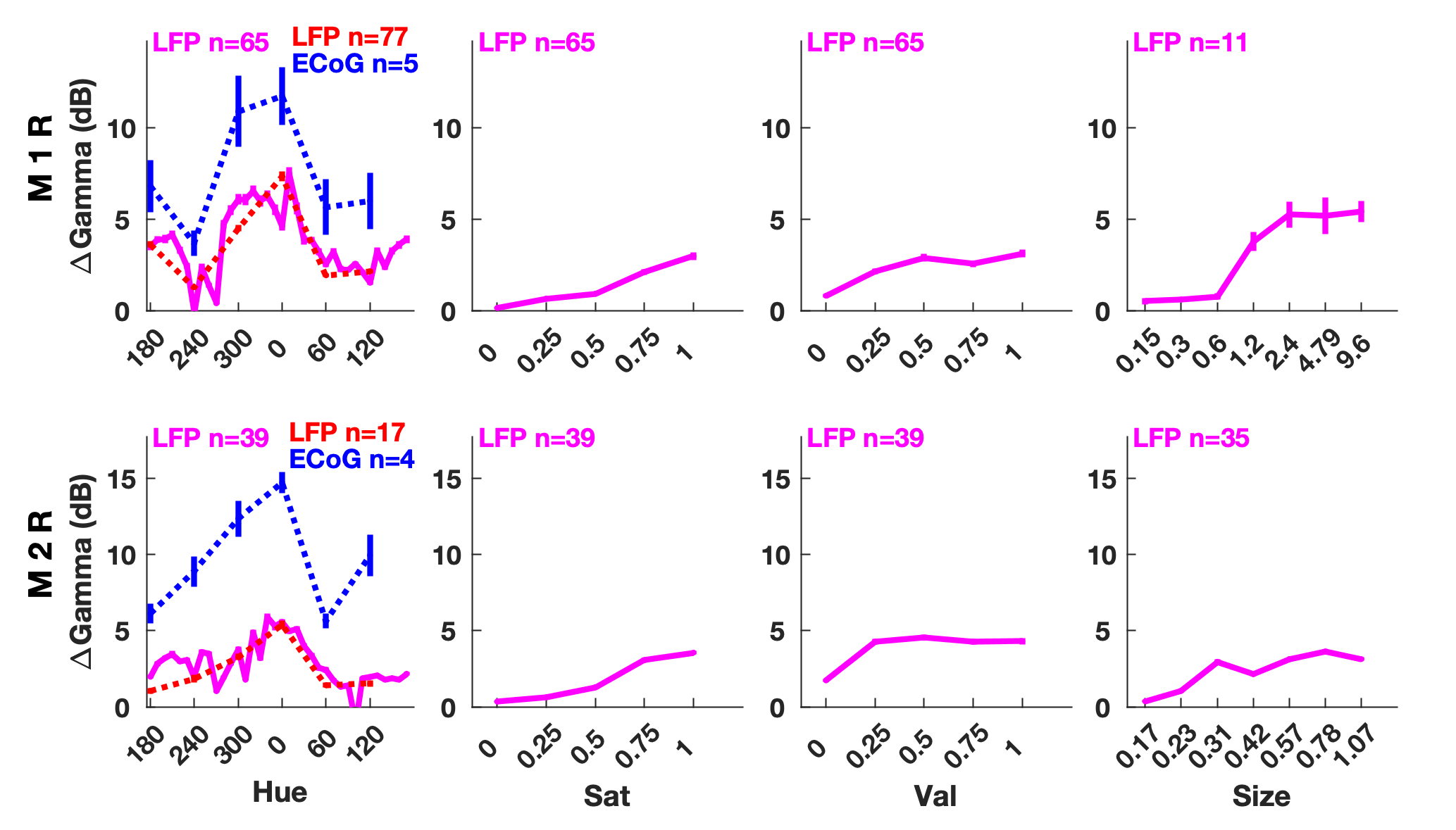

Supplement: Figure 1-2 — Gamma response to chromatic features. Response refers to the change in gamma band (30-80 Hz) power from baseline (-250-0 ms) in 250-500 ms period, expressed on a log scale in decibels. The hue tuning is obtained from the Hue data in which 36 hues were displayed (with 0° signifying red) for M1R and M2R. The dotted lines are for M1 and M2 (same monkeys, other cerebral hemisphere) showing LFP (red) and ECoG (blue) responses to 6 hues. The saturation (Sat) tuning uses the Hue-Sat data averaged across 6 hues, while the value (Val) response utilizes the Hue-Val data averaged over the 6 hues. The Size response is displayed using Hue-Size data for the red hue. All responses are averaged over electrodes (magenta, red: LFP, blue: ECoG), and error bars denote SEM. Download Figure 1-2, TIF file. [file eneuro-11-ENEURO.0417-23.2024-s003.tif]

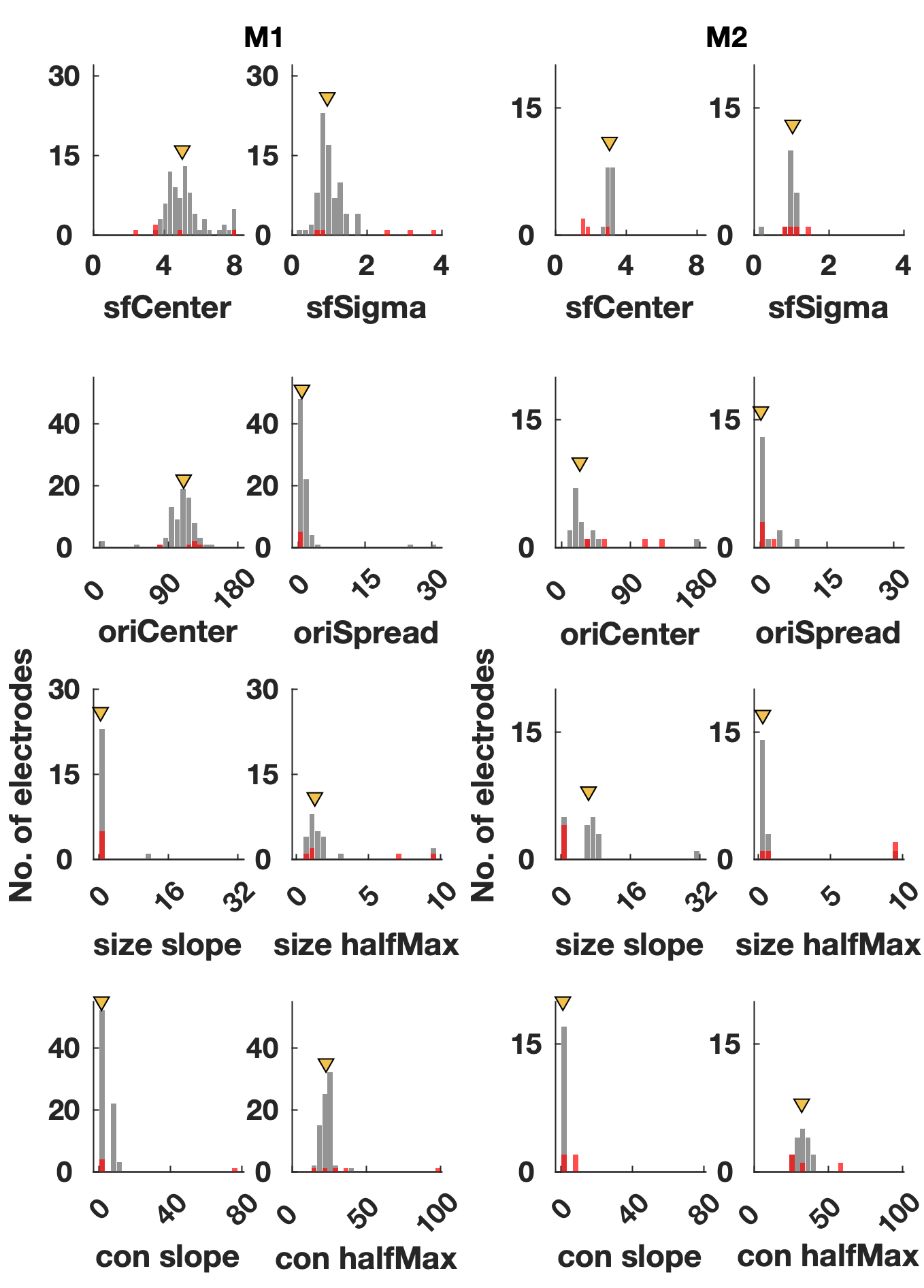

Supplement: Figure 3-1 — Distribution of grating parameters. Distribution of 8 parameters for all electrodes defining the shape of tuning functions to independent features of spatial frequency, orientation, size and contrast, shown for both monkeys. Note that while trials were divided into two halves for testing the model performance, here these parameters were obtained using all trials (averaging the parameters obtained from two halves also yielded similar results). The grey bars correspond to microelectrodes, and red ones to ECoGs. Medians across all electrodes are shown as triangular markers. Download Figure 3-1, TIF file. [file eneuro-11-ENEURO.0417-23.2024-s004.tif]

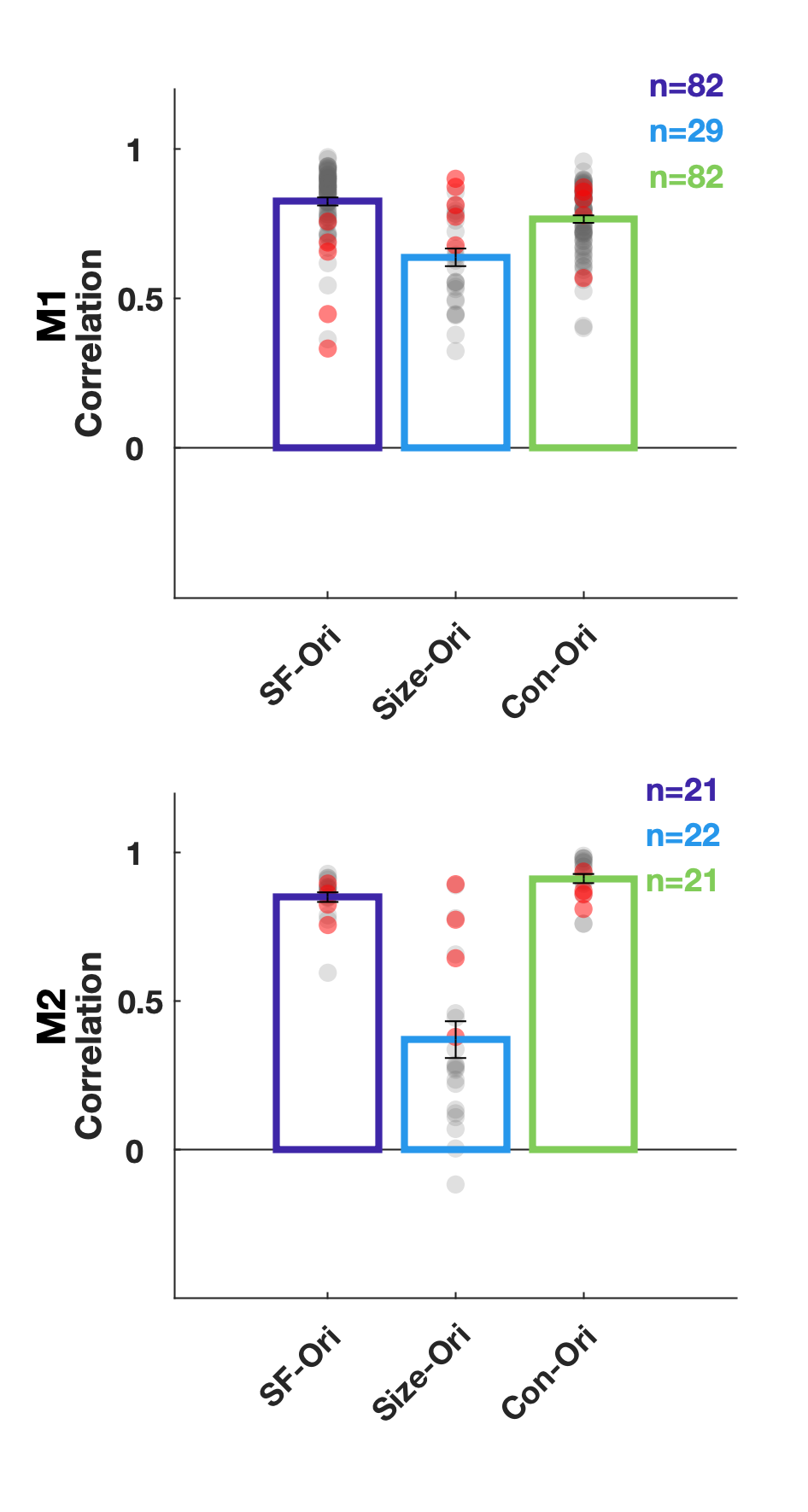

Supplement: Figure 3-2 — Performance with individual parameters. The correlation between estimated and actual gamma responses when individual tuning parameters of electrodes were used instead of medians, for three grating protocols. Red dots correspond to ECoGs and grey to individual microelectrodes. The correlation values of each electrode are averaged over 3 iterations of cross-validation. Error bars correspond to SEM. Download Figure 3-2, TIF file. [file eneuro-11-ENEURO.0417-23.2024-s005.tif]

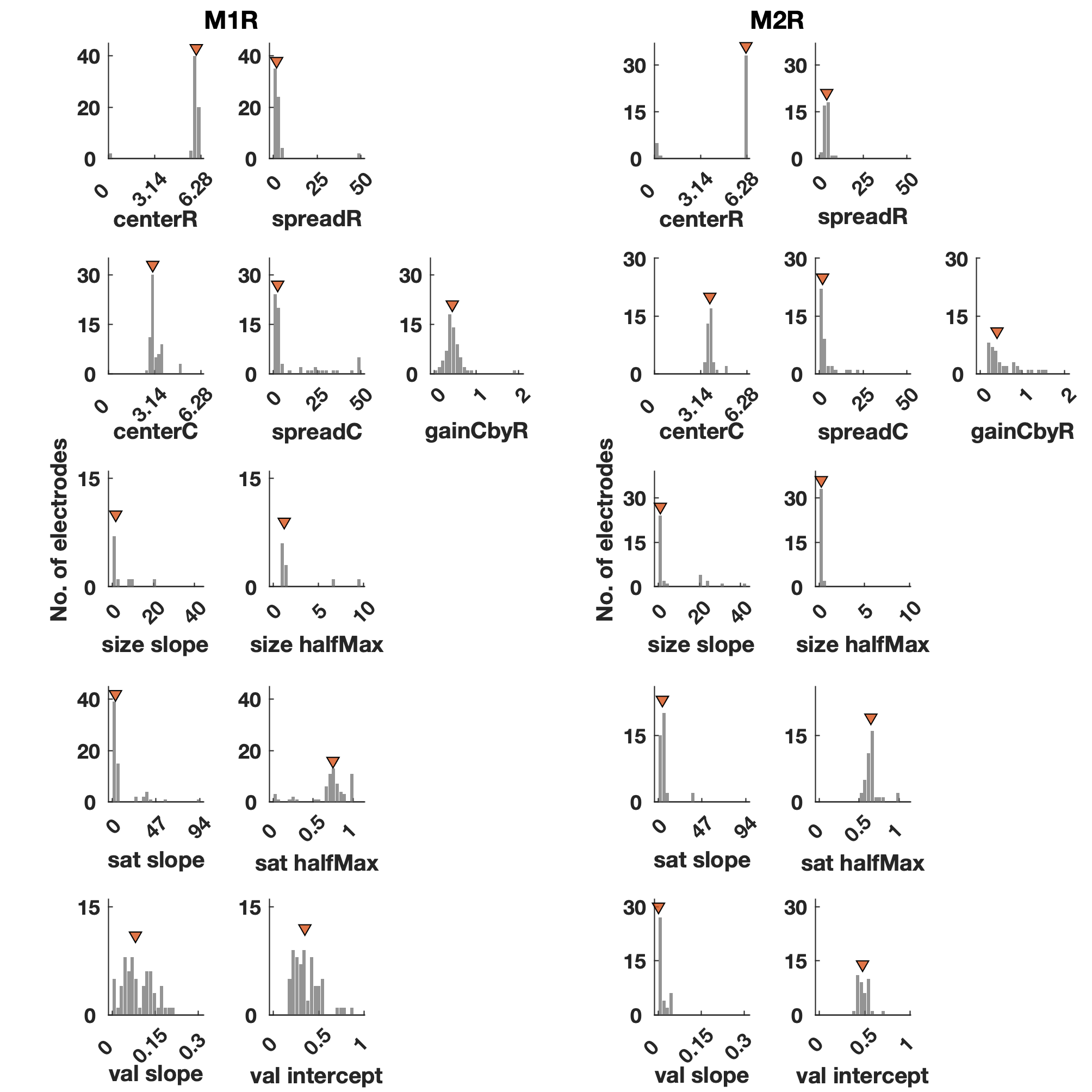

Supplement: Figure 4-1 — Distribution of hue parameters. Distribution of 11 parameters for all electrodes defining the shape of tuning functions to independent features of hue, size, saturation and value, shown for both monkeys. All trials were used to calculate these parameters. Medians across all microelectrodes are shown as triangular markers. Download Figure 4-1, TIF file. [file eneuro-11-ENEURO.0417-23.2024-s006.tif]

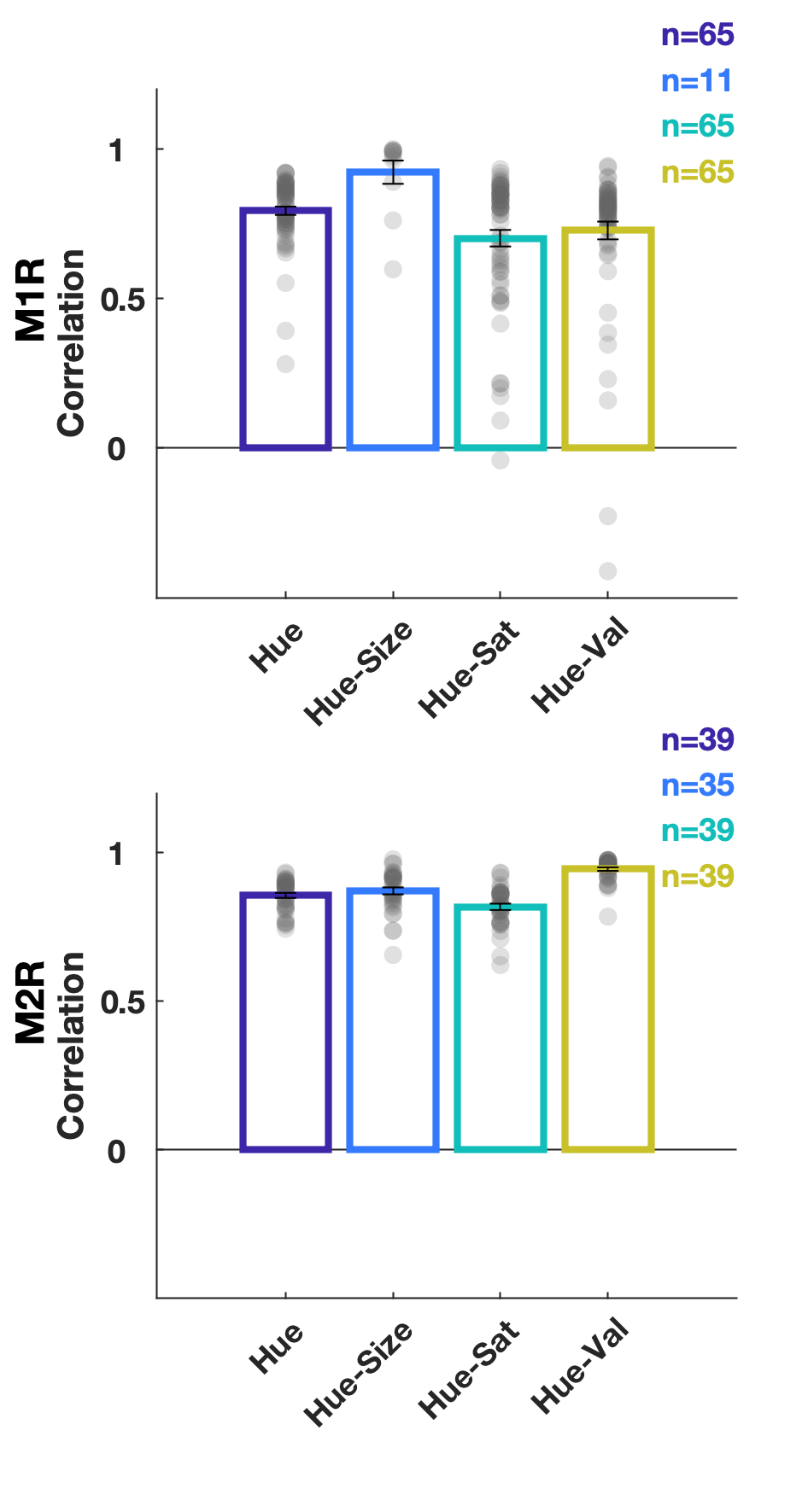

Supplement: Figure 4-2 — Performance with individual parameters. The correlation between estimated and actual gamma responses when individual tuning parameters of electrodes were used instead of medians, for the hue protocols. Grey dots correspond to individual microelectrodes. The correlation values of each electrode are averaged over 3 iterations of cross-validation. Error bars correspond to SEM. Download Figure 4-2, TIF file. [file eneuro-11-ENEURO.0417-23.2024-s007.tif]
